# Whole genome resequencing data for three rockfish species of *Sebastes*

**DOI:** 10.1038/s41597-019-0100-z

**Published:** 2019-06-20

**Authors:** Shengyong Xu, Linlin Zhao, Shijun Xiao, Tianxiang Gao

**Affiliations:** 1 Fishery College, Zhejiang Ocean University, 1st Haidanan Road, Zhoushan, 316022 P.R. China; 2 The First Institute of Oceanography, Ministry of Natural Resources, 6th Xianxialing Road, Qingdao, 266061 P.R. China; 3 School of Computer Science and Technology, Wuhan University of Technology, 122th Luoshi Road, Wuhan, 430070 P.R. China

**Keywords:** Marine biology, DNA sequencing, Comparative genomics, Next-generation sequencing, Ichthyology

## Abstract

Here we report Illumina-based whole genome sequencing of three rockfish species of *Sebastes* in northwest Pacific. The whole genomic DNA was used to prepare 350-bp pair-end libraries and the high-throughput sequencing yielded 128.5, 137.5, and 124.8 million mapped reads corresponding to 38.54, 41.26, and 37.43 Gb sequence data for *S. schlegelii*, *S. koreanus*, and *S. nudus*, respectively. The k-mer analyses revealed genome sizes were 846.4, 832.5, and 813.1 Mb and the sequencing coverages were 45×, 49×, and 46× for three rockfish, respectively. Comparative genomic analyses identified 46,624 genome-wide single nucleotide polymorphisms (SNPs). Phylogenetic analysis revealed closer relationships of the three species, compared to other six rockfish species. Demographic analysis identified contrasting changes between *S. schlegelii* and other two species, suggesting drastically different response to climate changes. The reported genome data in this study are valuable for further studies on comparative genomics and evolutionary biology of rockfish species.

## Abstract (summary)

**Table:** 

Design Type(s)	species comparison design • sequence analysis objective
Measurement Type(s)	whole genome sequencing assay
Technology Type(s)	DNA sequencing
Factor Type(s)	
Sample Characteristic(s)	Sebastes schlegelii • Sebastes koreanus • Sebastes nudus

Machine-accessible metadata file describing the reported data (ISA-Tab format)

## Background & Summary

The rockfish of genus *Sebastes* Cuvier 1829 is the most specious in the family Sebastidae (Actinopterygii: Scorpaeniformes)^1,2^. The genus contains nearly 110 species worldwide and most of the species are subjected to substantial commercial and recreational fisheries^2^. Such great species diversity is likely attributed to recent species diversification processes^2–4^, thus resulting taxonomic confusion in some areas due to morphological similarity. The rockfish species have provided valuable opportunities for evolutionary studies, shedding light on the origin and diversification within the genus^3,5^. In addition, as ovoviviparous teleost, rockfish could provide exceptional clues for studying evolution of their reproductive ecology. Ovoviviparity is a unique fish reproduction mode, in which fertilized eggs cannot be delivered from the female ovary until the embryos are mature^6^. In these respects, molecular information such as whole genome data would contribute to providing more comprehensive insights into evolutionary biology of these species.

In this study, we report whole genome data of three marine ovoviviparous fish in genus *Sebastes*, viz., *Sebastes schlegelii* Hilgendorf 1880, *Sebastes koreanus* Kim and Lee 1994, and *Sebastes nudus* Matsubara 1943. The three rockfish are commercial species commonly distributed in Korea, Japan, and northeast coast of China^1^. Herein, a total of three male adults (each individual representing one species) were collected from coastal waters of Qingdao, China. Prior to sequencing, the genome sizes of three species were estimated as ~800 Mb, thus nearly 40 Gb sequencing data (about 50× genome coverage) of each species was produced by Illumina HiSeq2500 sequencing platform. We intend to develop genomic resources for further studies on taxonomy, phylogenetics, conservation and evolution of these commercially important rockfish in genus *Sebastes*.

**Fig. 1:**
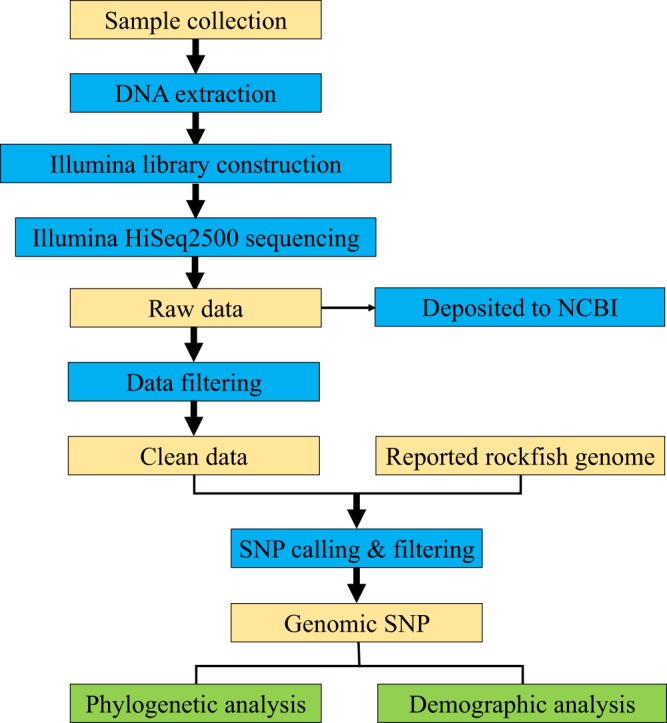
Overview of the experimental design and analysis pipeline.

The experimental design, sequencing and analysis pipeline is shown in Fig. 1. After data filtering, a total of 38.54, 41.26, and 37.43 Gb sequence data were produced for *S. schlegelii*, *S. koreanus*, and *S. nudus*, respectively (Table 1). K-mer analyses revealed the genome size was 846.4, 832.5, and 813.1 Mb for the respective three species (Table 2). The genome sequences of *S. schlegelii*, *S. koreanus*, and *S. nudus* were assembled into scaffolds with a total size of 755.1, 751.7, and 748.5 Mb, respectively. The estimated genomic information of three rockfish species were shown in Table 2.

**Table 1:** Summary of the high-throughput sequencing in this study.

Species	Sample type	Library type	Total length (Gb)	Effective rate (%)	Error rate (%)	Q20 (%)	SRA Accession Number
*Sebastes schlegelii*	DNA	350-bp pair-end	38.54	99.63	0.02	97.53	SRP171394
*Sebastes koreanus*	DNA	350-bp pair-end	41.26	99.63	0.02	97.53	SRP171394
*Sebastes nudus*	DNA	350-bp pair-end	37.43	99.86	0.03	97.42	SRP171394

**Table 2:** Statistical information of k-mer analysis and genome assembly in this study.

Species	Gen (Mb)	Cov (X)	Het (%)	Rep (%)	No_cont_	N50_cont_ (bp)	L_cont_ (Kb)	No_scaf_	N50_scaf_ (bp)	L_scaf_ (Kb)	L_total_ (Mb)
*Sebastes schlegelii*	846.36	45	0.22	44.10	379,992	6,790	86.78	260,971	13,978	207.70	755.13
*Sebastes koreanus*	832.53	49	0.20	43.65	385,402	7,261	109.96	259,176	16,225	269.69	751.68
*Sebastes nudus*	813.12	46	0.31	41.40	402,857	6,813	109.92	318,031	10,934	165.86	748.46

Note: Gen: genome size, Cov: sequencing coverage, Het: Heterozygous ratio, Rep: Repeat ratio, No_cont_: number of contigs, N50_cont_: contig N50, L_cont_: maximum length of contigs, No_scaf_: number of scaffolds, N50_scaf_: scaffold N50, L_scaf_: maximum length of scaffolds, L_total_: total length of scaffolds.

**Fig. 2:**
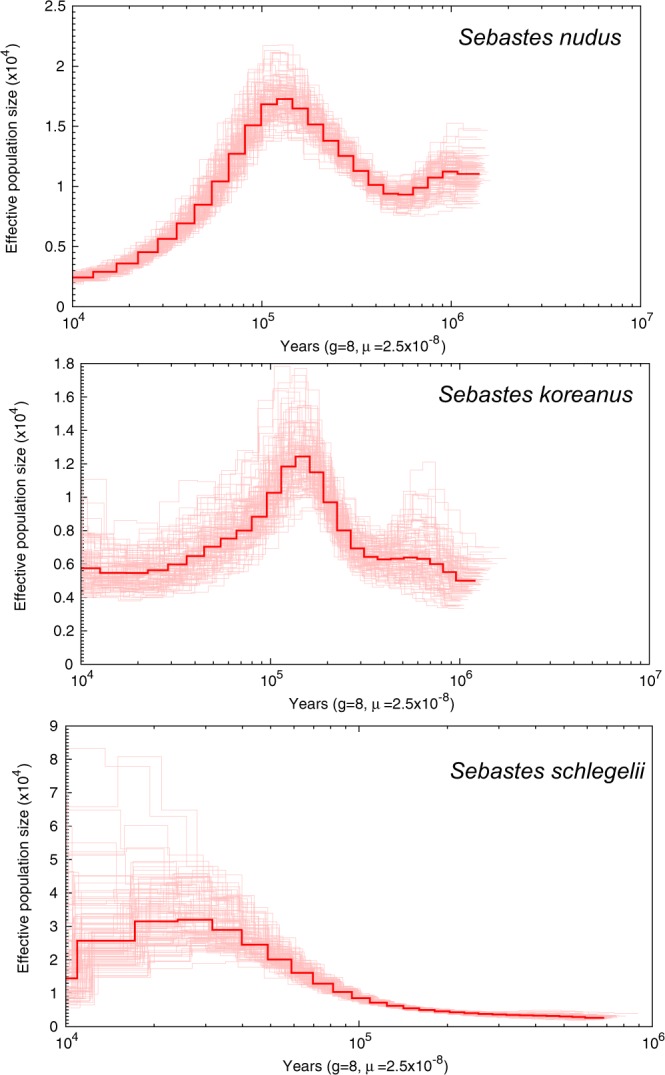
Demographic history of three rockfish species in this study. PSMC estimates of demographic changes in effective population size (*N*e) over time inferred from the draft genome sequences of the three rockfishes. Thick lines represent the median and thin light lines correspond to 100 rounds of bootstrapping.

**Fig. 3:**
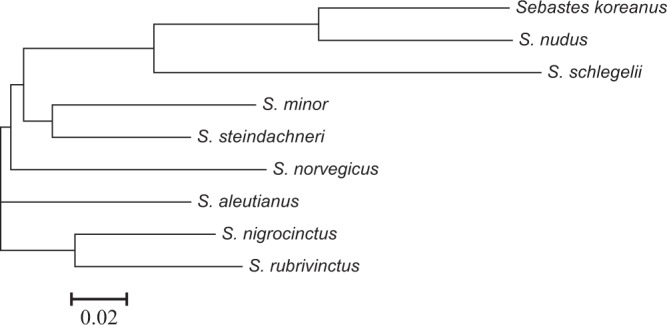
Phylogenetic relationship reconstructed based on whole genome sequences of nine rockfish species. The whole genome sequences of 9 rockfish species (including 6 reported species and 3 species in this study) were used for phylogenetic reconstruction based on neighbour-joining algorithm.

The filtered clean data were mapped to the reported *S. steindachneri* (GCA_001910785.2) reference genome and the generated bam files were subsequently investigated in demographic analyses. A coalescent-based hidden Markov model, the pairwise sequentially Markovian coalescent (PSMC) model, was used to infer the history of effective population sizes (*N*e). The PSMC results exhibited contrasting demographic changes in the last glacial, revealing *N*e increase in *S. schlegelii* and decrease in other two species (Fig. 2). The demographic analyses suggested that drastically different responses to climate changes can be detected in closely related species, as reported in demographic changes of two closely related dolphin species^7^. Such contrasting demographic changes could be due to the altered ecology of competitors and the pattern of population differentiation^7^. Further studies are warranted to specify the contrasting demographic patterns among closely related species. In addition, phylogenetic relationship of species in genus *Sebastes* were reconstructed based on whole genome sequences. Supplemented with six reported genome sequences, a total of 14,821,089 single nucleotide polymorphisms (SNPs) were identified. After SNP filtering, the remaining 46,624 SNPs were employed in phylogenetic reconstruction. The neighbour-joining topology revealed closer relationship of *S. schlegelii*, *S. koreanus*, and *S. nudus*, compared to other rockfish species in this genus (Fig. 3). Based on a literature survey and author knowledge, the reported whole genome data in the present study is the first whole genome information present to the public of the three rockfish, therefore, these data could be valuable for further studies on taxonomy, phylogenetics and evolutionary biology of rockfish species.

## Methods

### Sample collection

Animal experiments were conducted in accordance with the guidelines approved by the Zhejiang Ocean University Animal Ethics Committee and the national legislation. The sample collection procedure was following the description of our previous published work (ref.^8^). To obtain enough genomic DNA for the Illumina sequencing, we collected fresh epaxial white muscle tissues from *Sebastes schlegelii*, *S. koreanus*, and *S. nudus* sampled from Qingdao, China. The samples were quickly frozen in liquid nitrogen for 1 hour before storing at −80 °C. Genomic DNA was extracted using a standard phenol/chloroform extraction protocol. The integrity of genomic DNA molecules was checked using agarose gel electrophoresis, showing a main band around 20 Kb and satisfying the requirement for Illumina library construction by the manufacturer’s protocol.

### Whole-genome sequencing

Whole genome sequencing was performed commercially at Novogene Co. Ltd in Beijing. In brief, 1.0 μg of genome DNA was fragmented using an E210 Focused-ultrasonicator (Covaris, Woburn, MA). The sheared DNA fragments were used to prepare pair-end libraries with an average insert size of 350 bp for all samples according to the manufacturer’s instructions (Illumina Inc., San Diego, CA). Each library was sequenced in two independent lanes of HiSeq 2500 platform (Illumina Inc.) using 150-bp pair-end fashion. The raw data were converted to single-sample FASTQ files through base calling procedure and after filtering interference information such as adaptors and low-quality reads, the clean data FASTQ files of each sample were employed for further bioinformatics analyses.

### Genome assembly

The genome size, heterozygous ratio and repeat ratio were estimated using k-mer analysis (K = 17) performed in GCE v1.0.0^9^. Pair-end reads were assembled into contigs and scaffolds in SOAPdenovo v2.01^10^ with a k-mer of 41 by applying the *de Bruijn* graph structure.

### Phylogenetic analysis

The generated genome data were supplemented with publicly available sequences of six rockfish species in genus *Sebastes*, i.e. *S. steindachneri* (GCA_001910785.2), *S. aleutianus* (GCA_001910805.2), *S. minor* (GCA_001910765.2), *S. nigrocinctus* (GCA_000475235.3), *S. norvegicus* (GCA_900302655.1), and *S. rubrivinctus* (GCA_000475215.1) downloaded from NCBI database. The clean reads were aligned to the genome reference of *S. steindachneri* by using the bwa-mem algorithm in BWA 0.7.12^11^ with default parameters. Single nucleotide polymorphisms (SNPs) calling was implemented in SAMtools 1.3.1^12^ with default parameters. SNP filtering was produced using VCFtools^13^. The SNP calling procedure and parameters are expanded versions of descriptions in our related work^14^. In order to avoid sex bias affecting topological structure, contigs containing SNPs were cross-validated with the sex-determining loci identified in the previous study^15^. Sex-determining SNP loci were excluded in phylogenetic analysis. Phylogenetic tree of the nine species of *Sebastes* based on the filtered SNPs was reconstructed using neighbour-joining (NJ) method in Tassel 5^16^ with default parameters. However, potential sampling bias should be raised as a caveat when performing phylogenetic analyses based on SNPs derived from one single individual per species. Further analyses are warranted to obtain more robust results by sampling more individuals.

### Demographic analysis

Analysis of demographic history for all three rockfish species was done using the PSMC model, as implemented in the PSMC package^17^. The “fq2psmcfa” and “splitfa” tools from the PSMC package were used to create the input file for the PSMC modelling. The PSMC analysis command included the options “-N25” for the number of cycles of the algorithm, “-t15” as the upper limit for the most recent common ancestor (TMRCA), “-r5” for the initial θ/ρ, and “-p 4 + 25*2 + 4 + 6” atomic intervals. The reconstructed population history was plotted using “psmc_plot.pl” script using the substitution rate “-u 2.5e-8” adopted from medaka^18^, and a generation time of 8 years. The generation time was calculated as: g = a + [s/(1 − s)]^19^, where s is the expected adult survival rate which is assumed as 80%, and a is sexual maturation age that is 4 years for *S. schlegelii*^20^. Therefore, the generation time was determined as 8 in the PSMC analysis. To determine variance in the estimated effective population size, we performed 100 bootstraps for each species.

## Data Records

All sequencing raw reads for the three rockfish species have been deposited within NCBI Sequence Read Archive^21^, and the assembly genome sequences (*Sebastes schlegelii*^22^, *S. nudus*^23^, and *S. koreanus*^24^) have been deposited within GenBank. Also, the assembly genome sequences, aligned VCF files and phylogenetic tree file were stored in Figshare^25^.

## Technical Validation

**Fig. 4:**
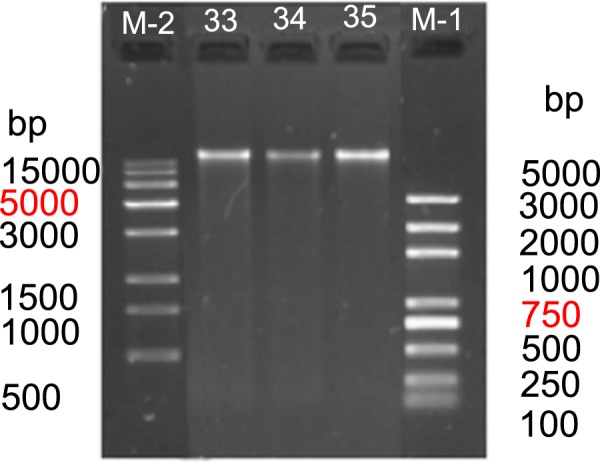
Agarose gel electrophoresis of DNA integrity assessment. The DNA lanes presented here have been cropped from a larger image with multiple DNA samples. Two kinds of DNA markers (M-1 and M-2) were used for DNA integrity assessment. Numbers embedded in the diagram (33, 34, and 35) represent *S. schlegelii*, *S. koreanus*, and *S. nudus*, respectively.

**Fig. 5:**
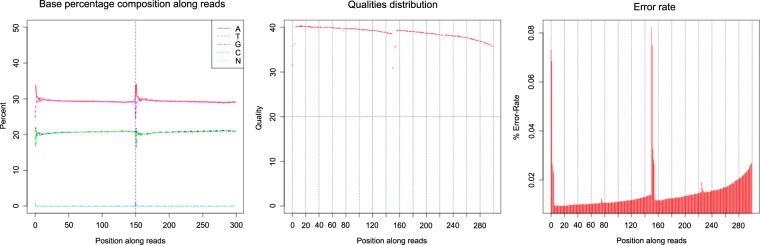
Quality evaluation including base composition, quality scoring and error rate of sequencing data. Sequencing quality met the requirement of further bioinformatics analyses in all three species. Illustrated here by the example of *S. nudus*.

In our present study, the sampled fish individuals were captured using hook-and-line fishing in the coastal waters of Qingdao, China. Taxonomic determination was implemented in the laboratory by identifying morphological characters. The DNA quality was checked using agarose gel electrophoresis (Fig. 4). The preprocessing steps including quality evaluation and data filtering of raw reads were implemented by the following procedures as in the previous study^8^. The quality of raw reads was evaluated using FastQC^26^ software and low-quality reads were filtered using HTQC^27^ software according to the following criteria: (1) adaptors in the reads were trimmed and removed; (2) read pairs were removed when either of the reads had more than 10% of N bases; (3) read pairs were removed if either of the reads had more than 20% low-quality bases (phred quality score < 5); (4) ambiguous or low-quality fragments at the two ends of reads within a window size of 5 bp and an average quality threshold of 20 were trimmed. The sequencing quality was also assessed by examining GC-content, Q20-statistics and error rate (Table 1, Fig. 5). FastQC output files can be also viewed within the Supplementary Information. Moreover, the parameters used in bioinformatics analyses were following the default settings or the published literatures, which were provided in the *Methods* section.

## Supplementary Information

### ISA-Tab metadata file

Download metadata file

### Supplementary information

Supplementary Information

## Data Availability

The code used at each step were shown in the respective methods.

## References

[1] Nelson, J. S., Grande, T. C. & Wilson, M. V. H. *Fishes of the world*, 5th edition. (John Wiley & Sons, 2016).

[2] Kai Y, Nakabo T (2013). Taxonomic review of the *Sebastes pachycephalus* complex (Scorpaeniformes: Scorpaenidae). Zootaxa.

[3] Muto N, Kai Y, Nakabo T (2019). Taxonomic review of the *Sebastes vulpes* complex (Scorpaeniformes: Scorpaenidae). Ichthyol. Res..

[4] Kai Y, Park KD, Nakabo T (2012). The incomplete history of mitochondrial lineages between two rockfishes, *Sebastes longispinis* and *Sebastes hubbsi* (Scorpaeniformes: Scorpaenidae). J. Fish Biol.

[5] Ingram T, Kai Y (2014). The geography of morphological convergence in the radiation of Pacific *Sebastes* rockfishes. Am. Nat..

[6] Lyu LK (2018). Deep transcriptomic analysis of black rockfish (*Sebastes schlegelii*) provides new insights on responses to acute temperature stress. Sci. Rep..

[7] Vijay N (2018). Population genomic analysis reveals contrasting demographic changes of two closely related dolphin species in the last glacial. Mol. Biol. Evol..

[8] Xu SY (2018). A draft genome assembly of the Chinese sillago (*Sillago sinica*), the first reference genome for Sillaginidae fishes. GigaScience.

[9] Liu, B. *et al*. Estimation of genomic characteristics by analyzing k-mer frequency in *de novo* genome projects. Preprint at, https://arxiv.org/abs/1308.2012 (2013).

[10] Luo RB (2012). SOAPdenovo2: an empirically improved memory-efficient short-read *de novo* assembler. GigaScience.

[11] Li H, Durbin R (2009). Fast and accurate short read alignment with Burrows-Wheeler Transform. Bioinformatics.

[12] Li H (2009). The sequence alignment/map format and SAMtools. Bioinformatics.

[13] Danecek P (2011). The variant call format and VCFtools. Bioinformatics.

[14] Xu SY (2017). Genomic evidence for local adaptation in the ovoviviparous marine fish *Sebastiscus marmoratus* with a background of population homogeneity. Sci. Rep..

[15] Fowler B, Buonaccorsi V (2016). Genomic characterization of sex-identification markers in *Sebastes carnatus* and *Sebastes chrysomelas* rockfishes. Mol. Ecol..

[16] Bradbury PJ (2007). TASSEL: software for association mapping of complex traits in diverse samples. Bioinformatics.

[17] Li H, Durbin R (2011). Inference of human population history from individual whole-genome sequences. Nature.

[18] Spivakov M (2014). Genomic and phenotypic characterization of a wild medaka population: towards the establishment of an isogenic population genetic resource in fish. G3-Genes Genom. Genet..

[19] Yuan Z (2018). Historical demography of common carp estimated from individuals collected from various parts of the world using the pairwise sequentially markovian coalescent approach. Genetica.

[20] Chen DG (1994). Preliminary studies on biology and cultivation of *Sebastes schlegelii*. Acta Oceanologica Sinica.

[21] *NCBI Sequence Read Archive*, https://identifiers.org/ncbi/insdc.sra:SRP171394 (2018).

[22] Xu, S. Y. *et al*. *Sebastes schlegelii* isolate SS_1, whole genome shotgun sequencing project. *GenBank*, https://identifiers.org/ncbi/insdc:RZNK00000000 (2018).

[23] Xu, S. Y. *et al*. *Sebastes nudus* isolate SN_1, whole genome shotgun sequencing project. *GenBank*, https://identifiers.org/ncbi/insdc:RZNL00000000 (2018).

[24] Xu, S. Y. *et al*. *Sebastes koreanus* isolate SK_1, whole genome shotgun sequencing project. *GenBank*, https://identifiers.org/ncbi/insdc:RZNM00000000 (2018).

[25] Xu, S. Y. *et al*. The assembly draft genome sequences of three rockfish species and filtered SNP matrix. *figshare*, 10.6084/m9.figshare.7409315 (2018).

[26] Andrews, S. *FastQC A quality control tool for high throughput sequence data*, http://www.bioinformatics.babraham.ac.uk/projects/fastqc/ (2013).

[27] Yang X (2013). HTQC: a fast quality control toolkit for Illumina sequencing data. BMC Biol..

