# Supplementary material for: Whole genome resequencing data for three rockfish species of Sebastes
Source: Sci Data. 2019 Jun 20;6:97. doi: 10.1038/s41597-019-0100-z (PMC6586840; doi:10.1038/s41597-019-0100-z)
Supplement: Supplementary file 2 — Supplementary Information [file 41597_2019_100_MOESM2_ESM.pdf]

Supplementary Information

Title
Whole genome resequencing data for three rockfish species of *Sebastes*

Authors
Shengyong Xu, Linlin Zhao, Shijun Xiao, Tianxiang Gao

Contents:

Page 2 to 21: FastQC output HTML files of *Sebastes schlegelii* pair-end clean data

Page 22 to 41: FastQC output HTML files of *Sebastes koreanus* pair-end clean data

Page 42 to 61: FastQC output HTML files of *Sebastes nudus* pair-end clean data

FastQC Report

星期三 8 五月 2019
SS_L4_1_clean.fq.gz

Summary

- Basic Statistics
- Per base sequence quality
- Per tile sequence quality
- Per sequence quality scores
- Per base sequence content
- Per sequence GC content
- Per base N content
- Sequence Length Distribution
- Sequence Duplication Levels
- Overrepresented sequences
- Adapter Content
- Kmer Content

Basic Statistics

Measure	Value
Filename	SS_L4_1_clean.fq.gz
File type	Conventional base calls
Encoding	Sanger / Illumina 1.9
Total Sequences	128134474
Sequences flagged as poor quality	0
Sequence length	150
%GC	40

Per base sequence quality

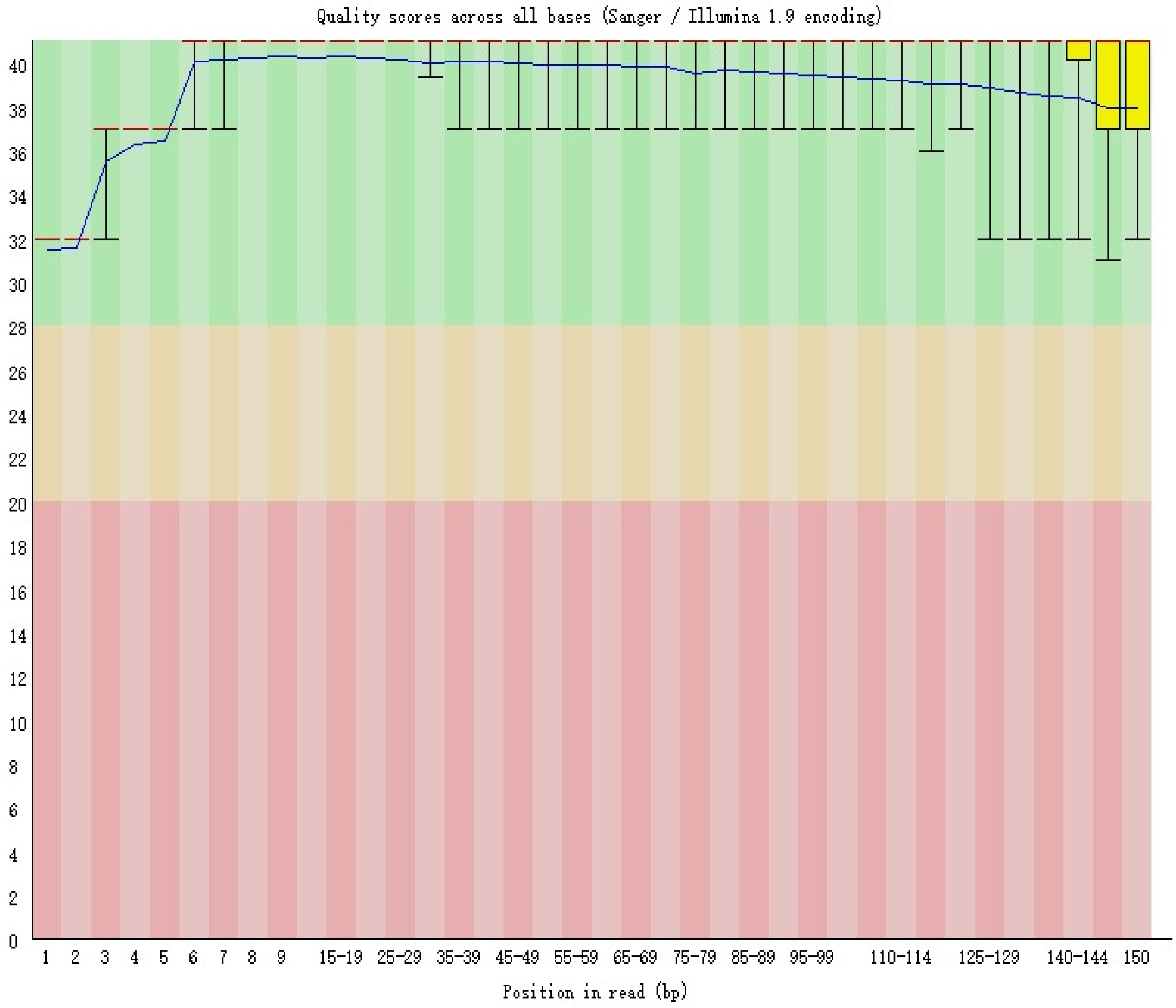

Per tile sequence quality

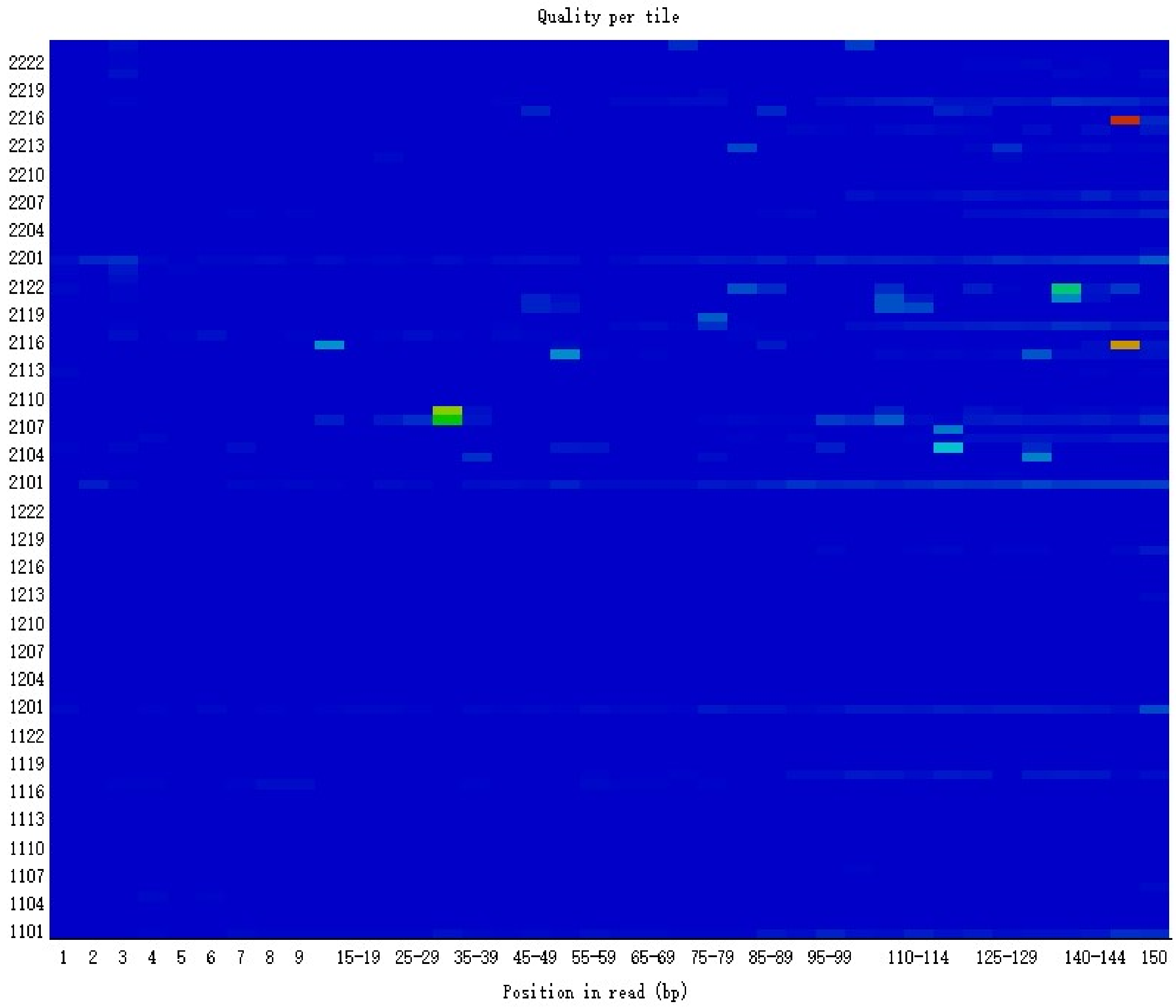

Per sequence quality scores

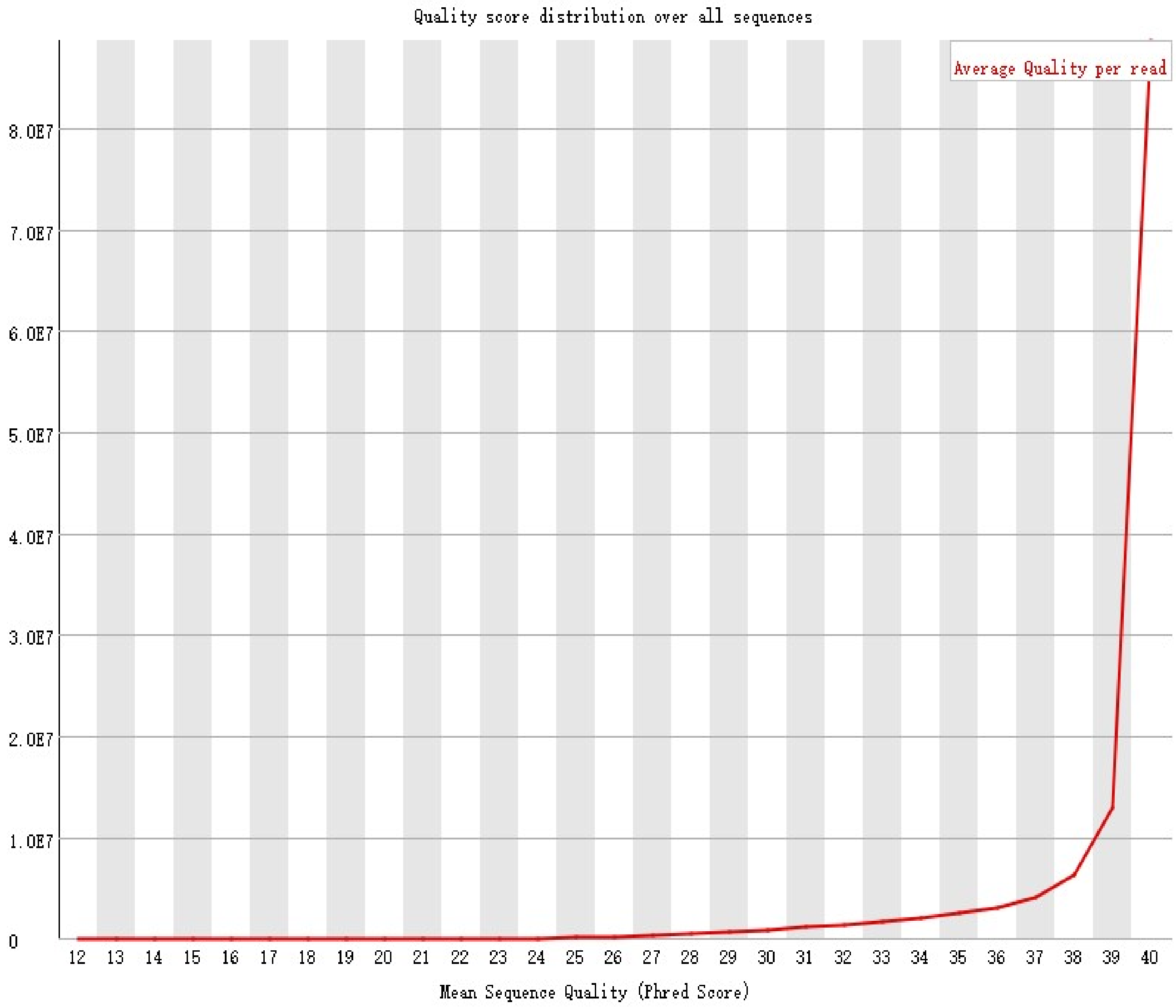

Per base sequence content

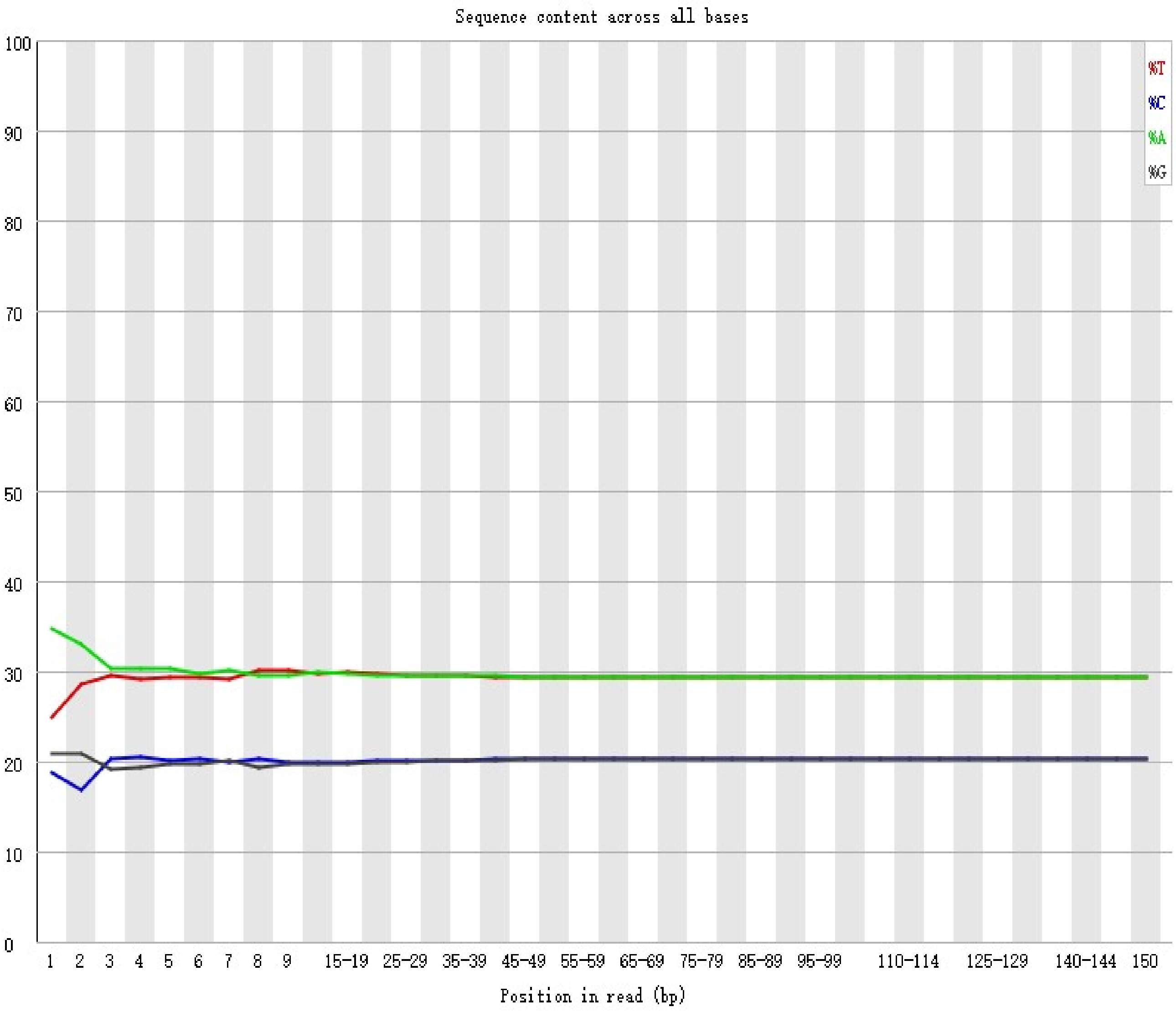

Per sequence GC content

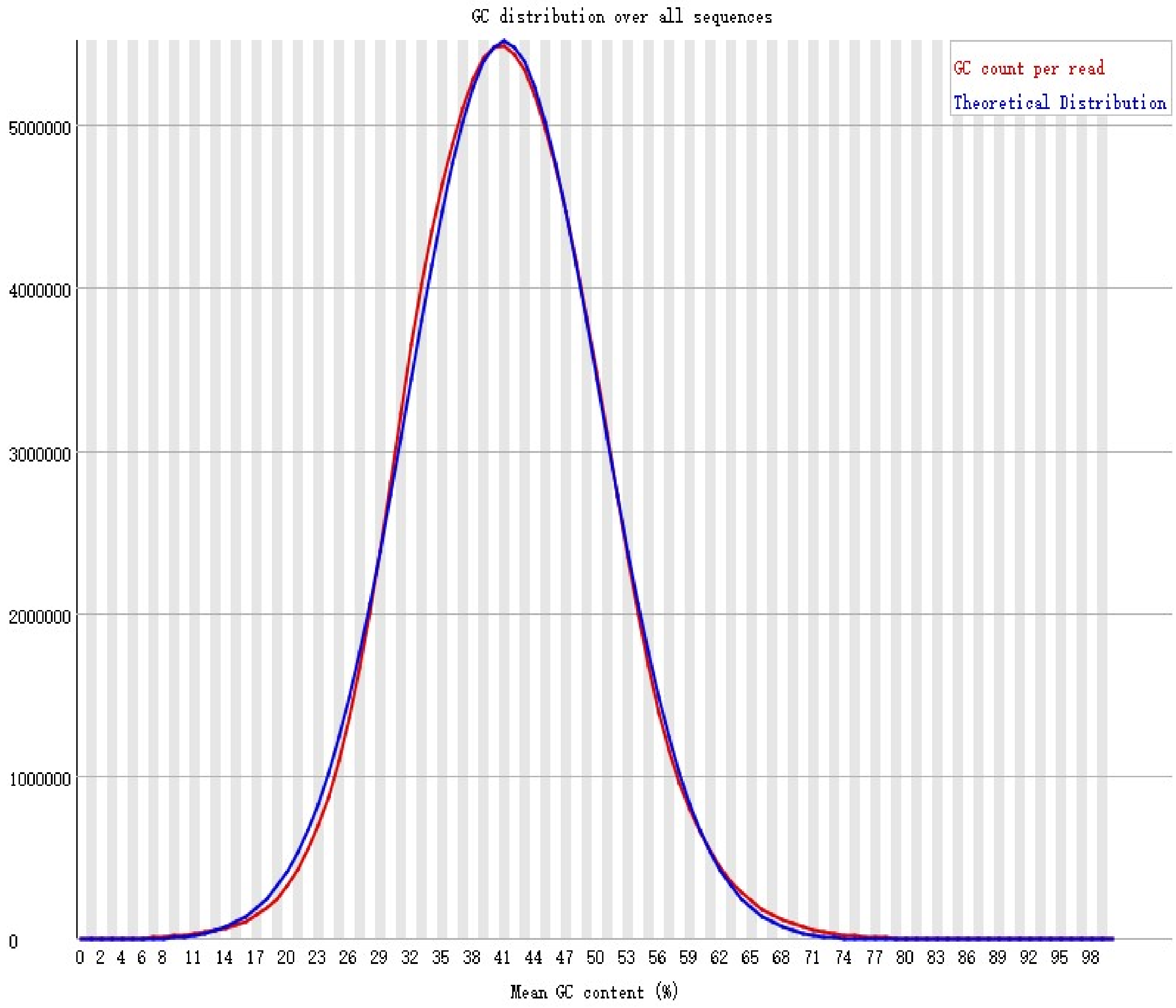

Per base N content

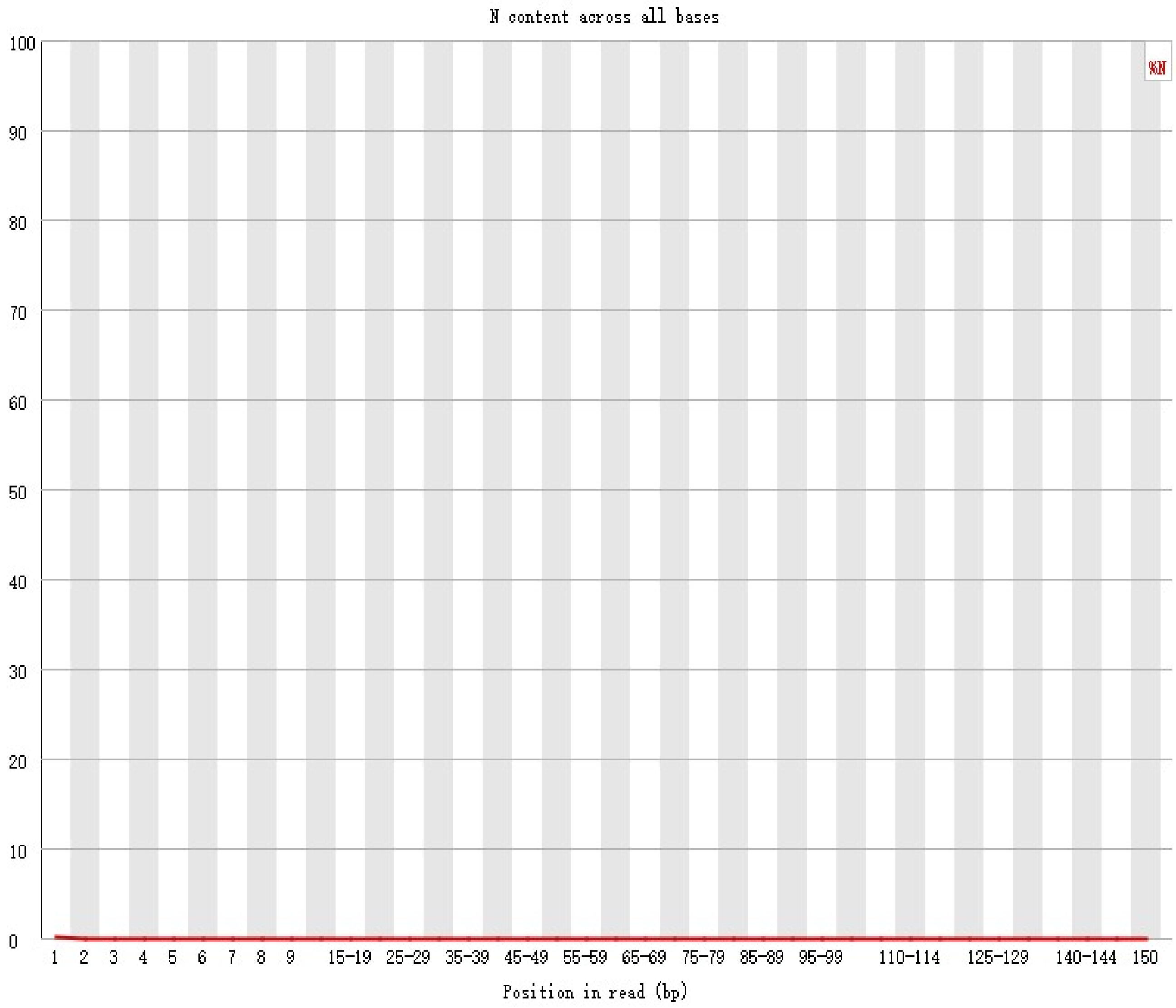

Sequence Length Distribution

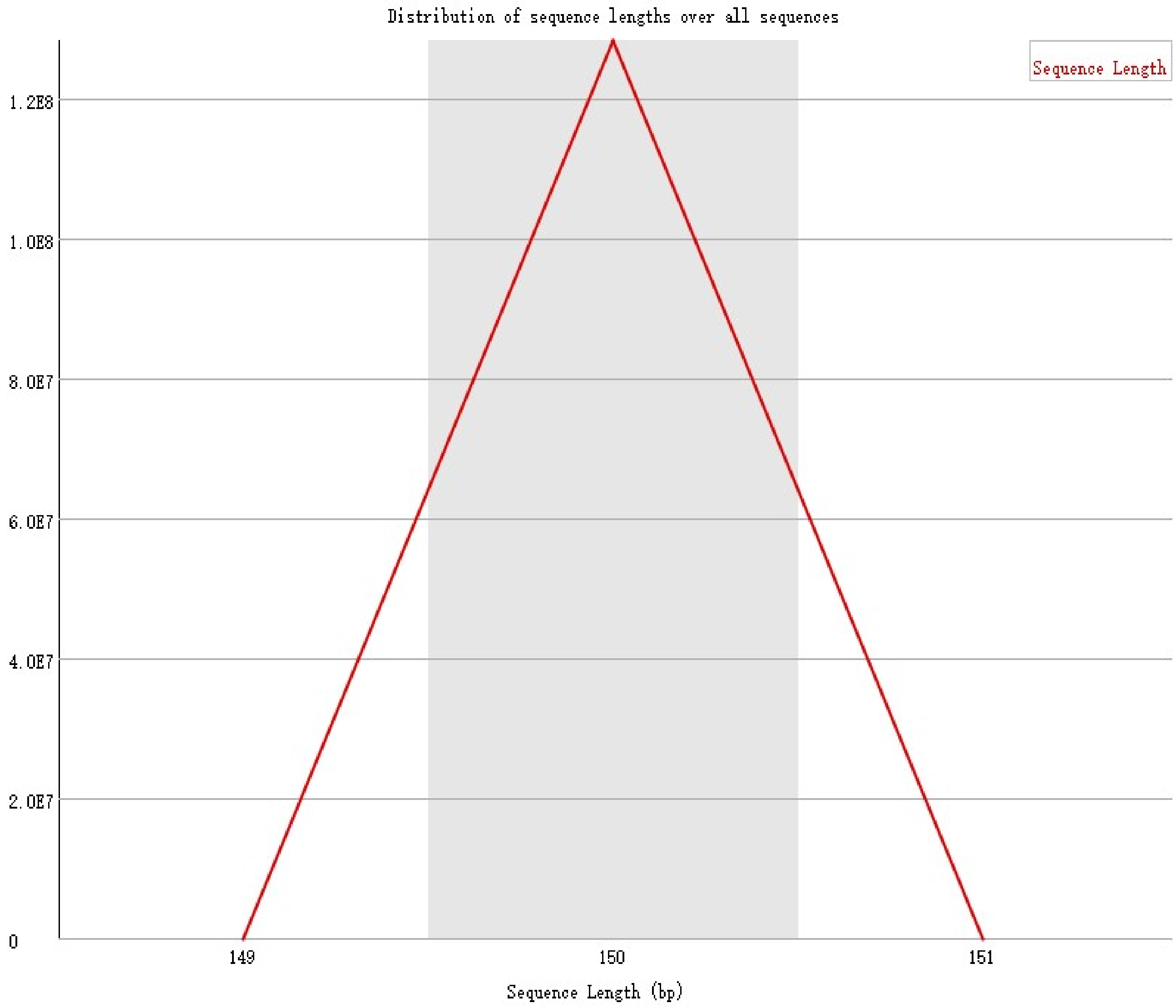

Sequence Duplication Levels

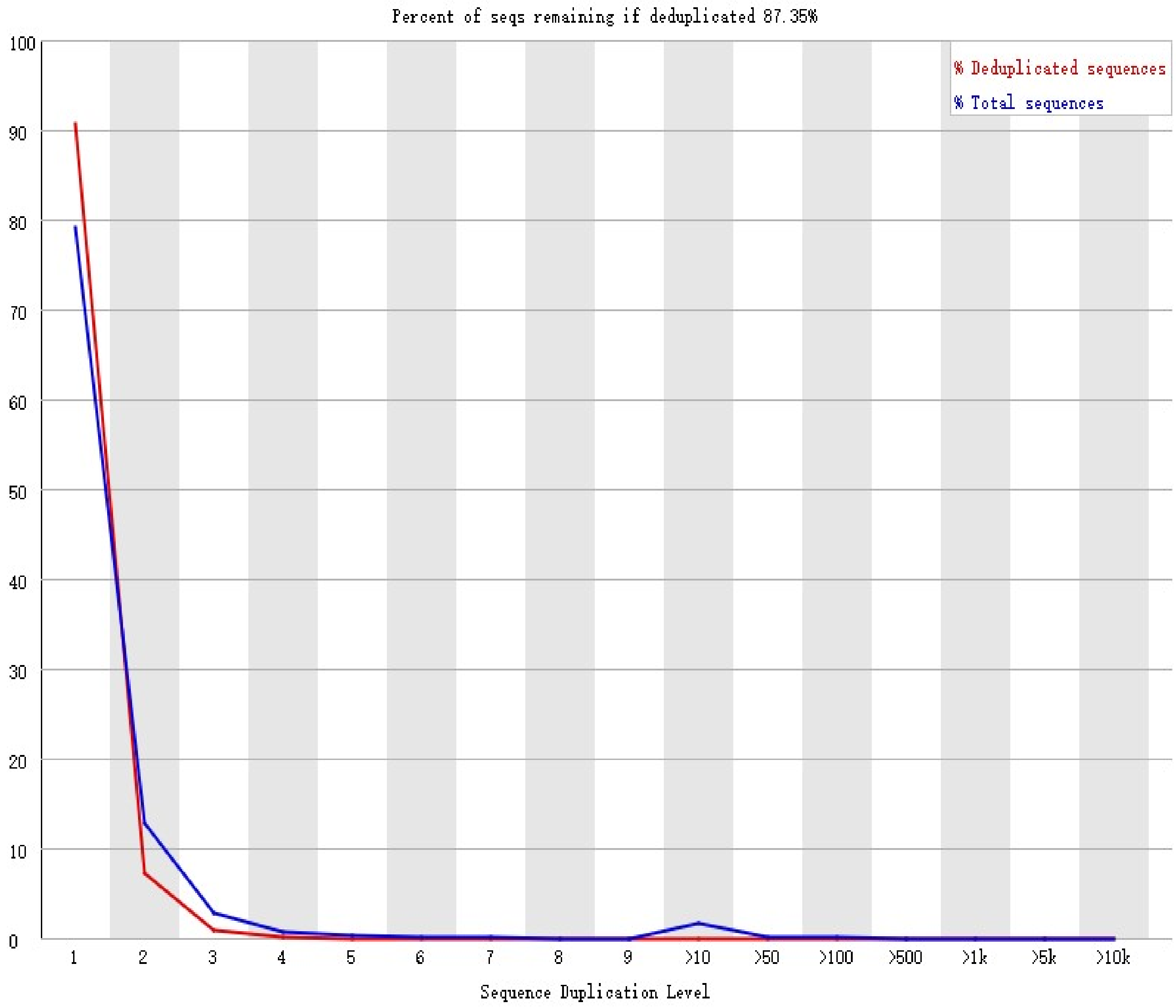

Overrepresented sequences

No overrepresented sequences

Adapter Content

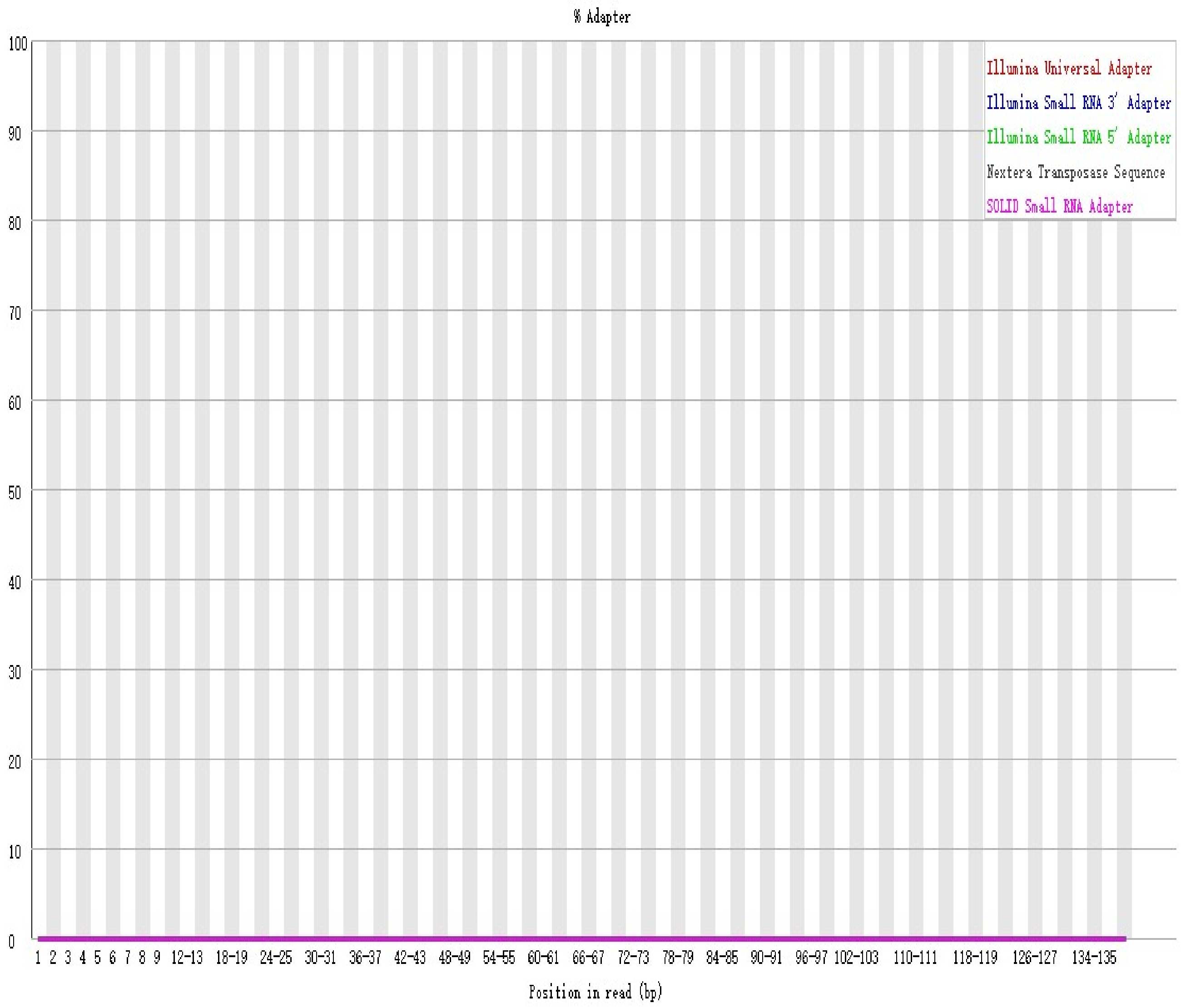

Kmer Content

No overrepresented Kmers

Produced by FastQC (version 0.11.5)

FastQC Report

星期三 8 五月 2019
SS_L4_2_clean.fq.gz

Summary

- Basic Statistics
- Per base sequence quality
- Per tile sequence quality
- Per sequence quality scores
- Per base sequence content
- Per sequence GC content
- Per base N content
- Sequence Length Distribution
- Sequence Duplication Levels
- Overrepresented sequences
- Adapter Content
- Kmer Content

Basic Statistics

Measure	Value
Filename	SS_L4_2_clean.fq.gz
File type	Conventional base calls
Encoding	Sanger / Illumina 1.9
Total Sequences	128134474
Sequences flagged as poor quality	0
Sequence length	150
%GC	40

Per base sequence quality

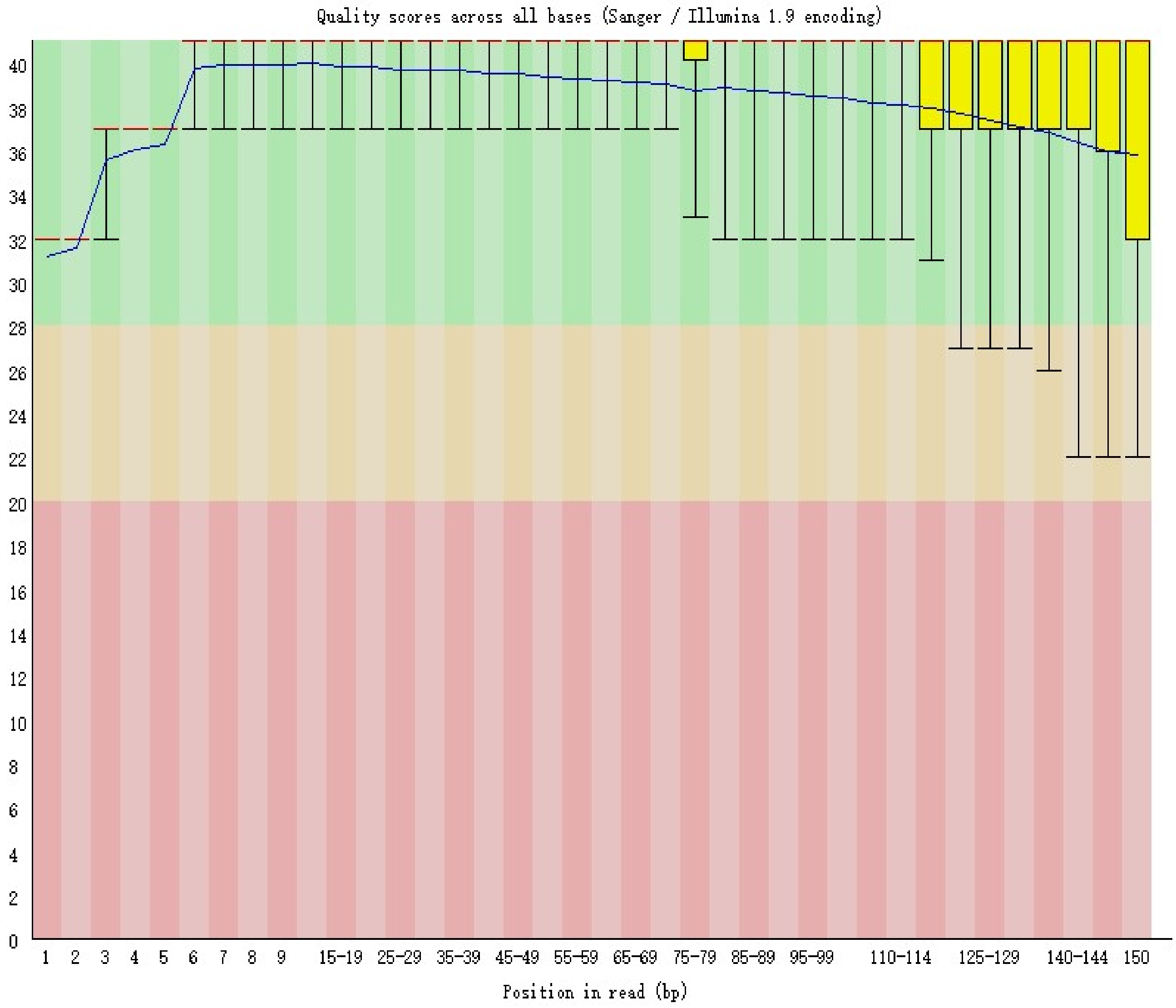

Per tile sequence quality

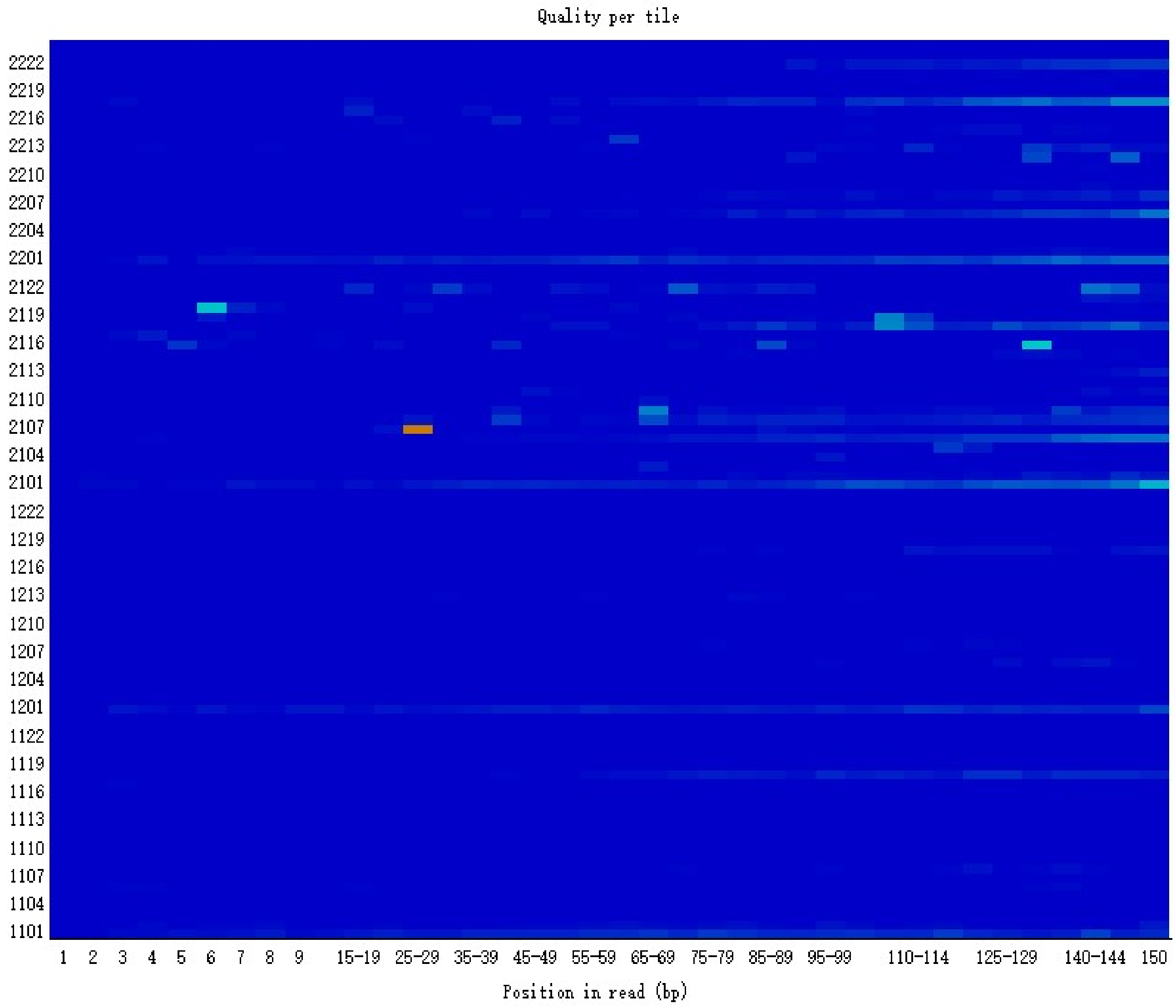

Per sequence quality scores

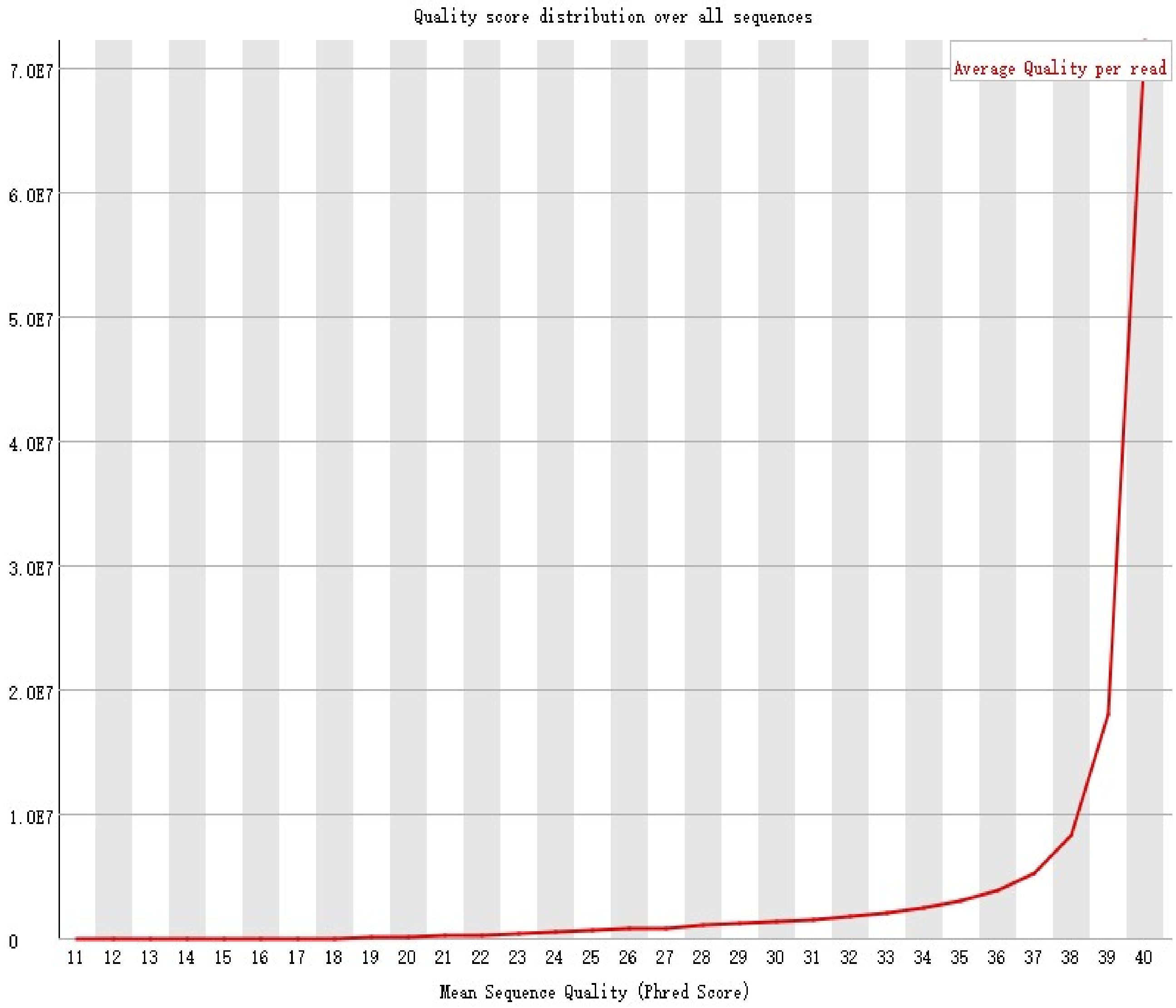

Per base sequence content

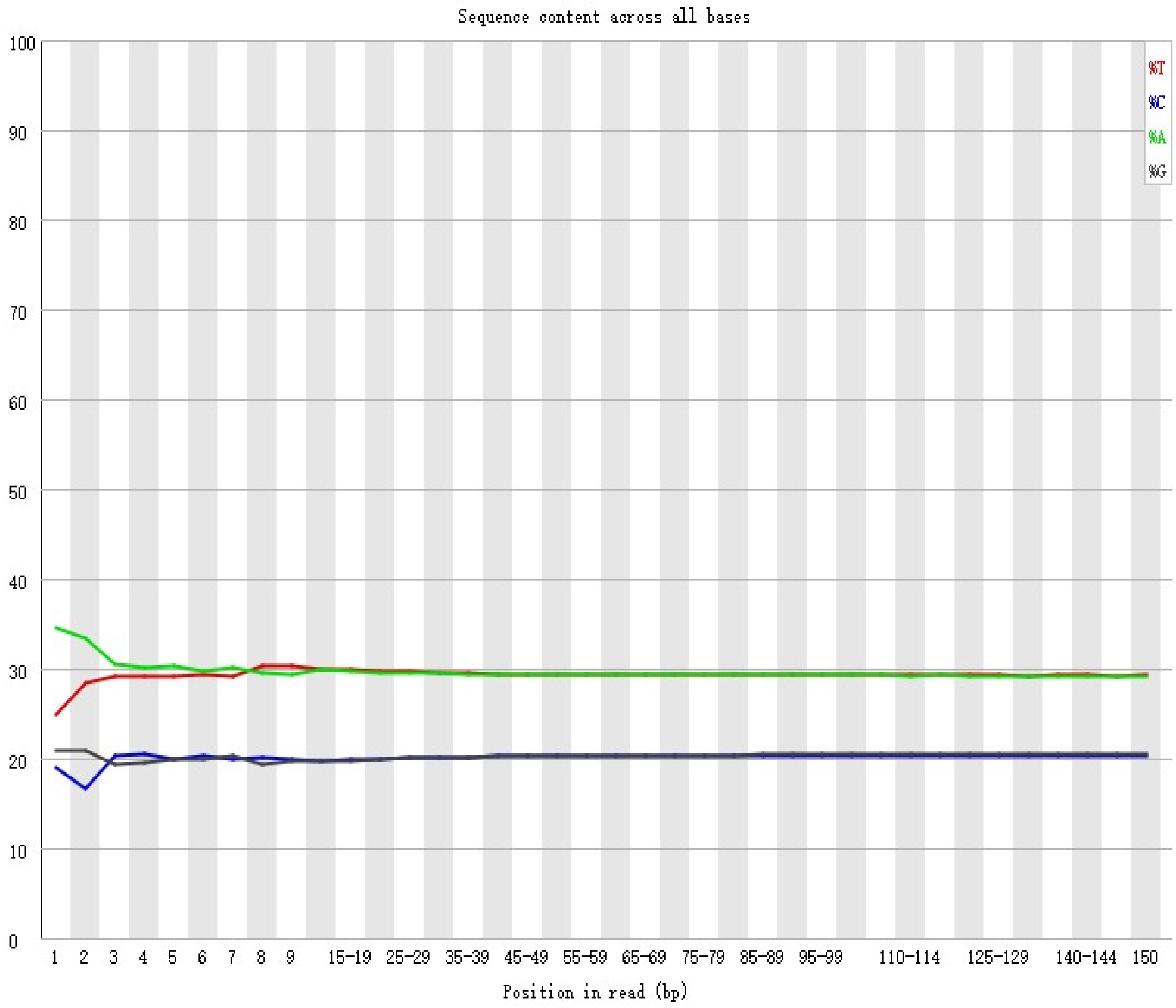

Per sequence GC content

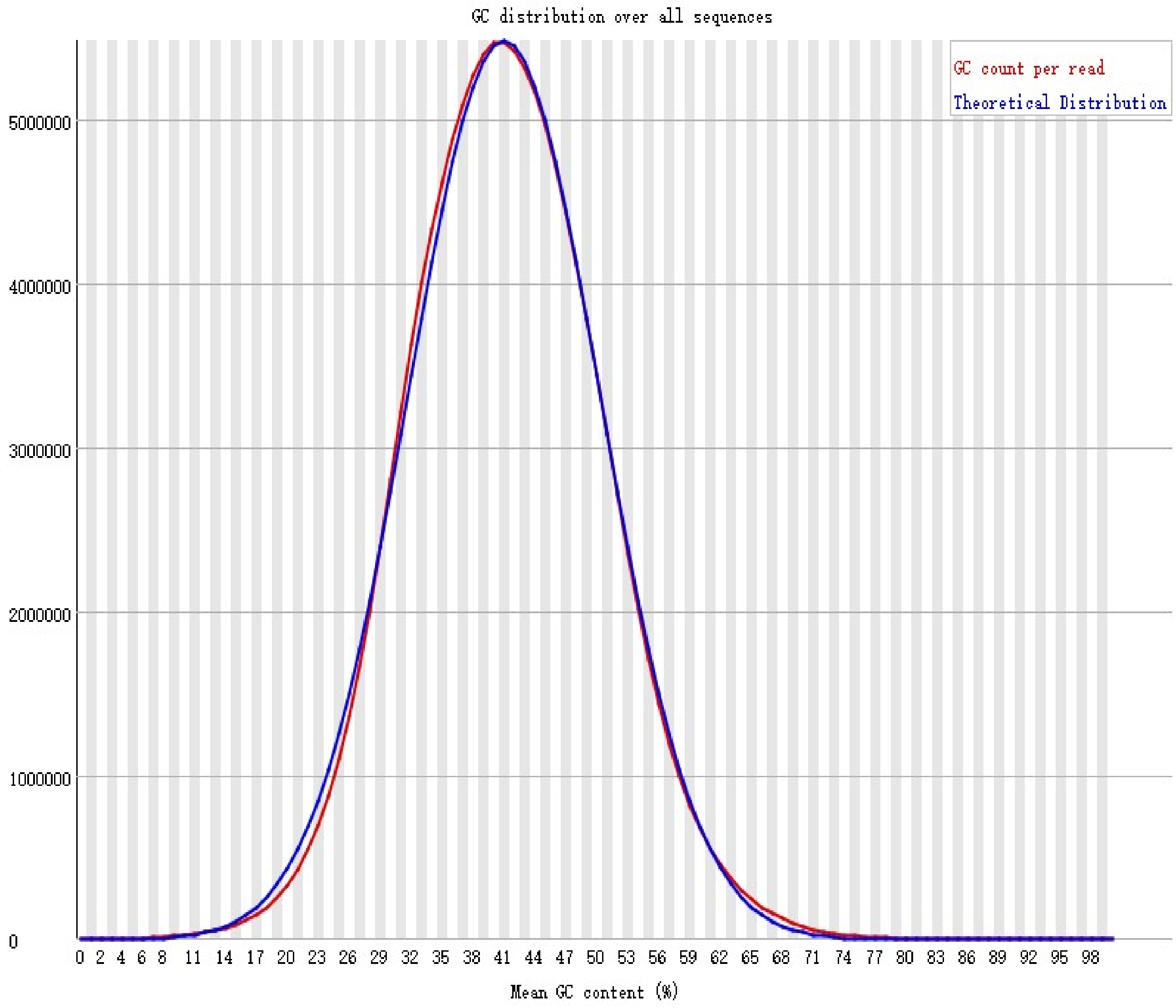

Per base N content

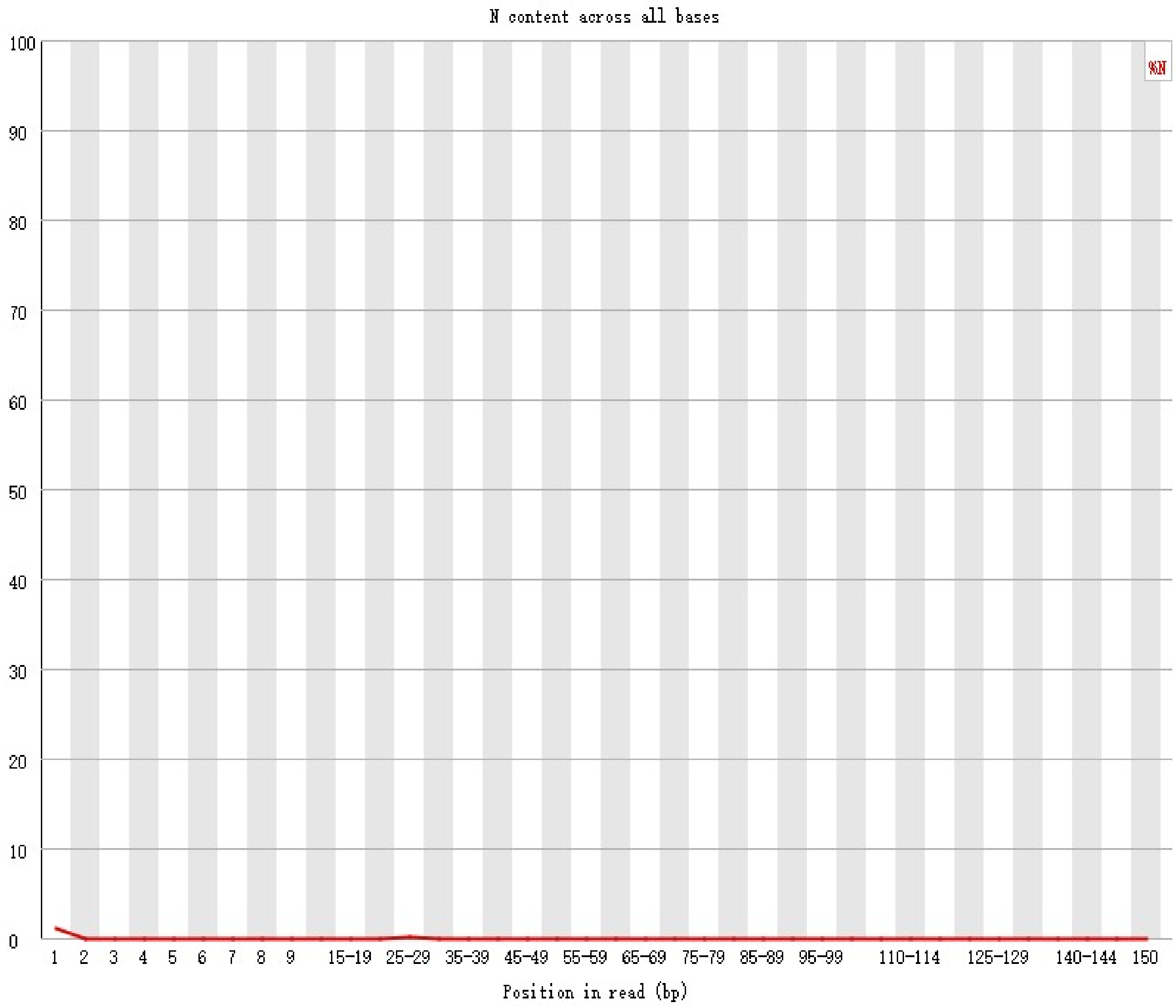

Sequence Length Distribution

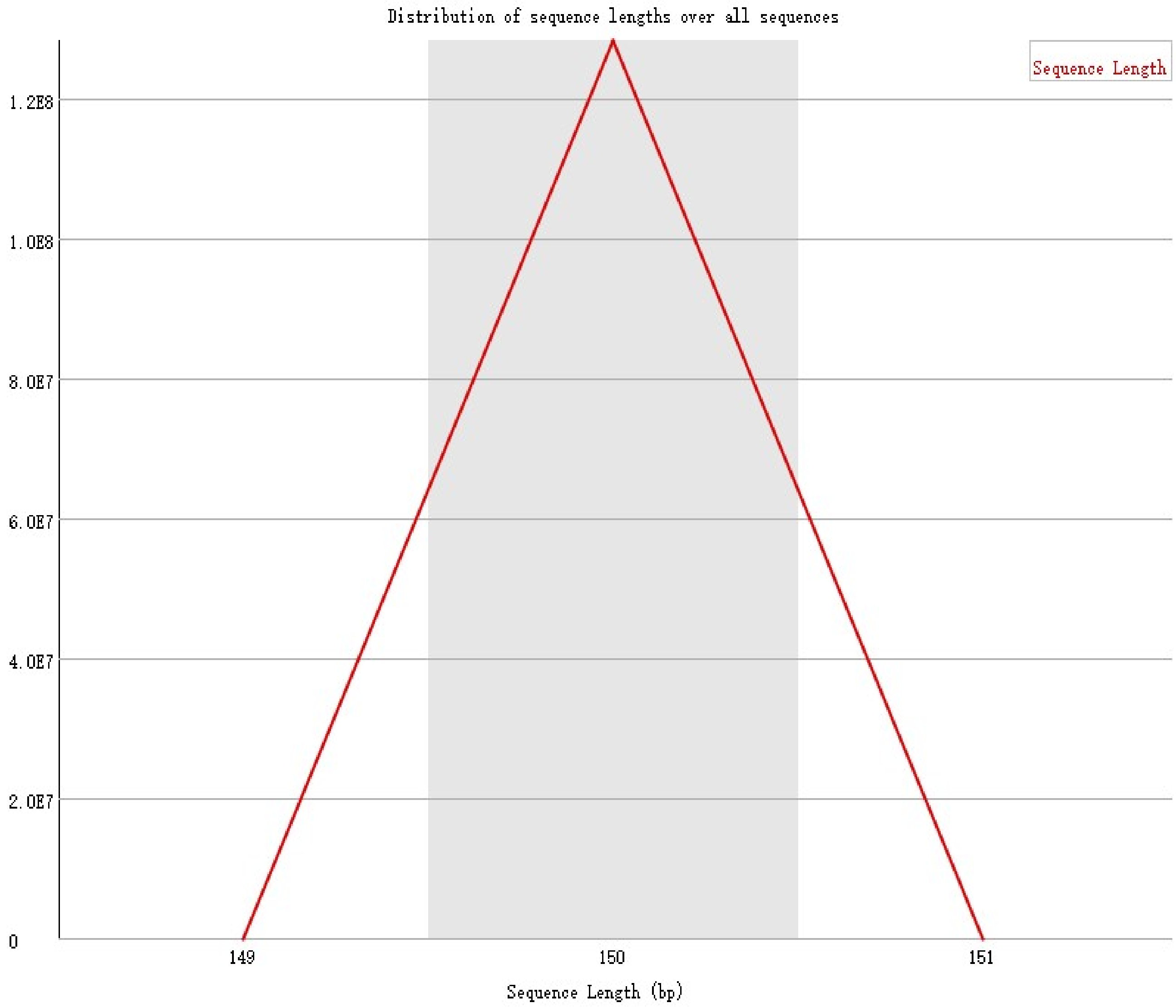

Sequence Duplication Levels

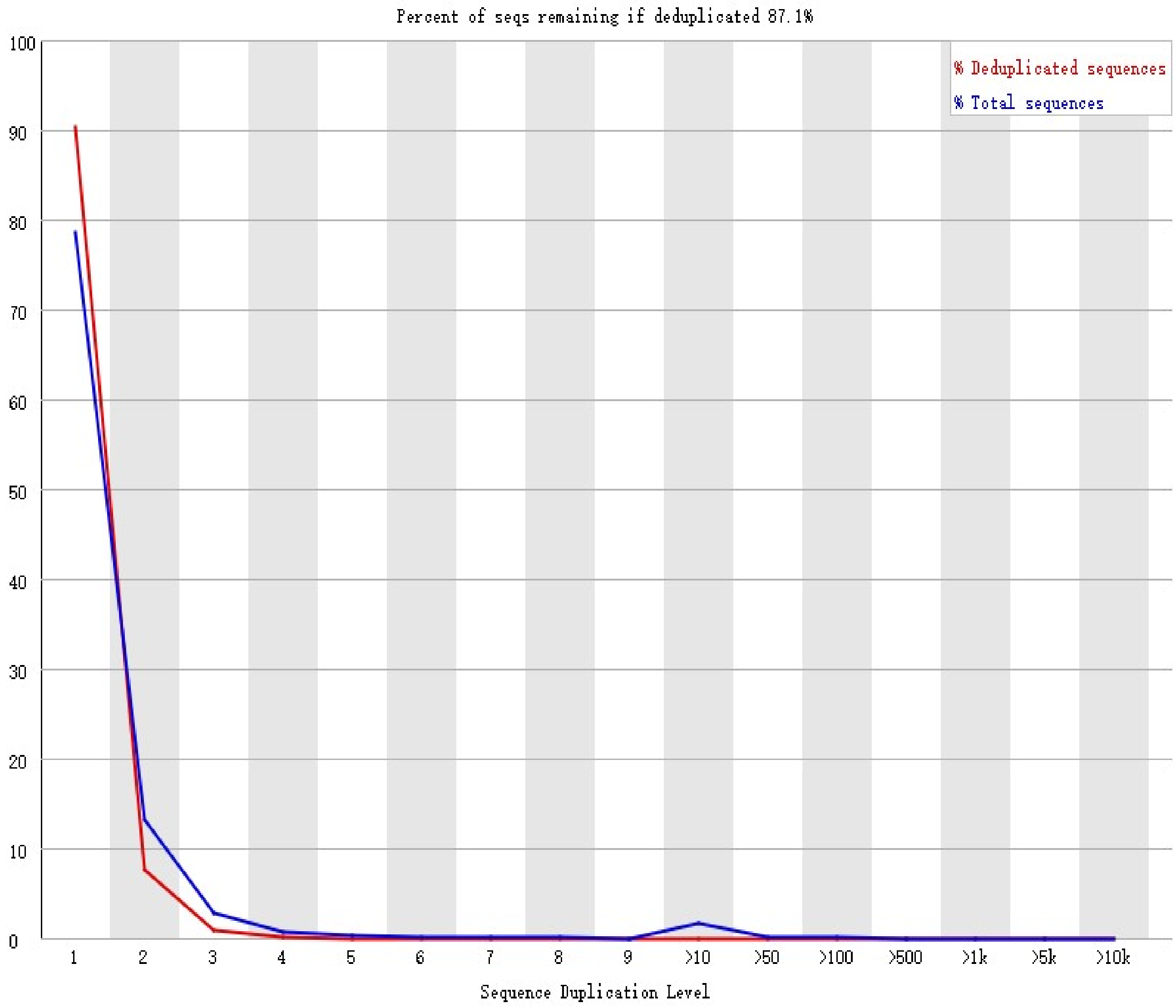

Overrepresented sequences

No overrepresented sequences

Adapter Content

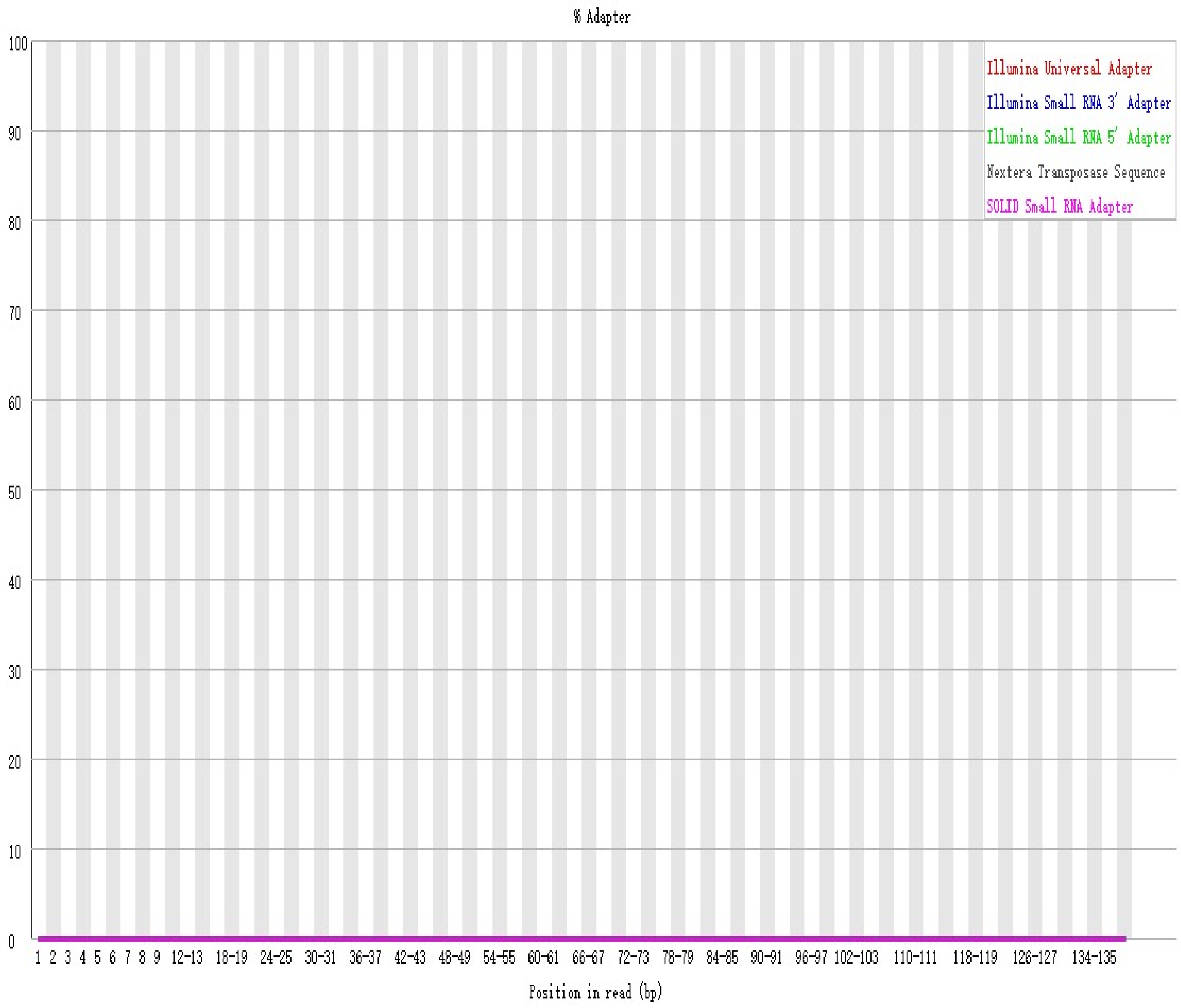

Kmer Content

No overrepresented Kmers

Produced by FastQC (version 0.11.5)

FastQC Report

星期三 8 五月 2019
SK_L4_1_clean.fq.gz

Summary

- Basic Statistics
- Per base sequence quality
- Per tile sequence quality
- Per sequence quality scores
- Per base sequence content
- Per sequence GC content
- Per base N content
- Sequence Length Distribution
- Sequence Duplication Levels
- Overrepresented sequences
- Adapter Content
- Kmer Content

Basic Statistics

Measure	Value
Filename	SK_L4_1_clean.fq.gz
File type	Conventional base calls
Encoding	Sanger / Illumina 1.9
Total Sequences	137026069
Sequences flagged as poor quality	0
Sequence length	150
%GC	41

Per base sequence quality

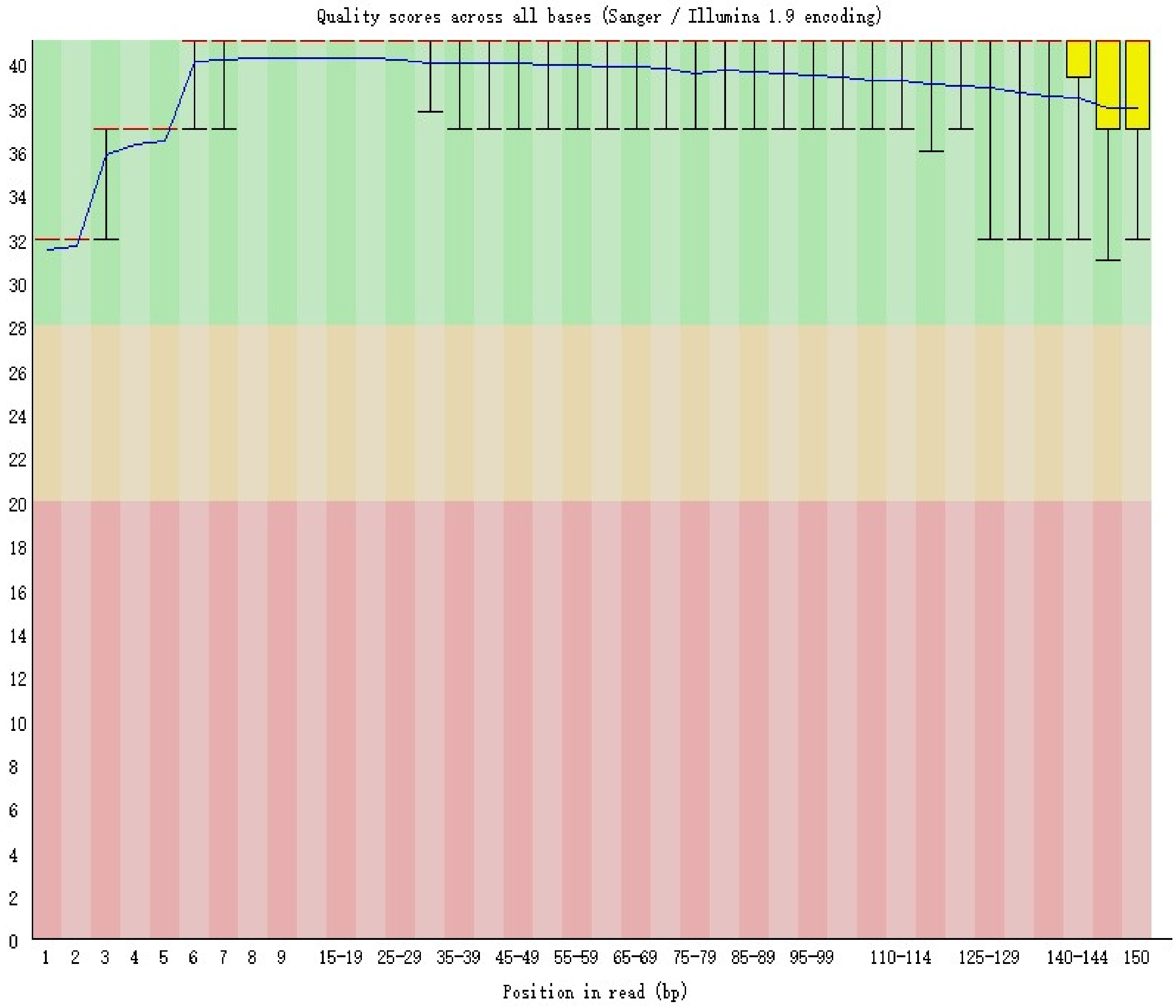

Per tile sequence quality

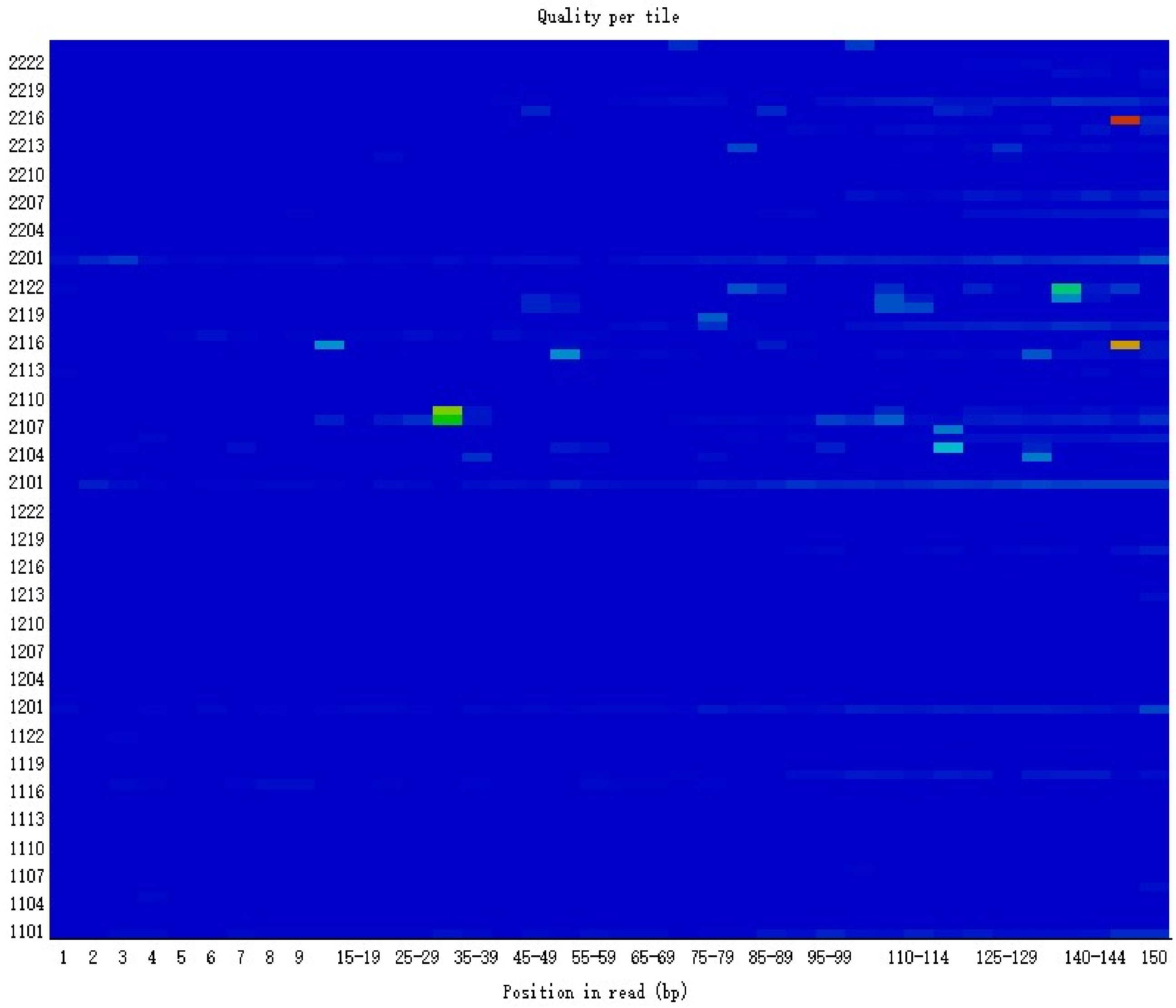

Per sequence quality scores

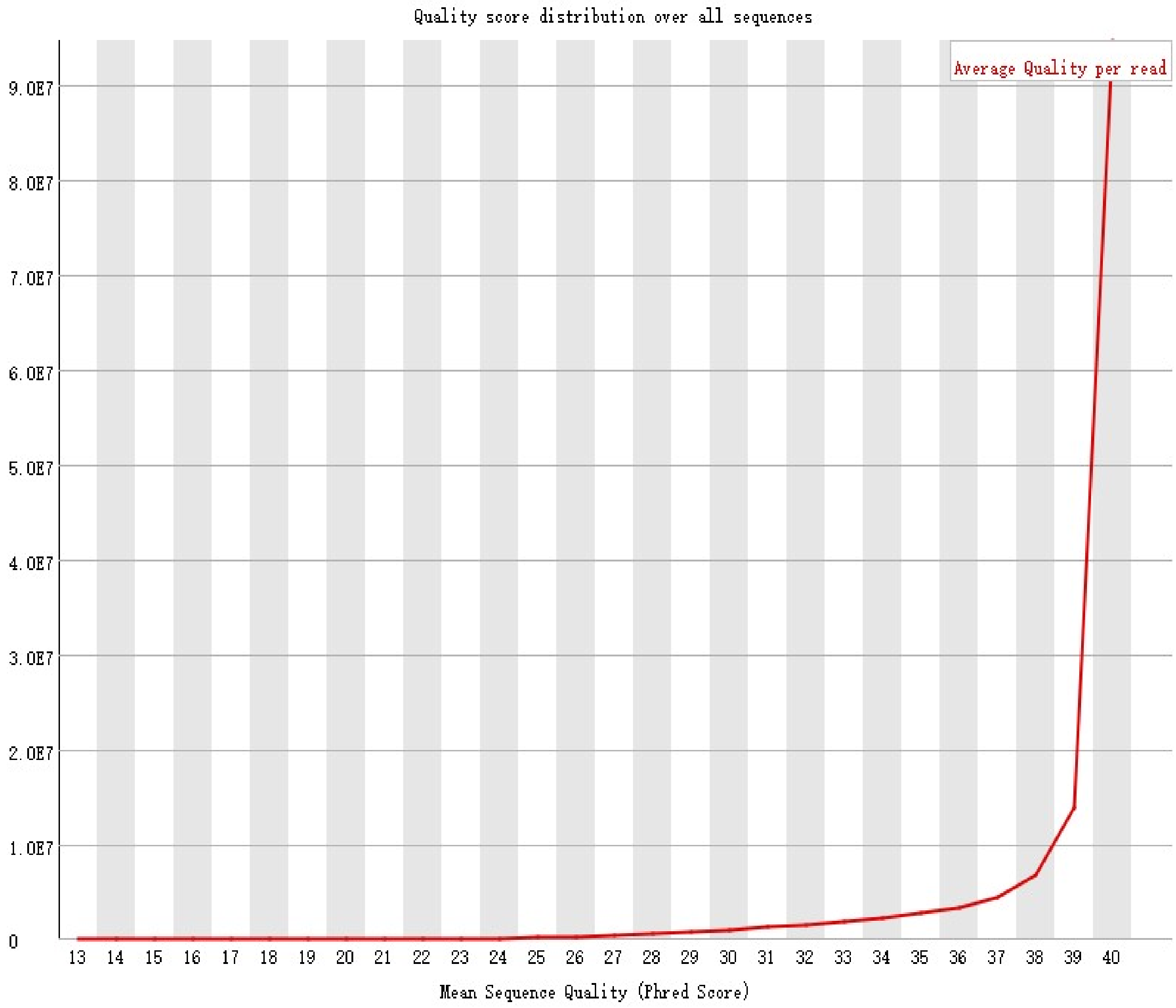

Per base sequence content

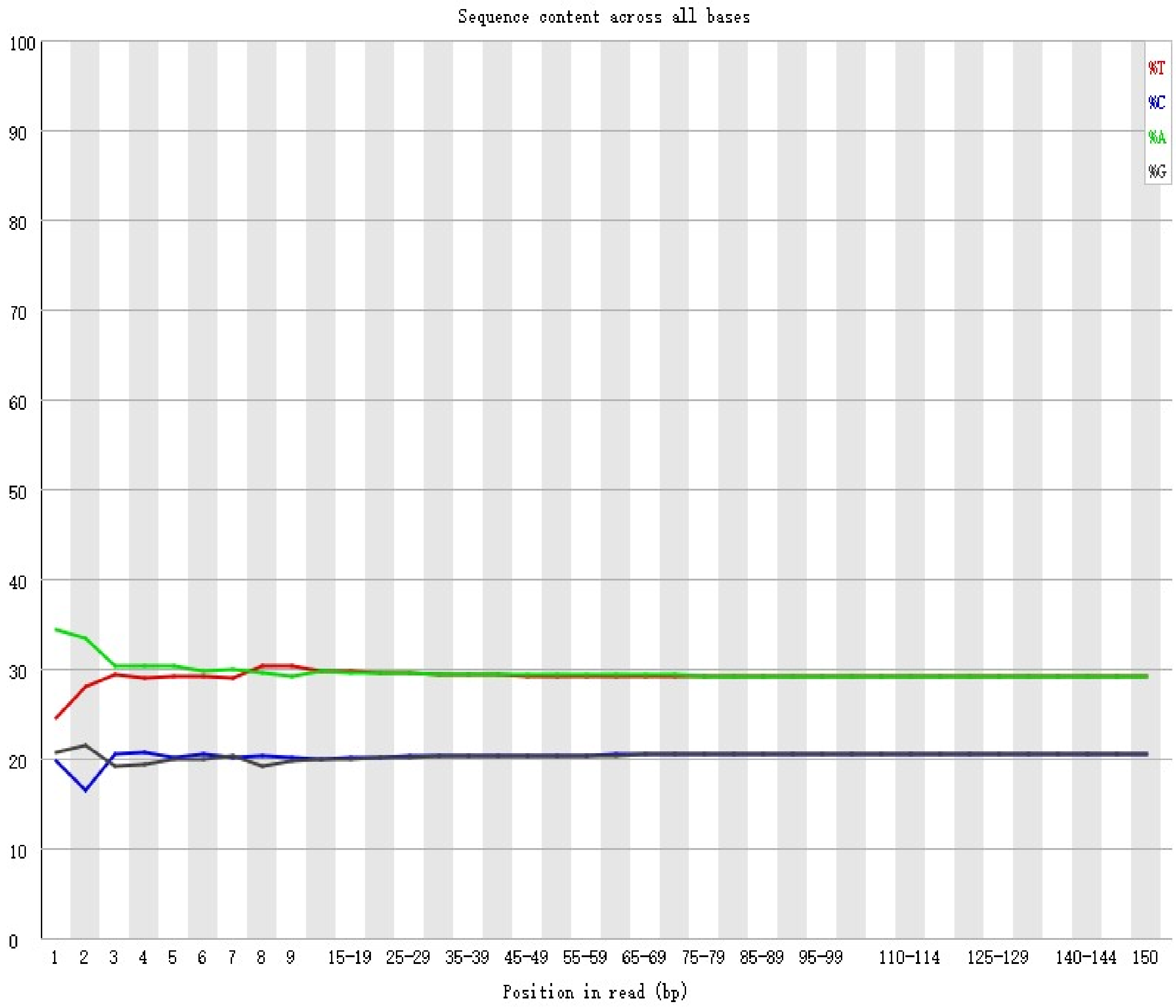

Per sequence GC content

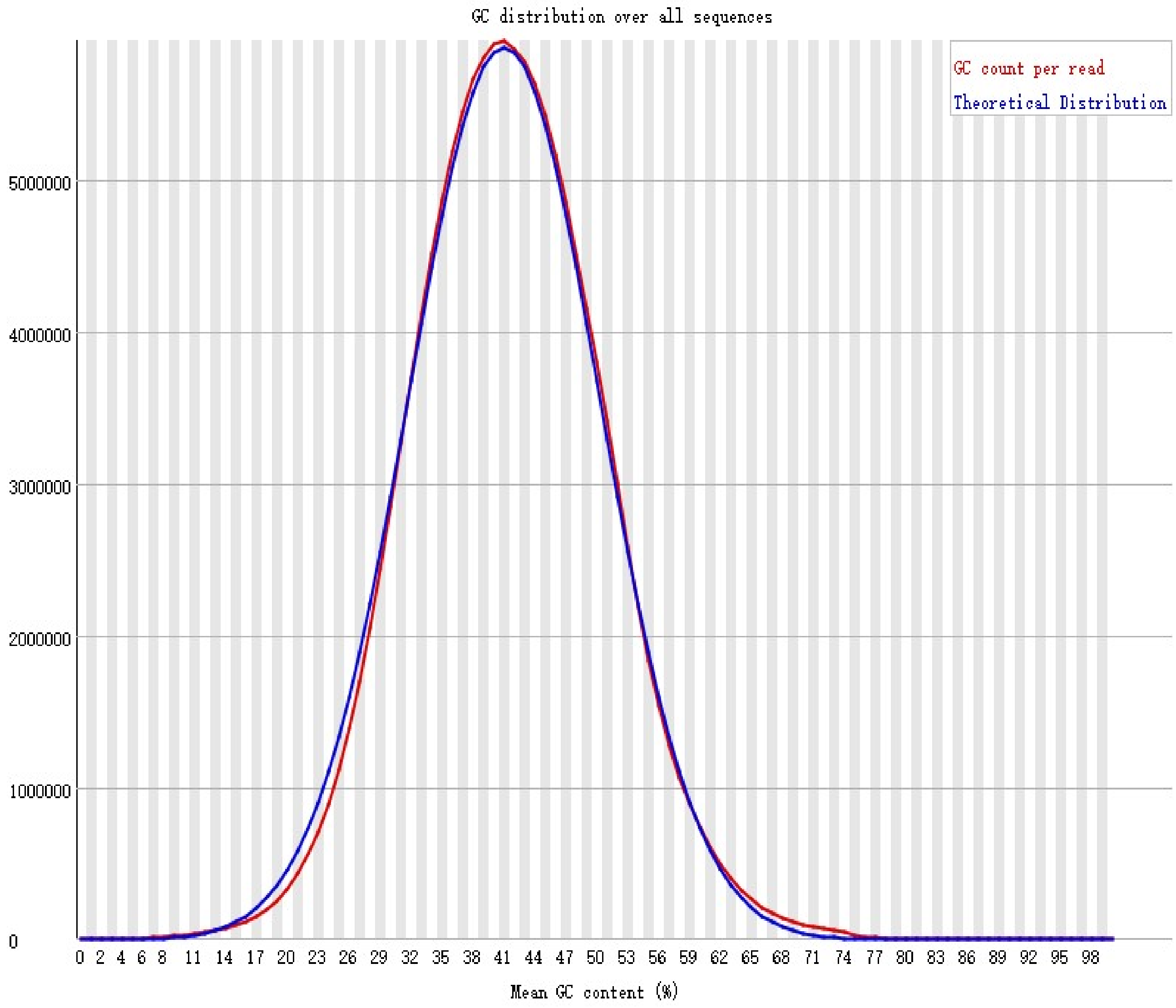

Per base N content

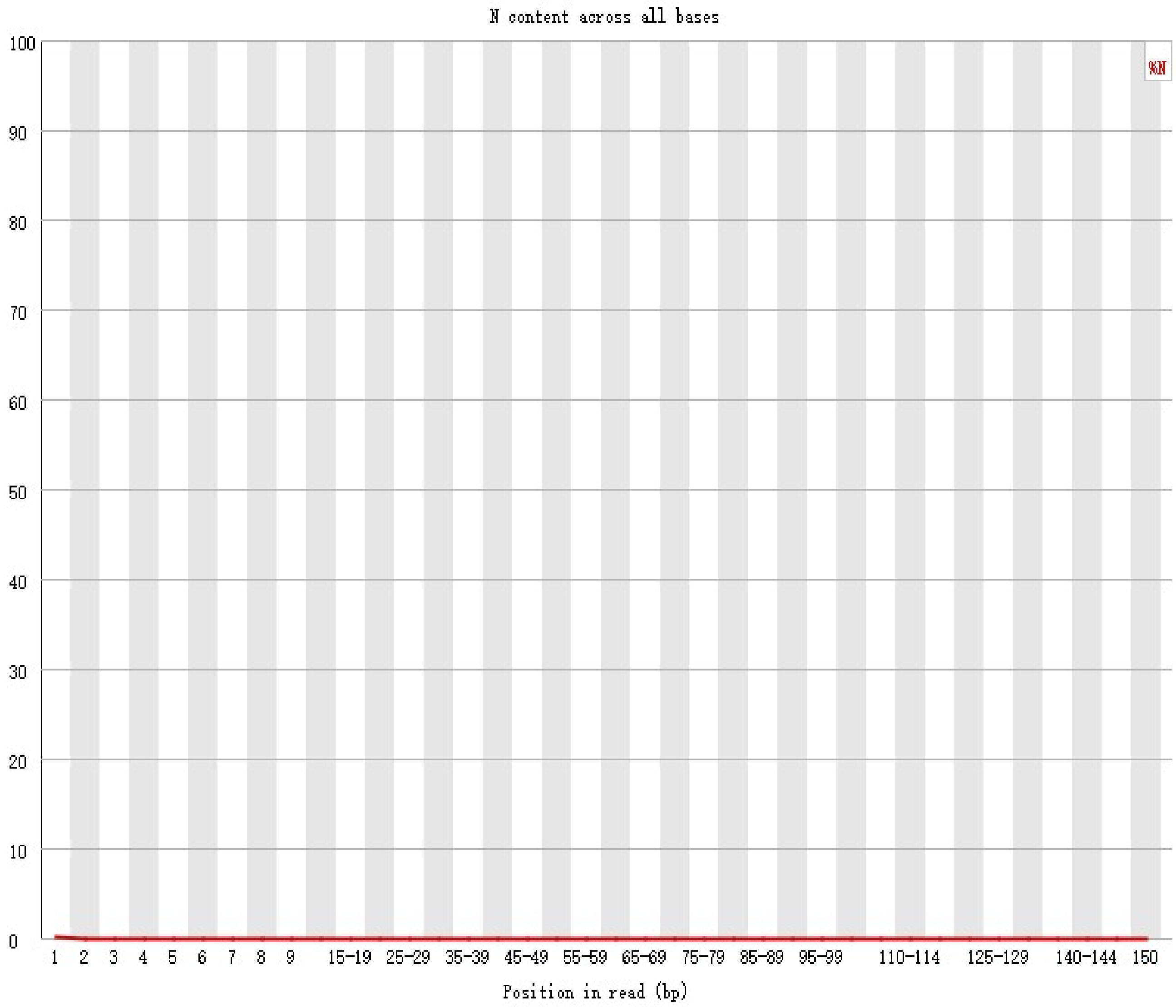

Sequence Length Distribution

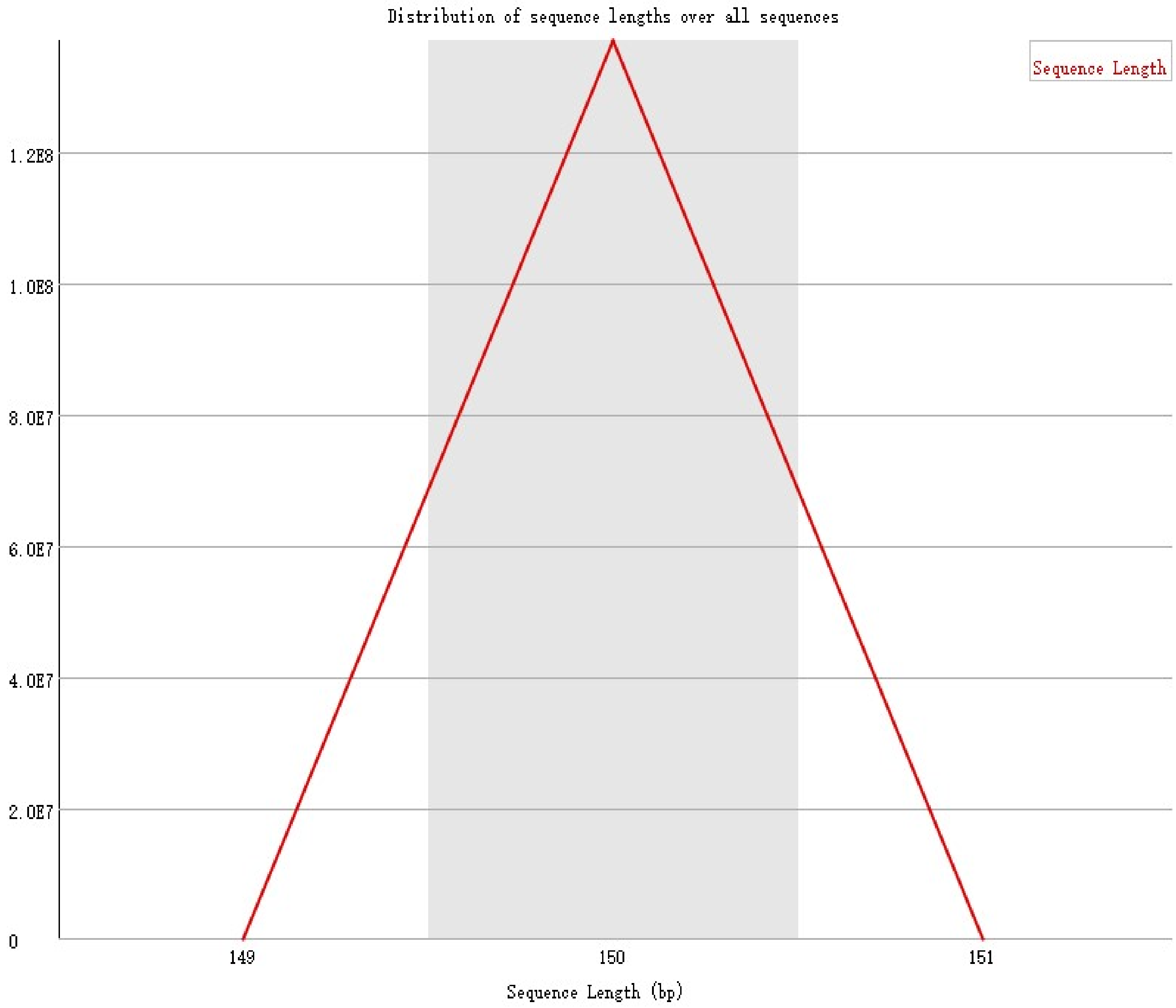

Sequence Duplication Levels

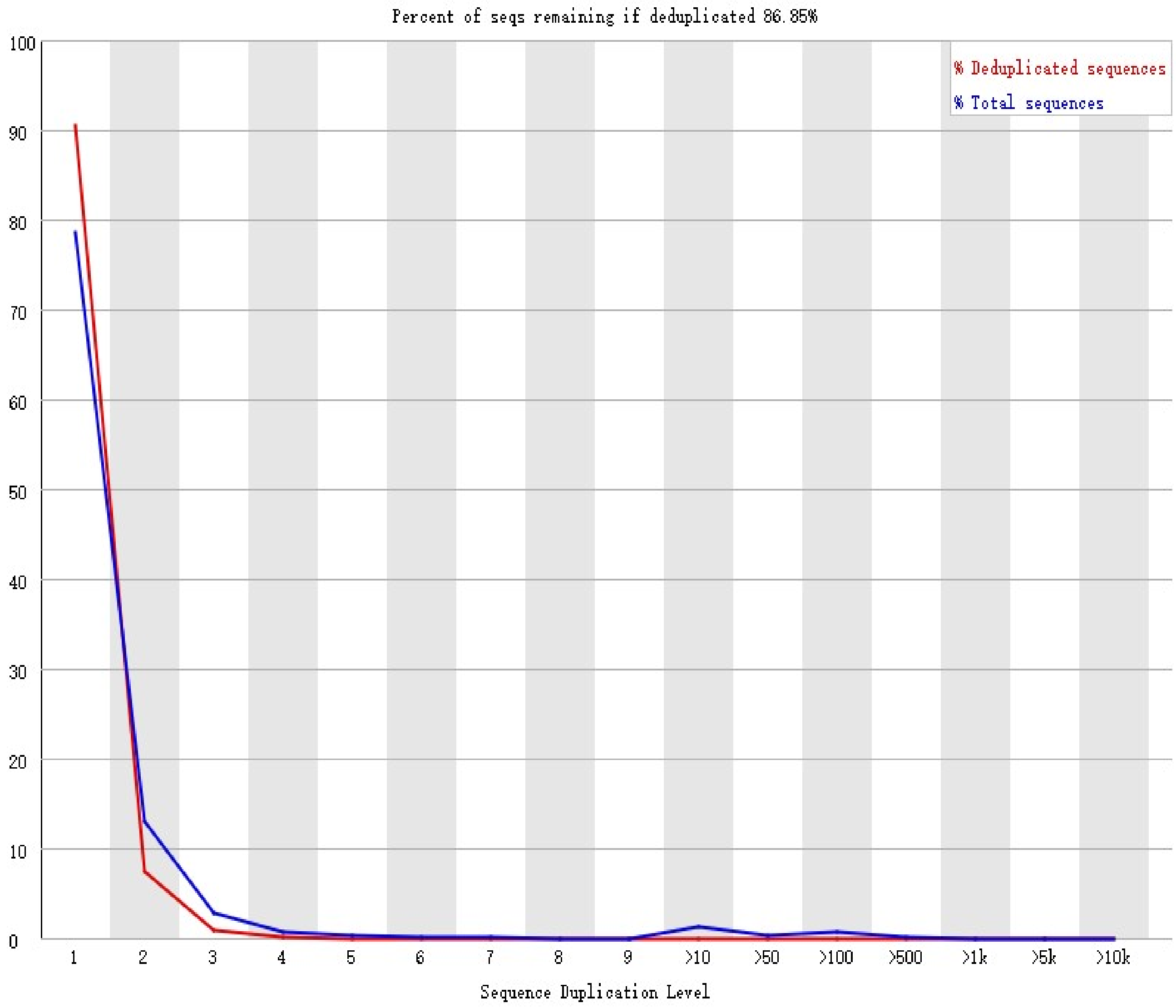

Overrepresented sequences

No overrepresented sequences

Adapter Content

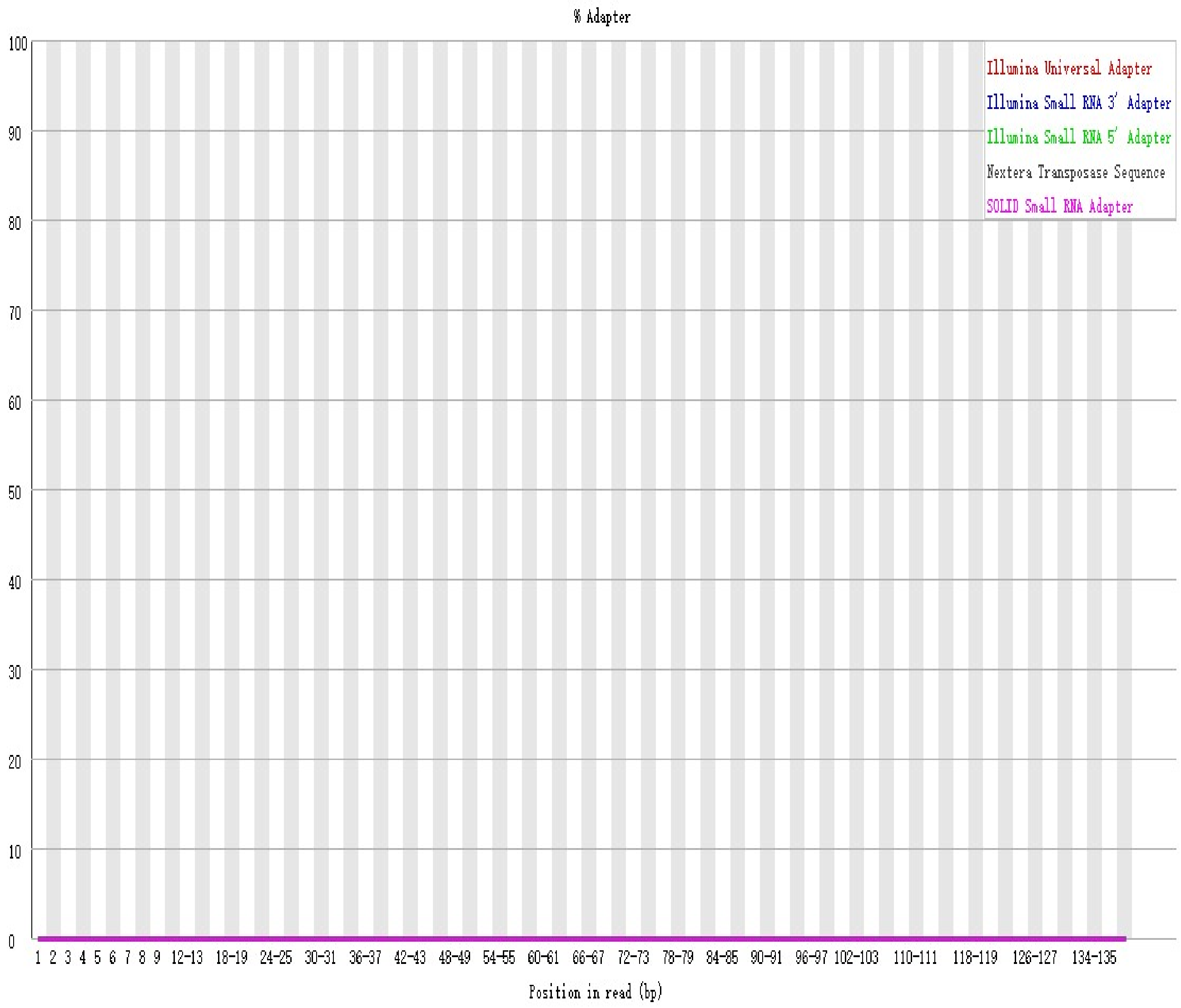

Kmer Content

No overrepresented Kmers

Produced by FastQC (version 0.11.5)

FastQC Report

星期三 8 五月 2019
SK_L4_2_clean.fq.gz

Summary

- Basic Statistics
- Per base sequence quality
- Per tile sequence quality
- Per sequence quality scores
- Per base sequence content
- Per sequence GC content
- Per base N content
- Sequence Length Distribution
- Sequence Duplication Levels
- Overrepresented sequences
- Adapter Content
- Kmer Content

Basic Statistics

Measure	Value
Filename	SK_L4_2_clean.fq.gz
File type	Conventional base calls
Encoding	Sanger / Illumina 1.9
Total Sequences	137026069
Sequences flagged as poor quality	0
Sequence length	150
%GC	41

Per base sequence quality

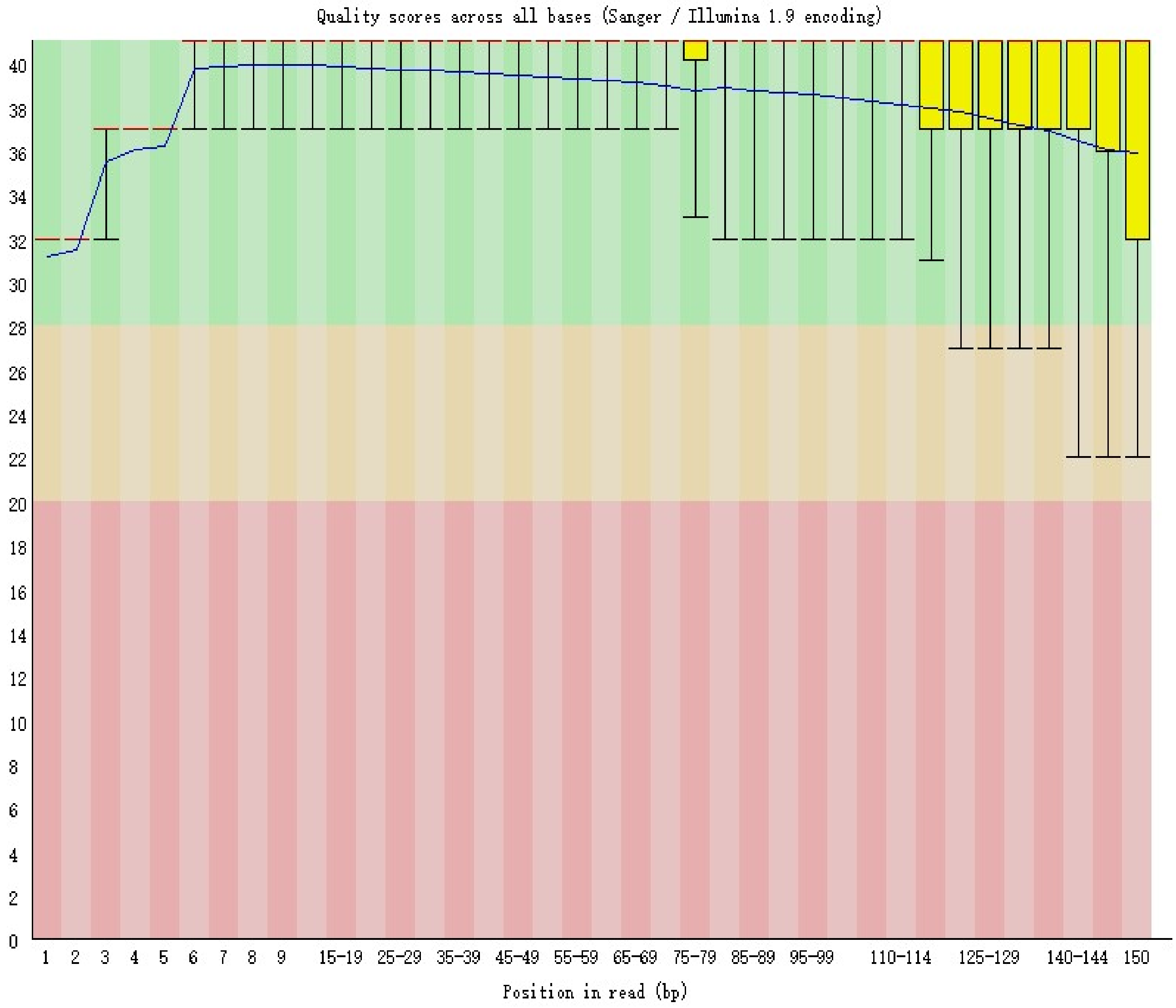

Per tile sequence quality

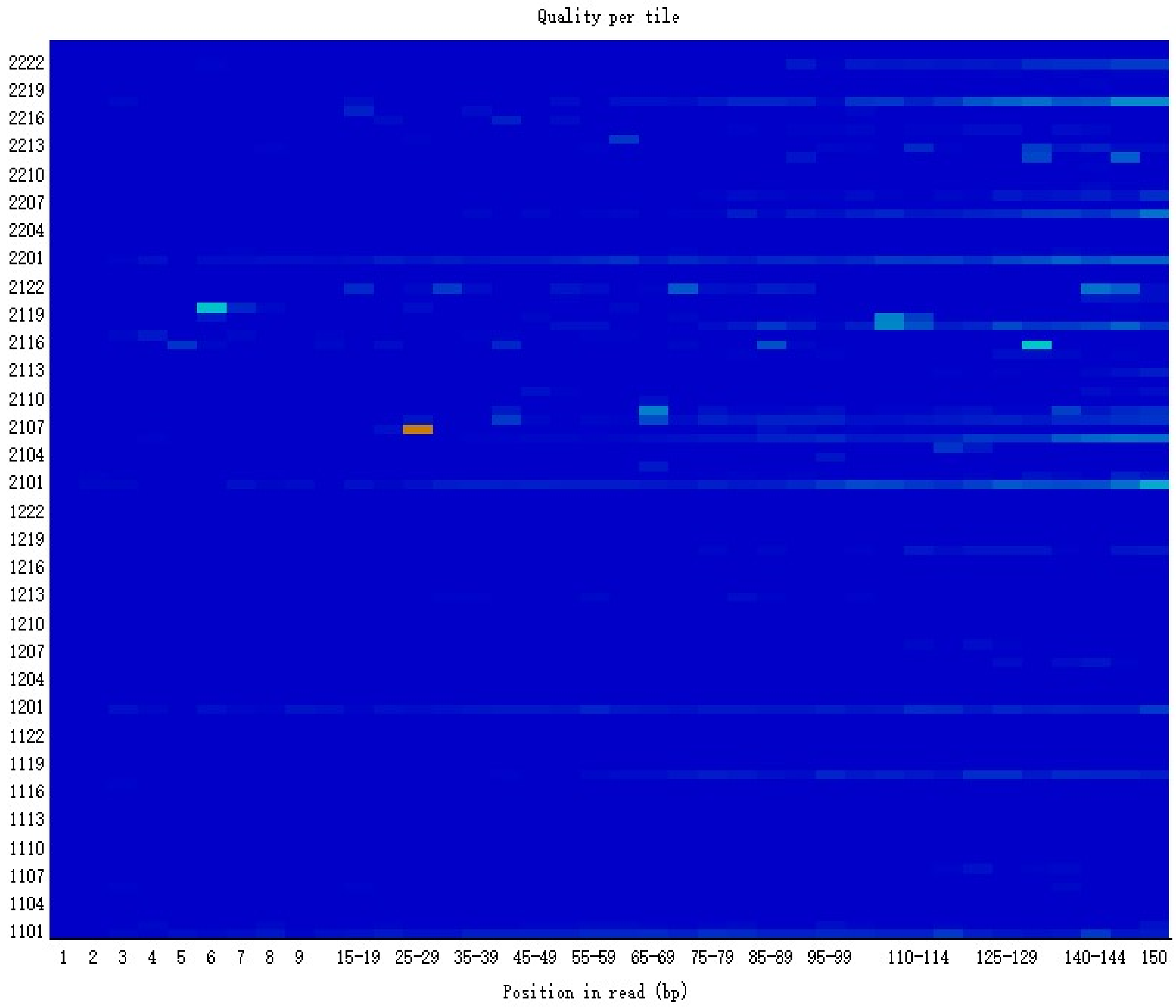

Per sequence quality scores

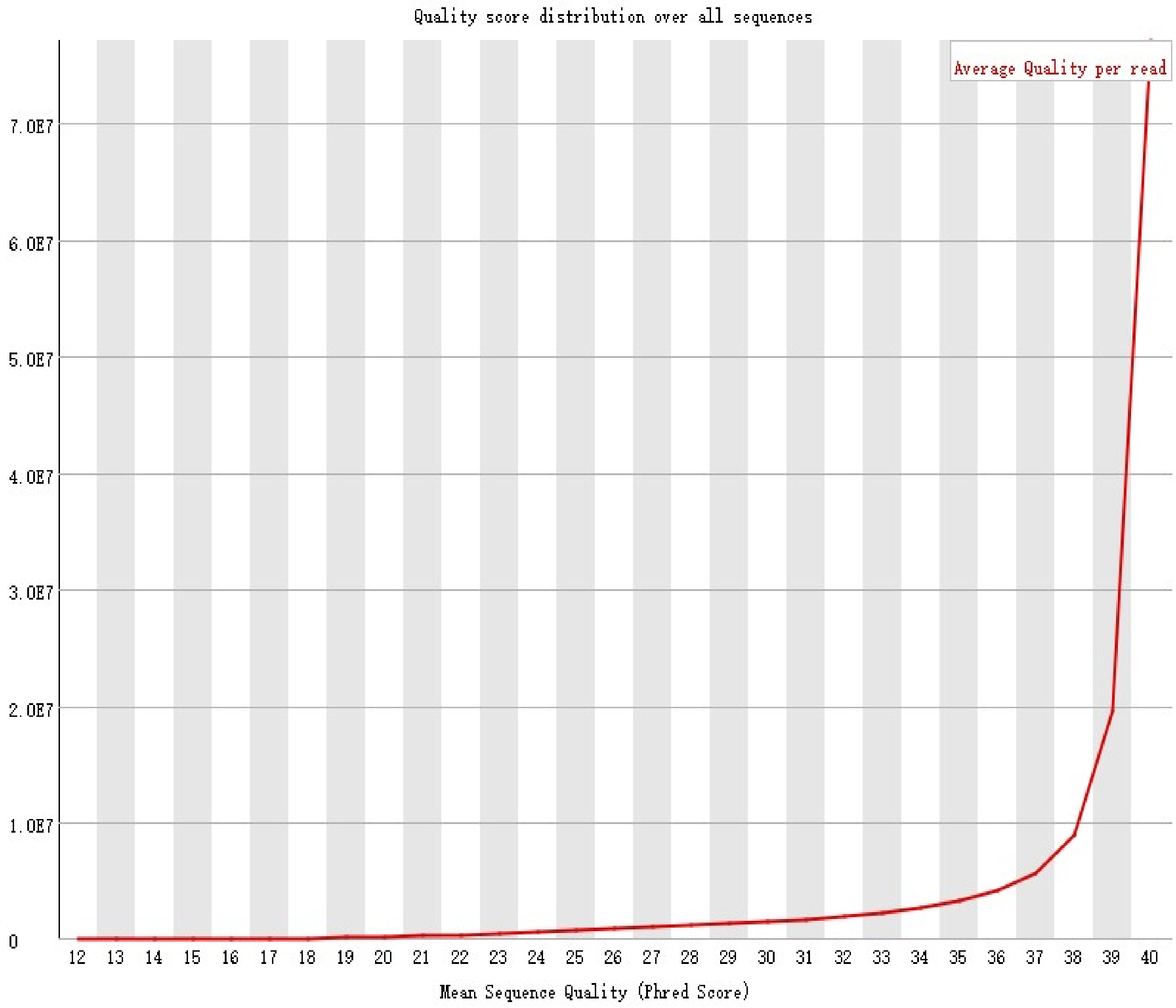

Per base sequence content

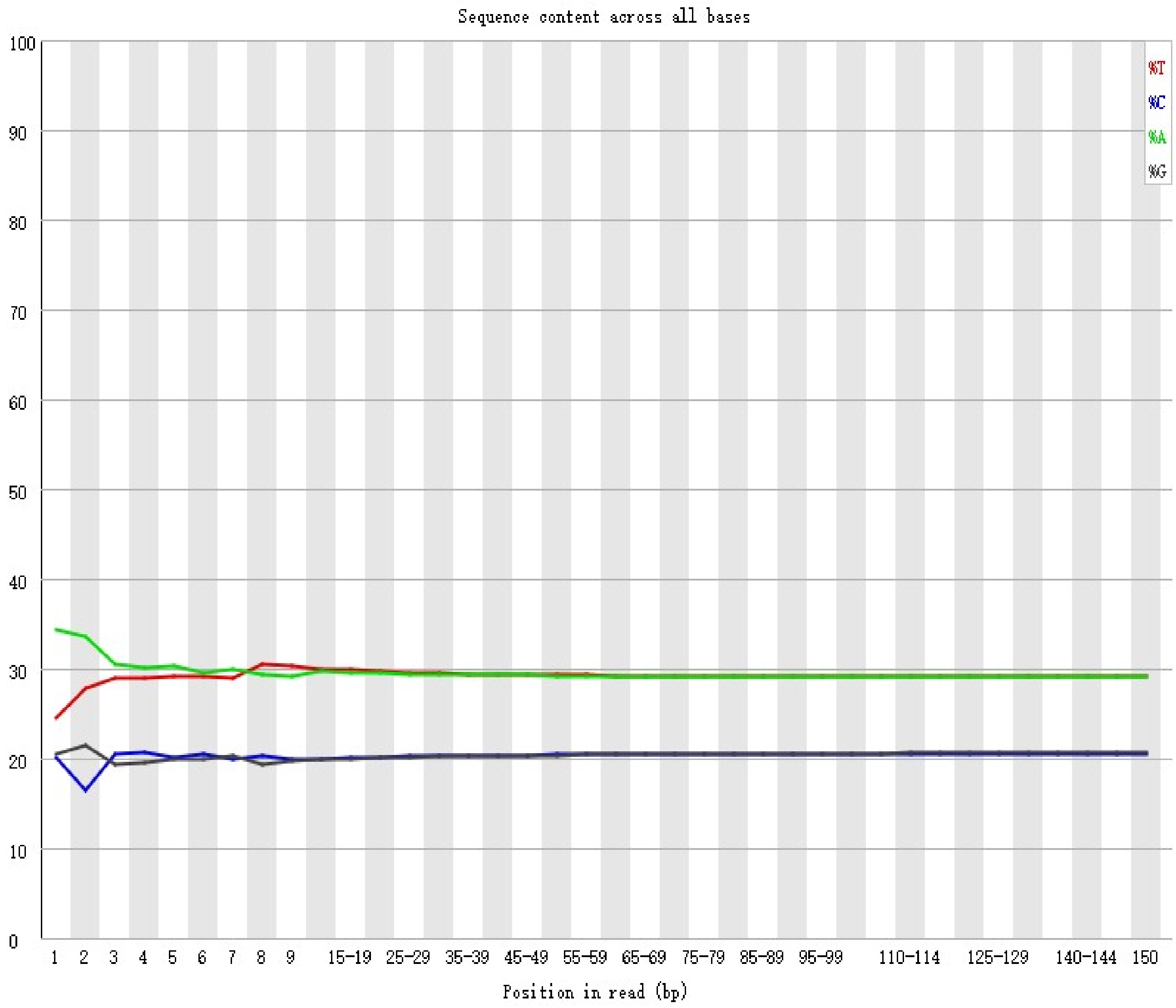

Per sequence GC content

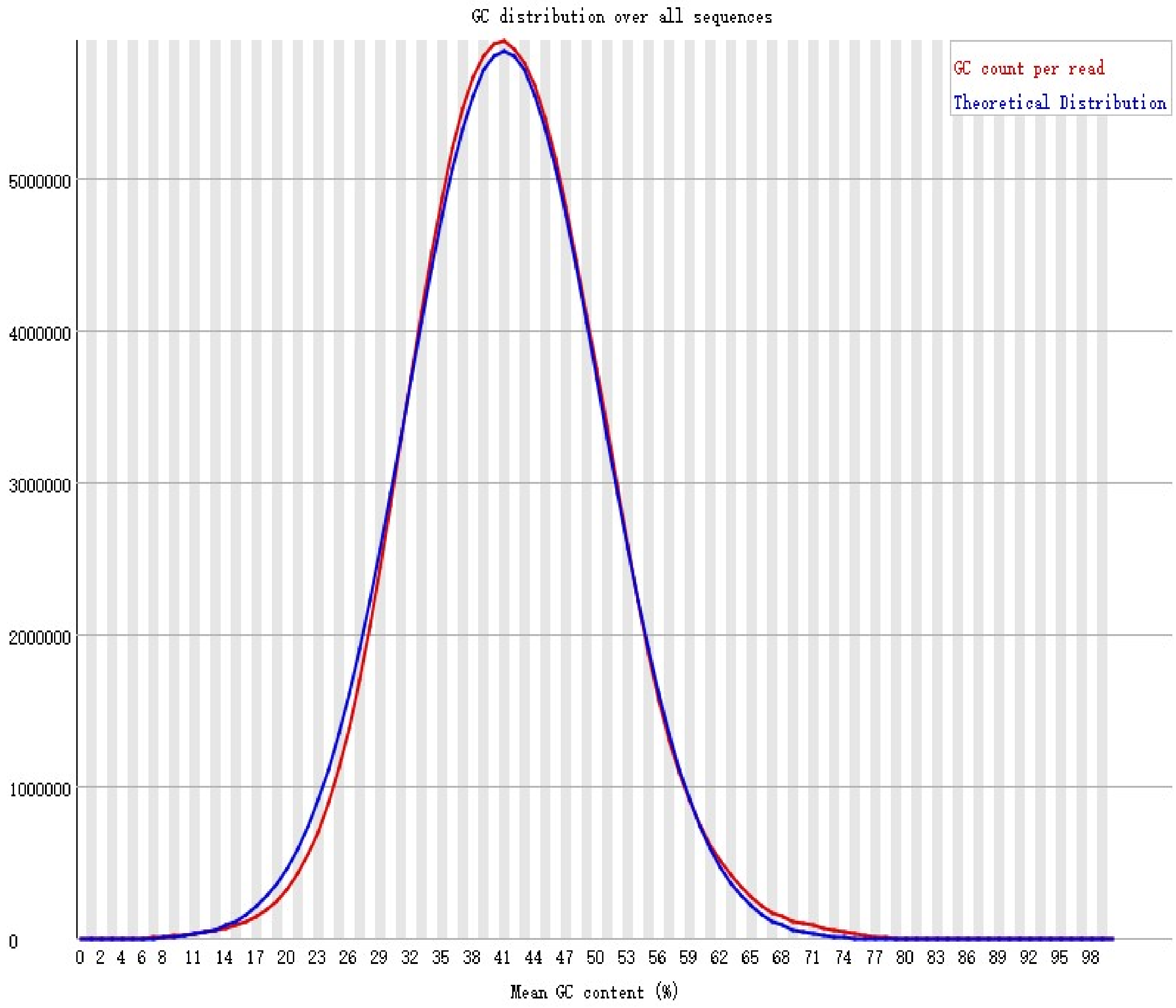

Per base N content

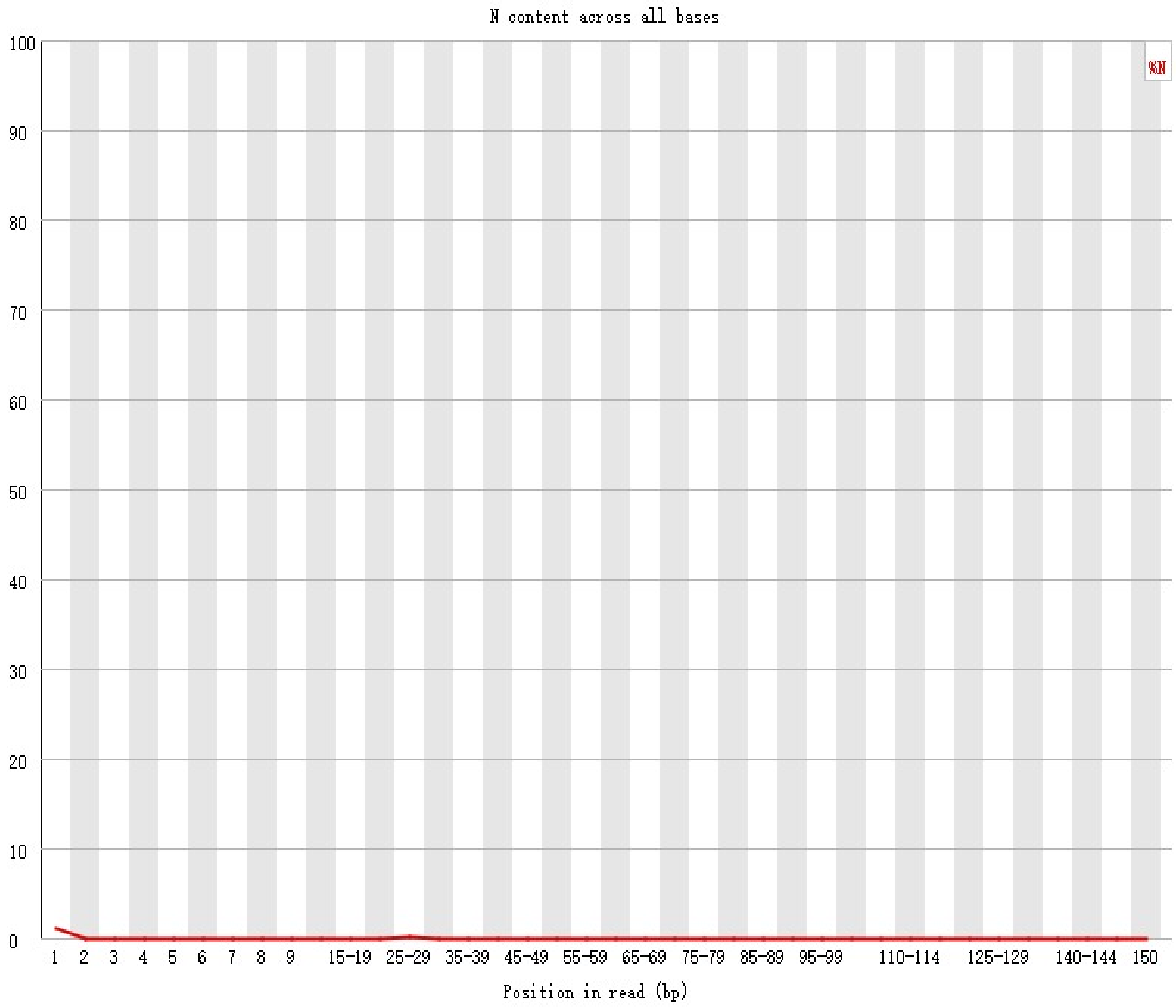

Sequence Length Distribution

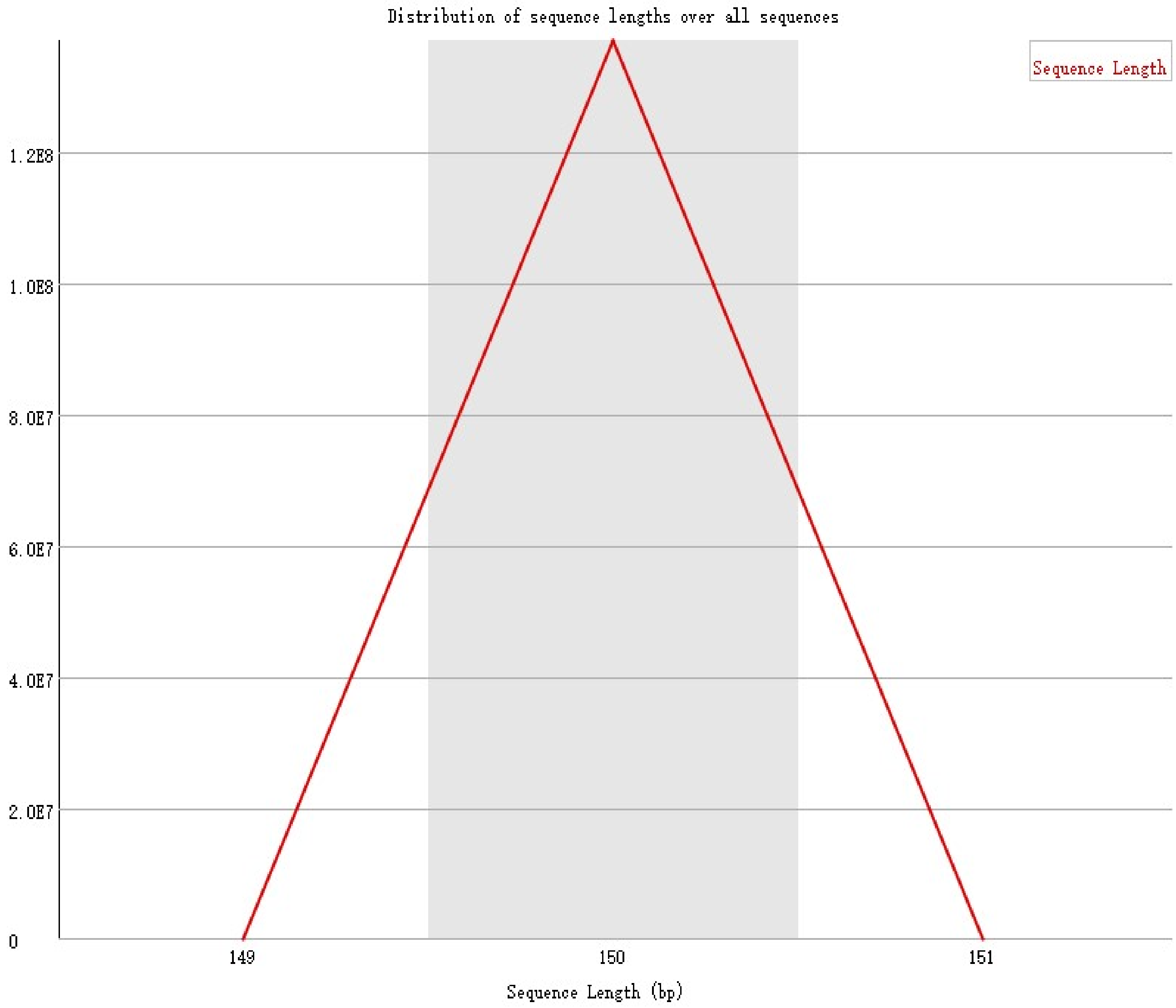

Sequence Duplication Levels

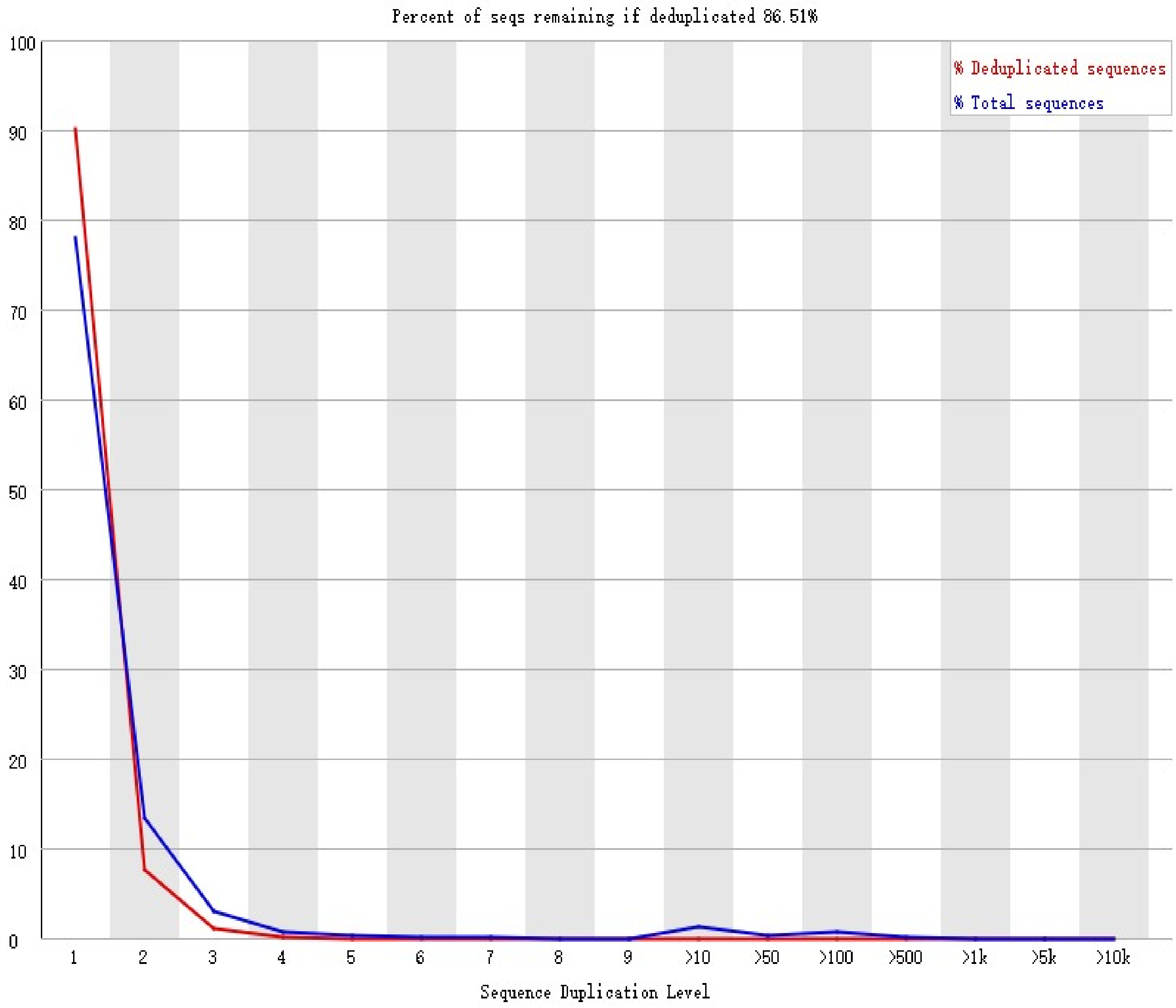

Overrepresented sequences

No overrepresented sequences

Adapter Content

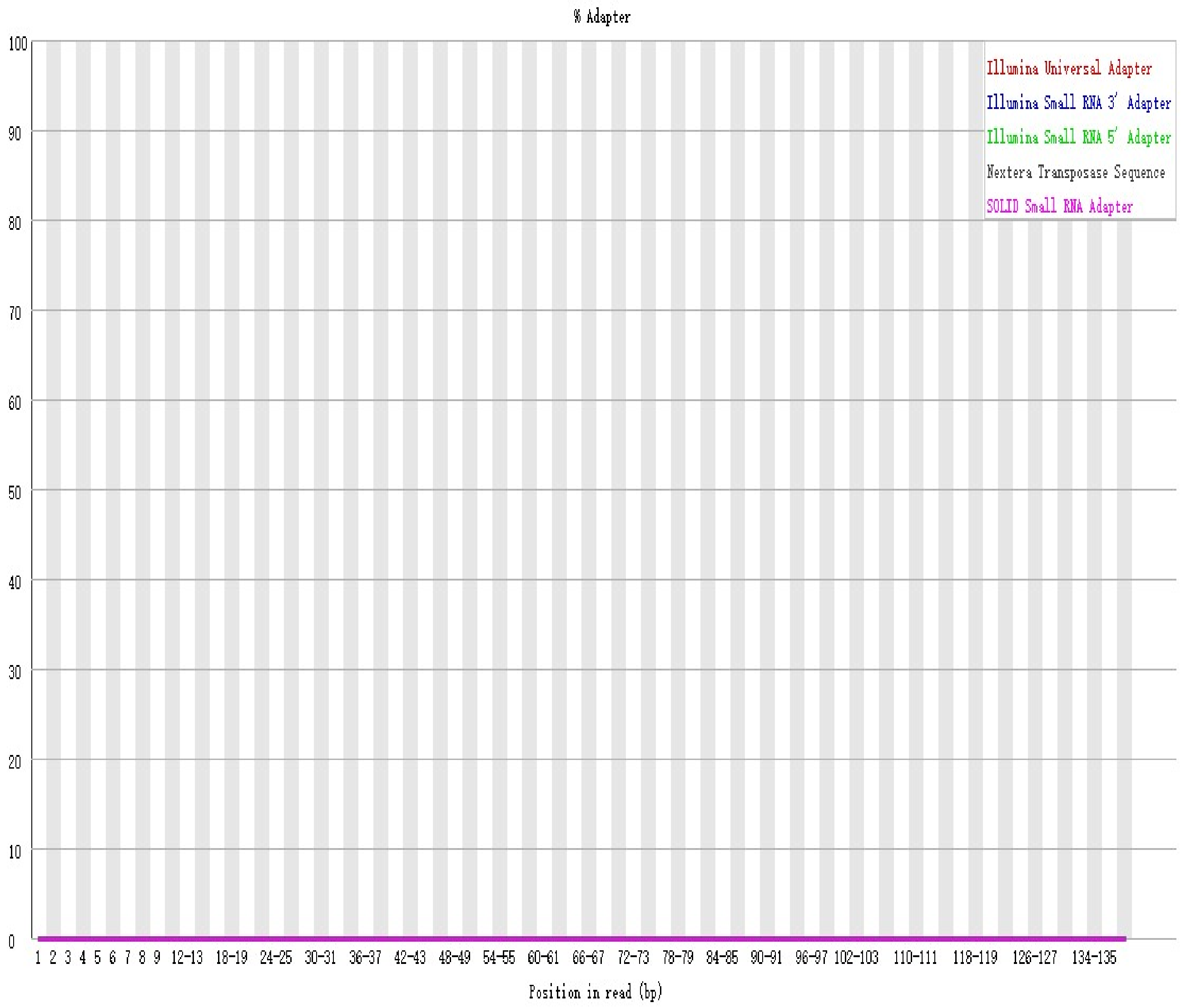

Kmer Content

No overrepresented Kmers

Produced by FastQC (version 0.11.5)

FastQC Report

星期三 8 五月 2019
SN_1_clean.rd.fq.gz

Summary

- Basic Statistics
- Per base sequence quality
- Per tile sequence quality
- Per sequence quality scores
- Per base sequence content
- Per sequence GC content
- Per base N content
- Sequence Length Distribution
- Sequence Duplication Levels
- Overrepresented sequences
- Adapter Content
- Kmer Content

Basic Statistics

Measure	Value
Filename	SN_1_clean.rd.fq.gz
File type	Conventional base calls
Encoding	Sanger / Illumina 1.9
Total Sequences	108737330
Sequences flagged as poor quality	0
Sequence length	150
%GC	41

Per base sequence quality

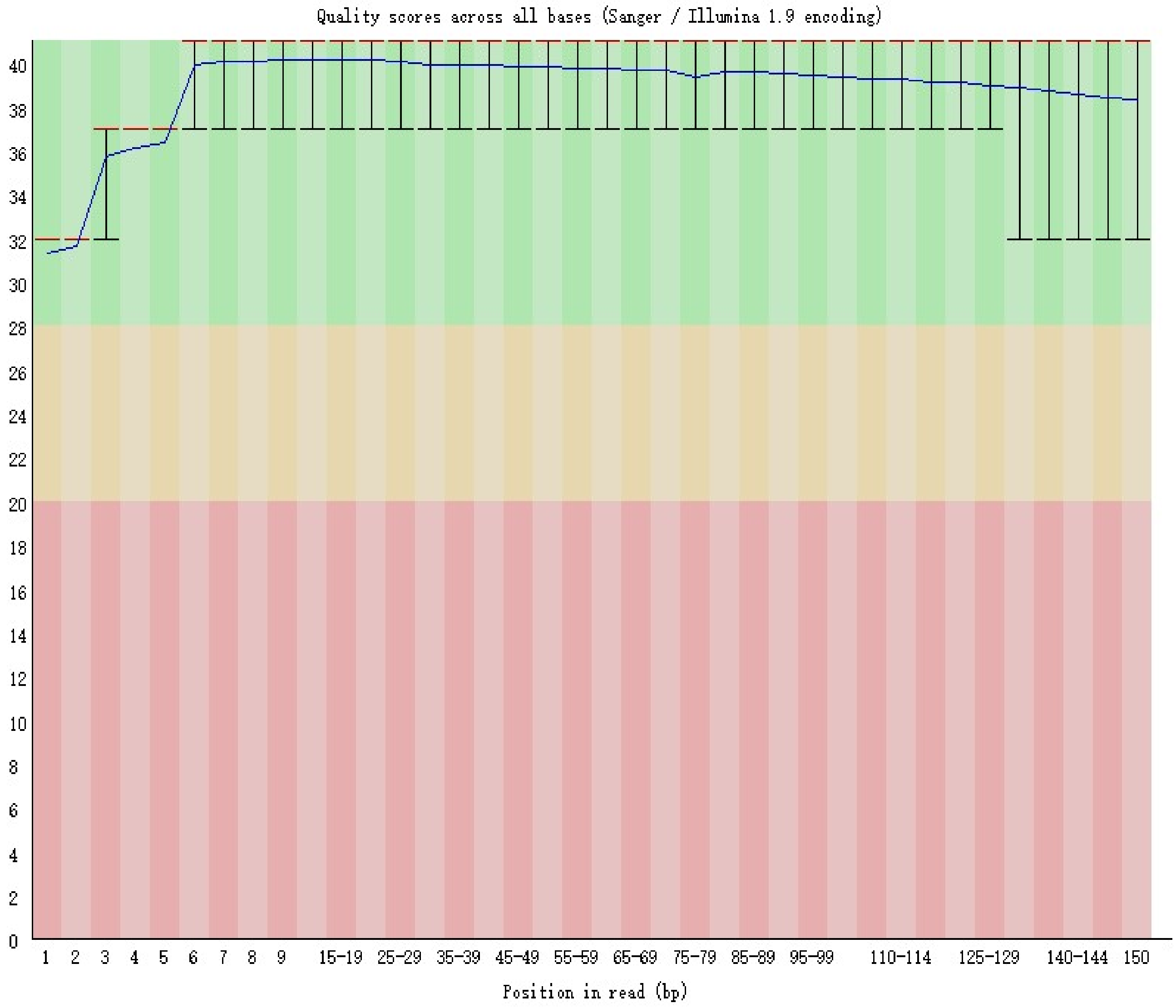

Per tile sequence quality

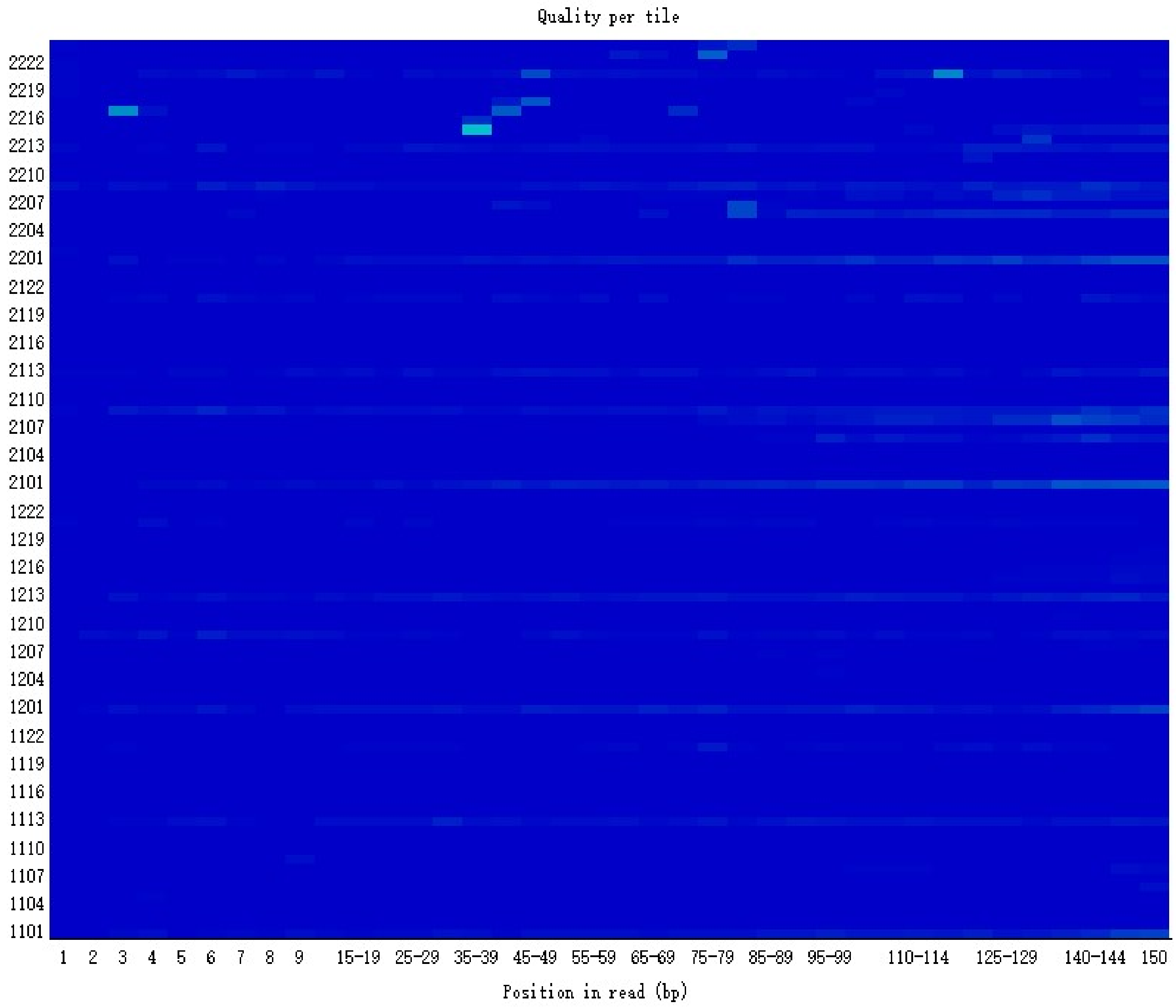

Per sequence quality scores

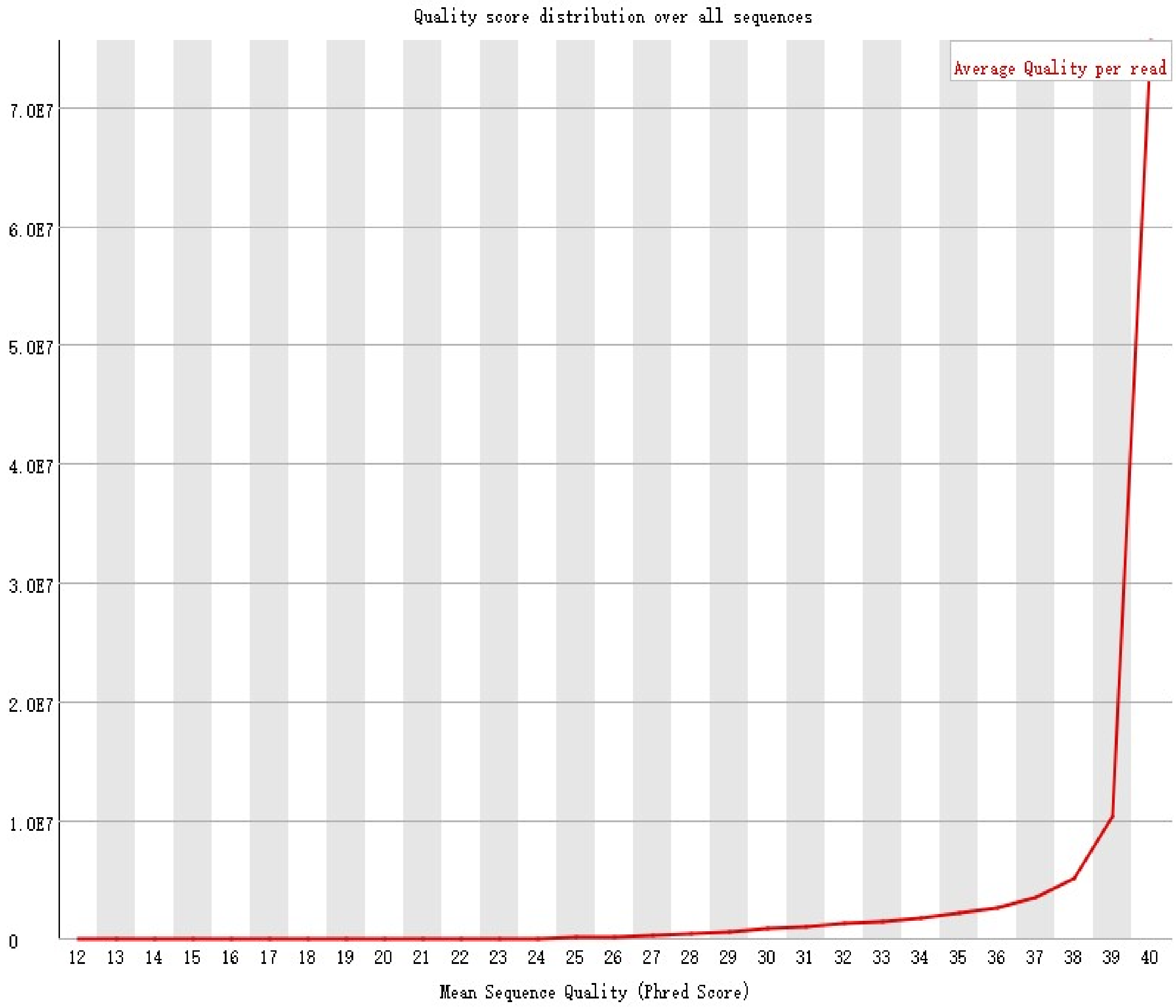

Per base sequence content

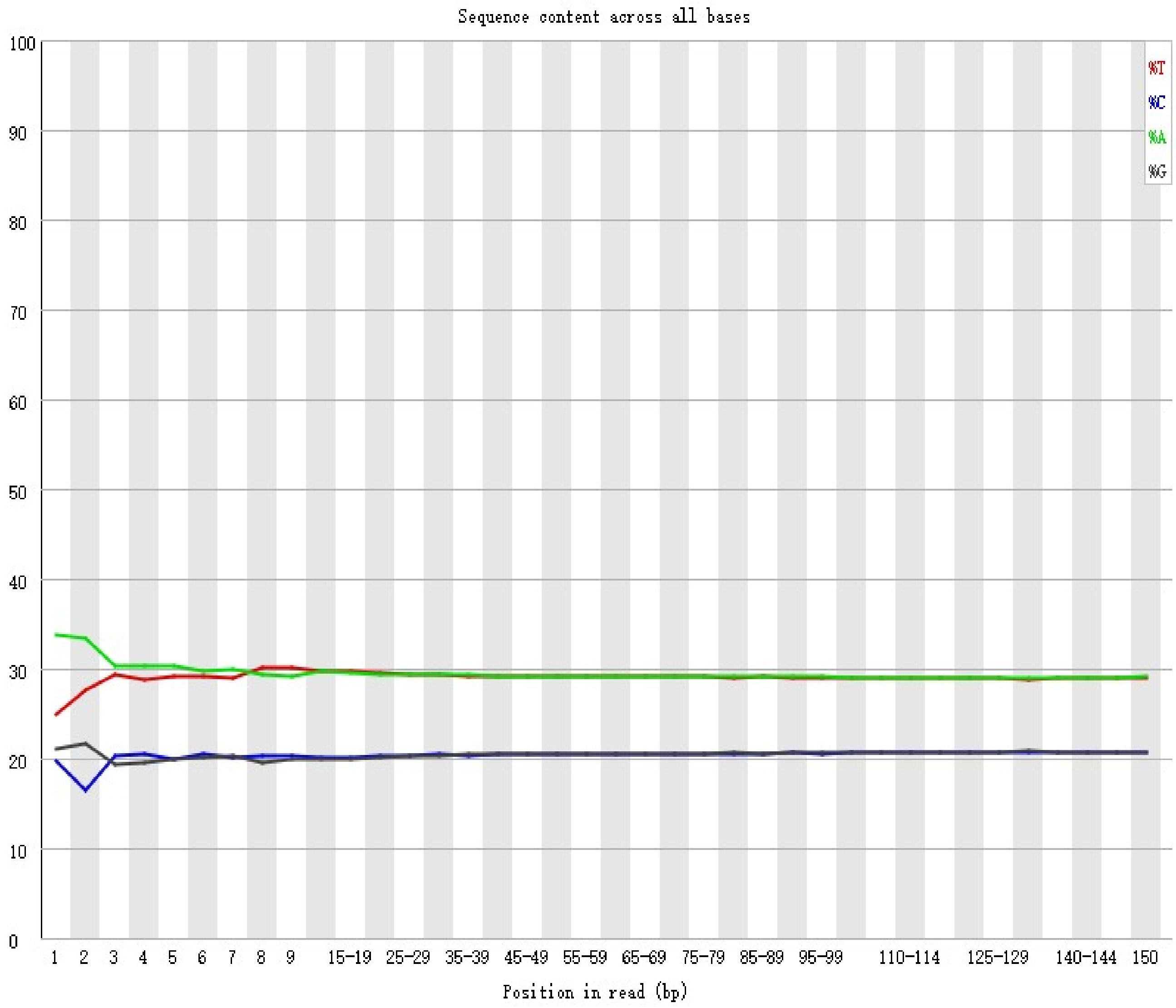

Per sequence GC content

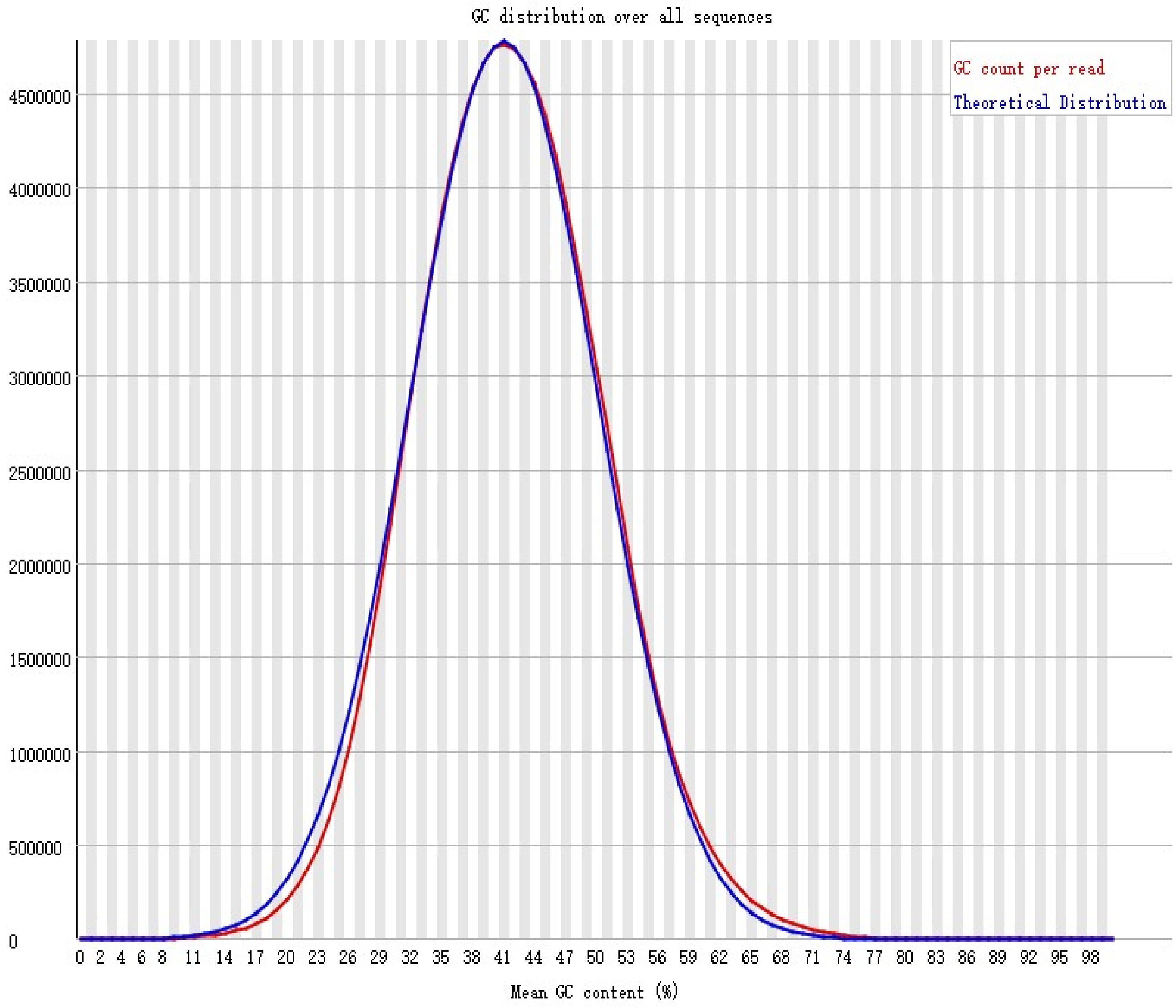

Per base N content

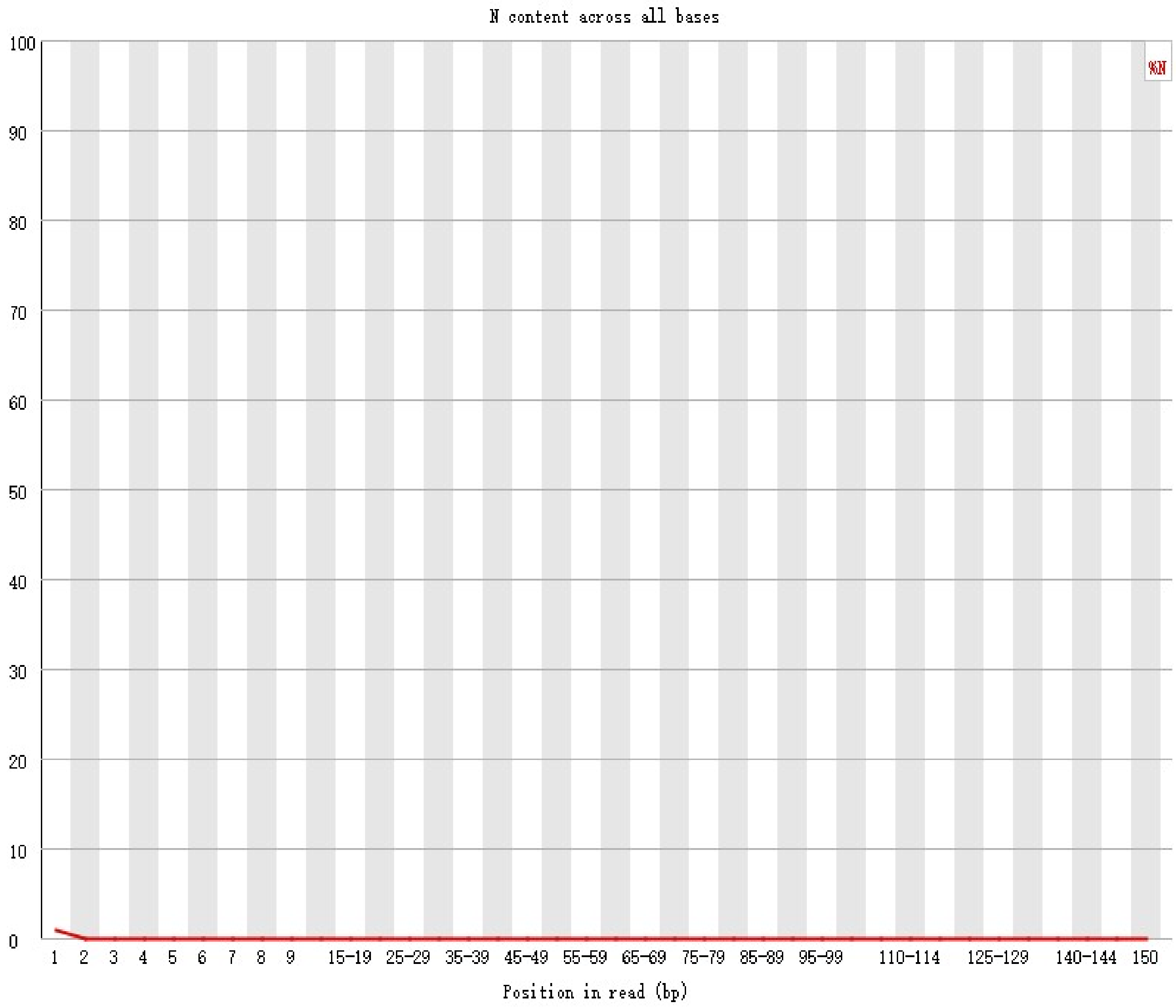

Sequence Length Distribution

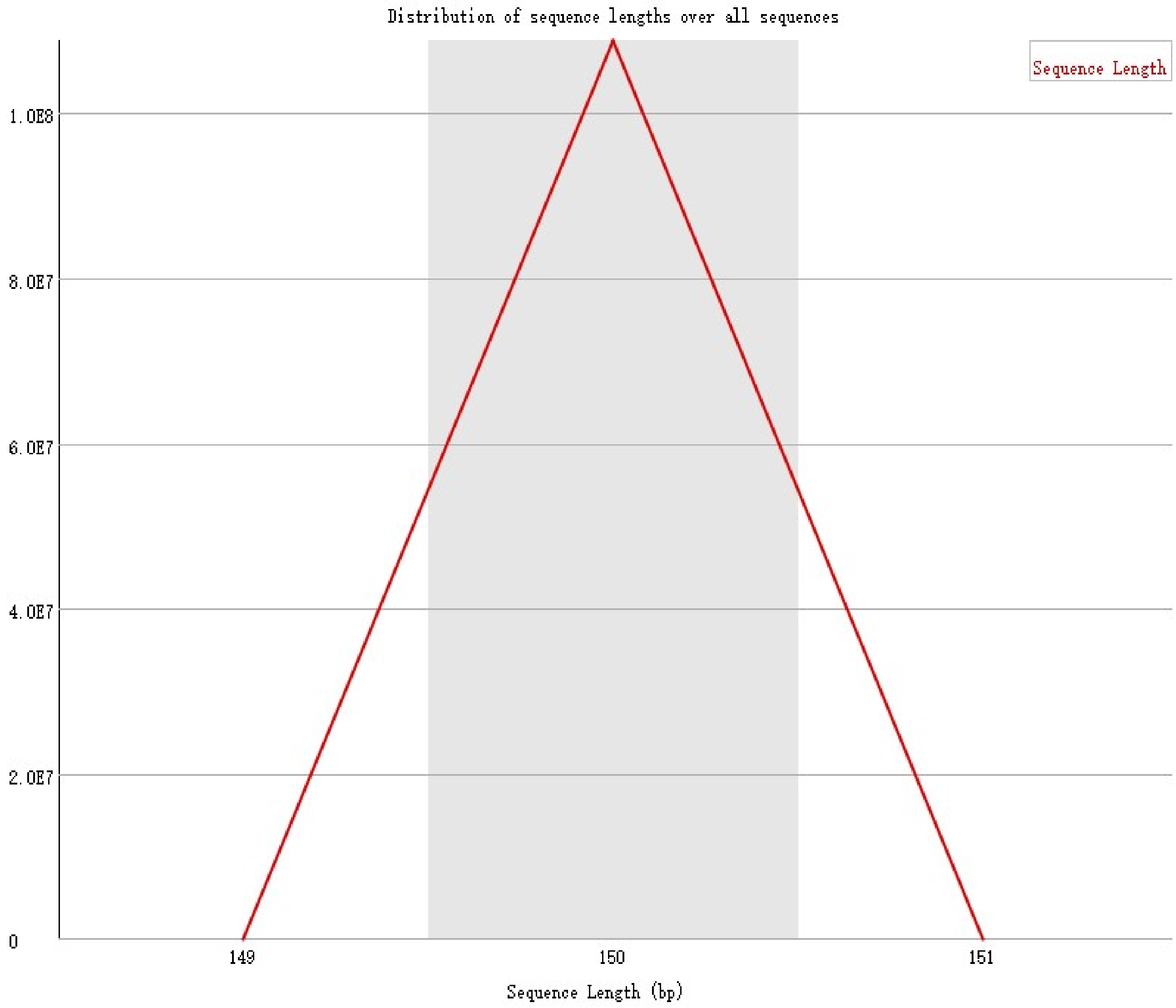

Sequence Duplication Levels

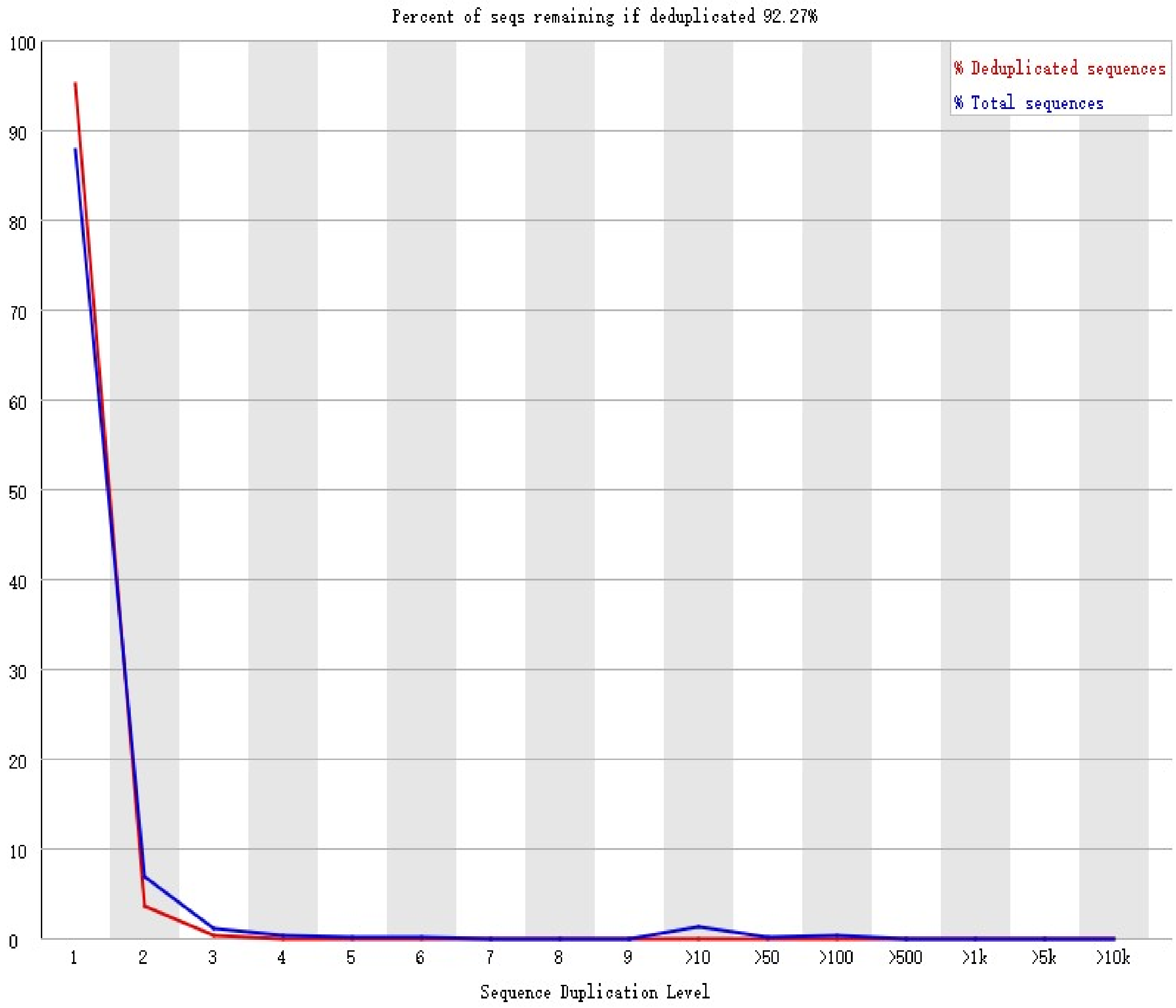

Overrepresented sequences

No overrepresented sequences

Adapter Content

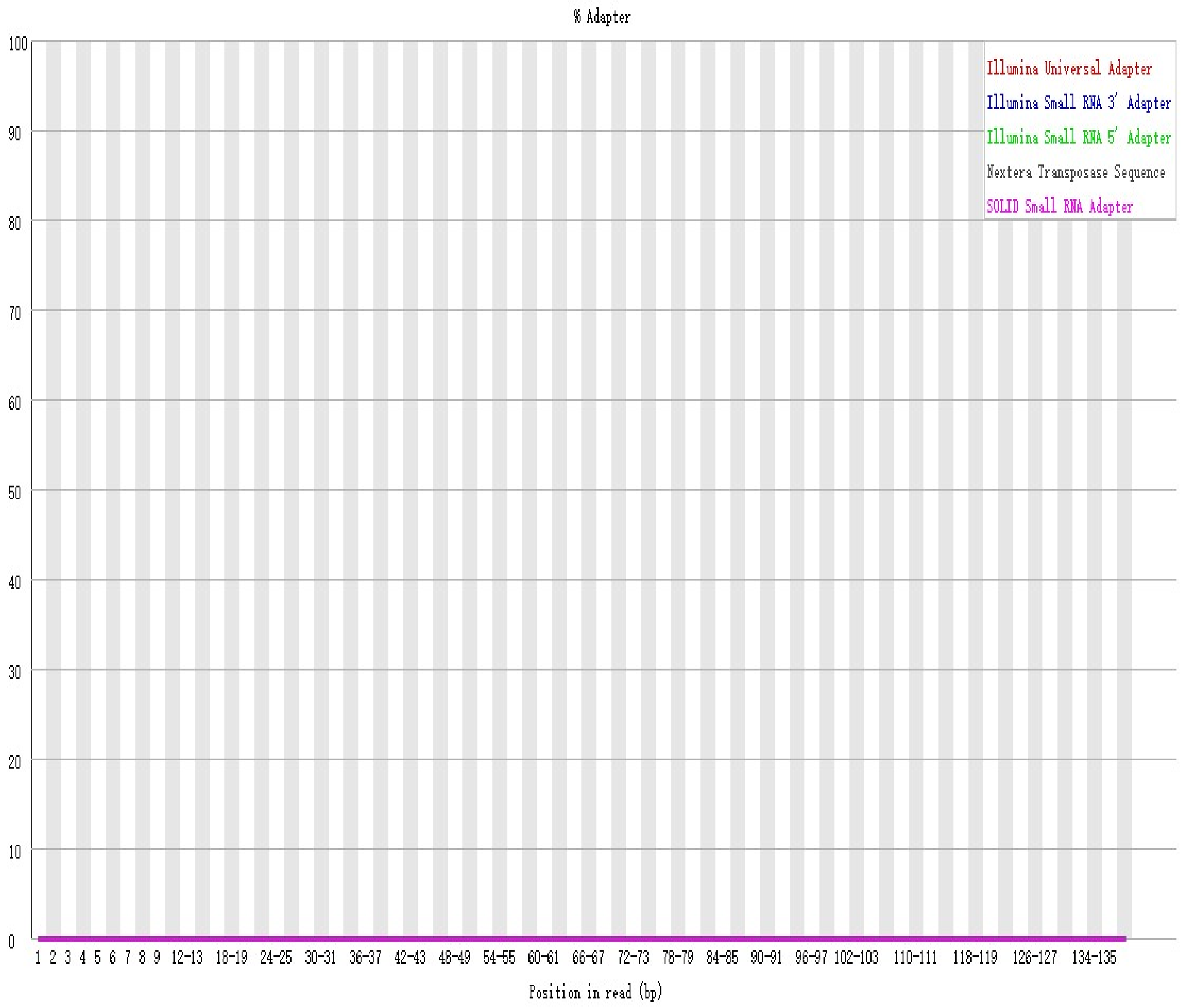

Kmer Content

No overrepresented Kmers

Produced by FastQC (version 0.11.5)

FastQC Report

星期三 8 五月 2019
SN_2_clean.rd.fq.gz

Summary

- Basic Statistics
- Per base sequence quality
- Per tile sequence quality
- Per sequence quality scores
- Per base sequence content
- Per sequence GC content
- Per base N content
- Sequence Length Distribution
- Sequence Duplication Levels
- Overrepresented sequences
- Adapter Content
- Kmer Content

Basic Statistics

Measure	Value
Filename	SN_2_clean.rd.fq.gz
File type	Conventional base calls
Encoding	Sanger / Illumina 1.9
Total Sequences	108737330
Sequences flagged as poor quality	0
Sequence length	150
%GC	41

Per base sequence quality

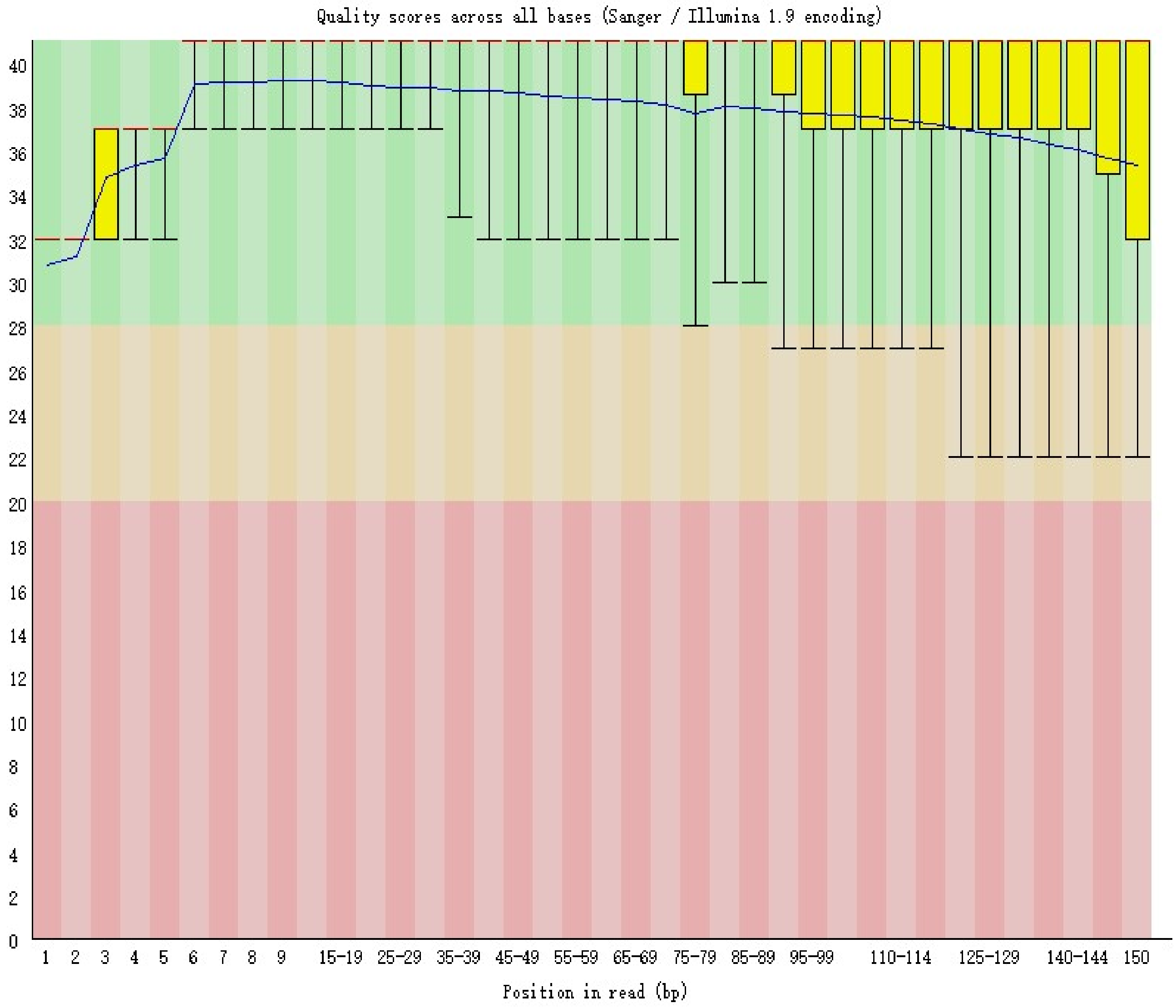

Per tile sequence quality

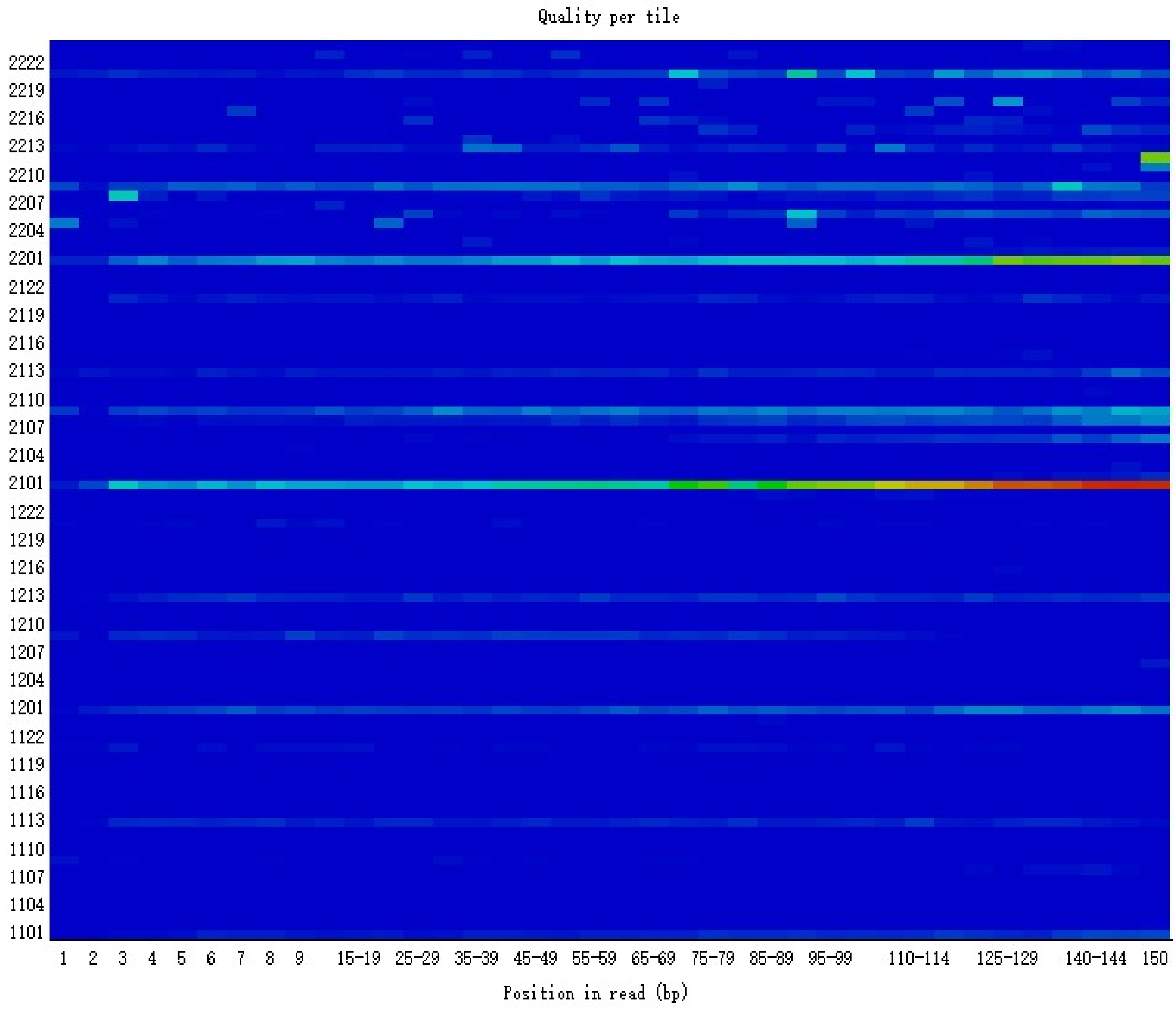

Per sequence quality scores

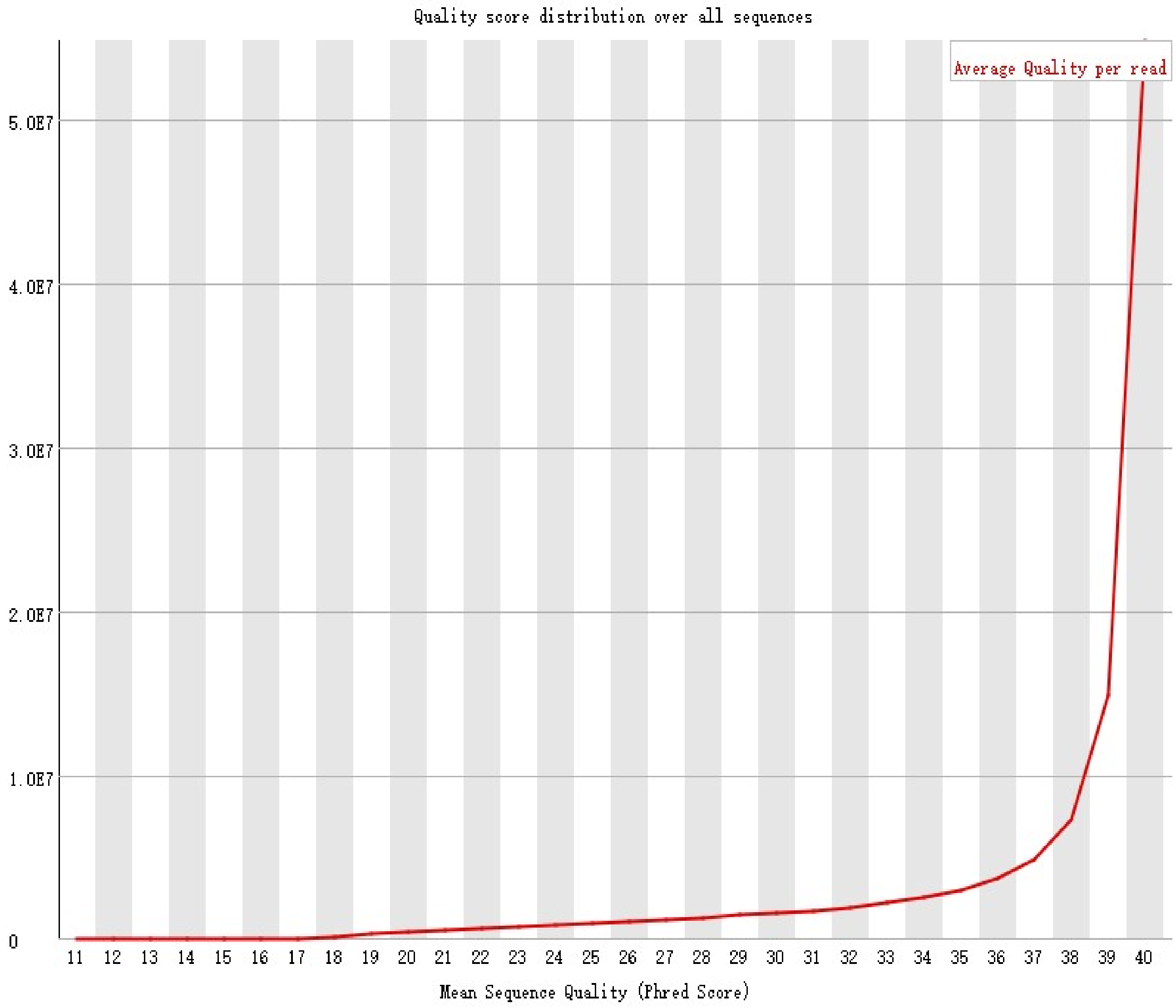

Per base sequence content

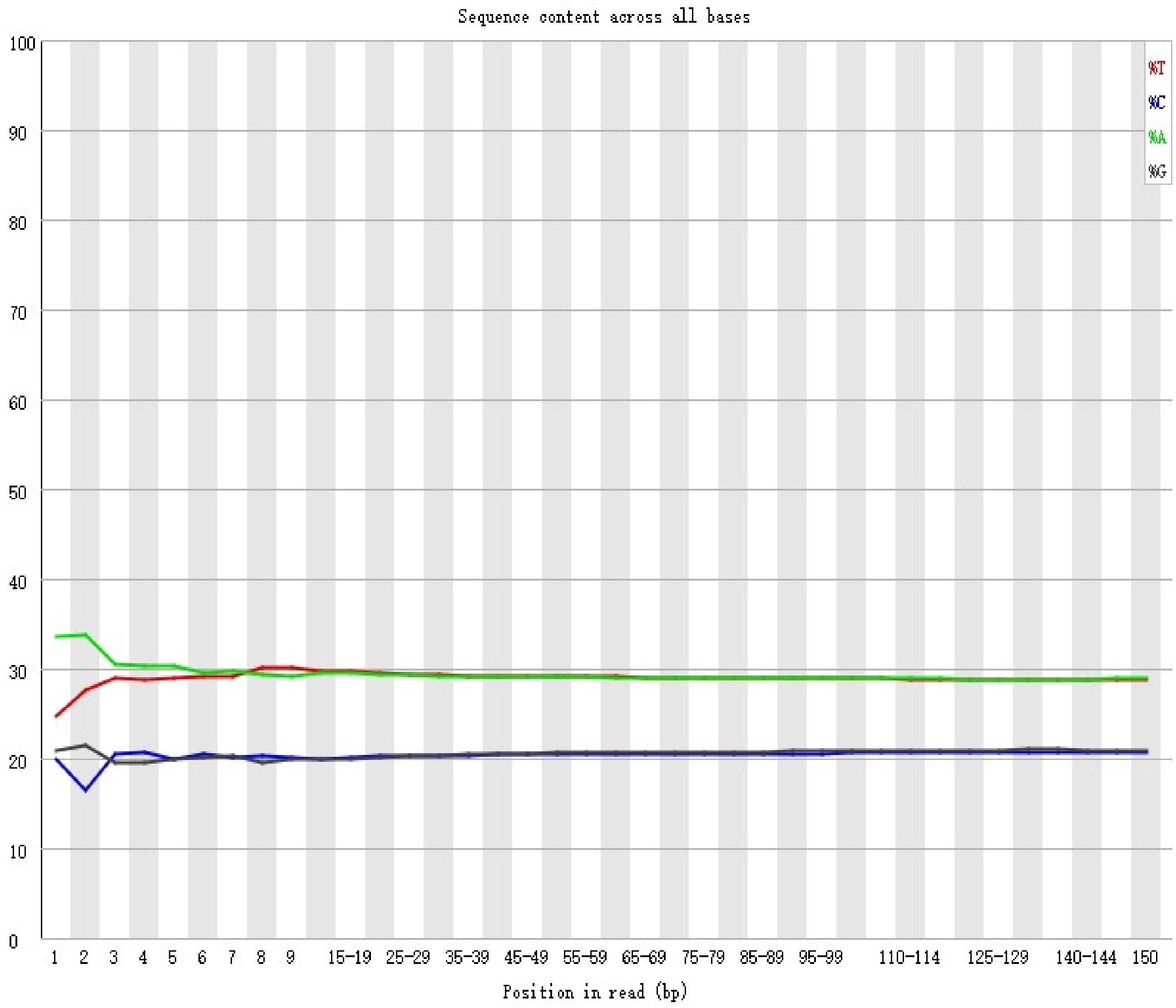

Per sequence GC content

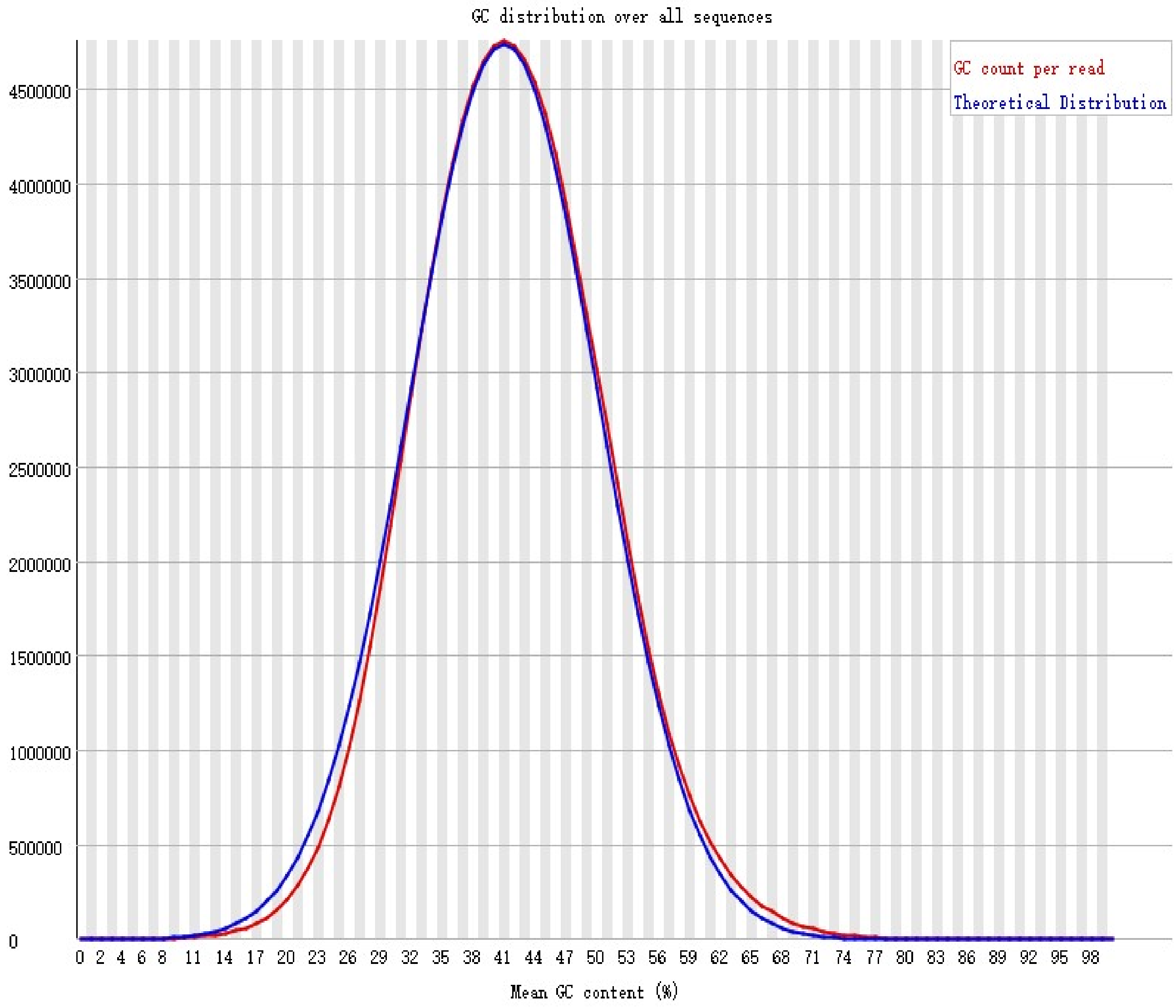

Per base N content

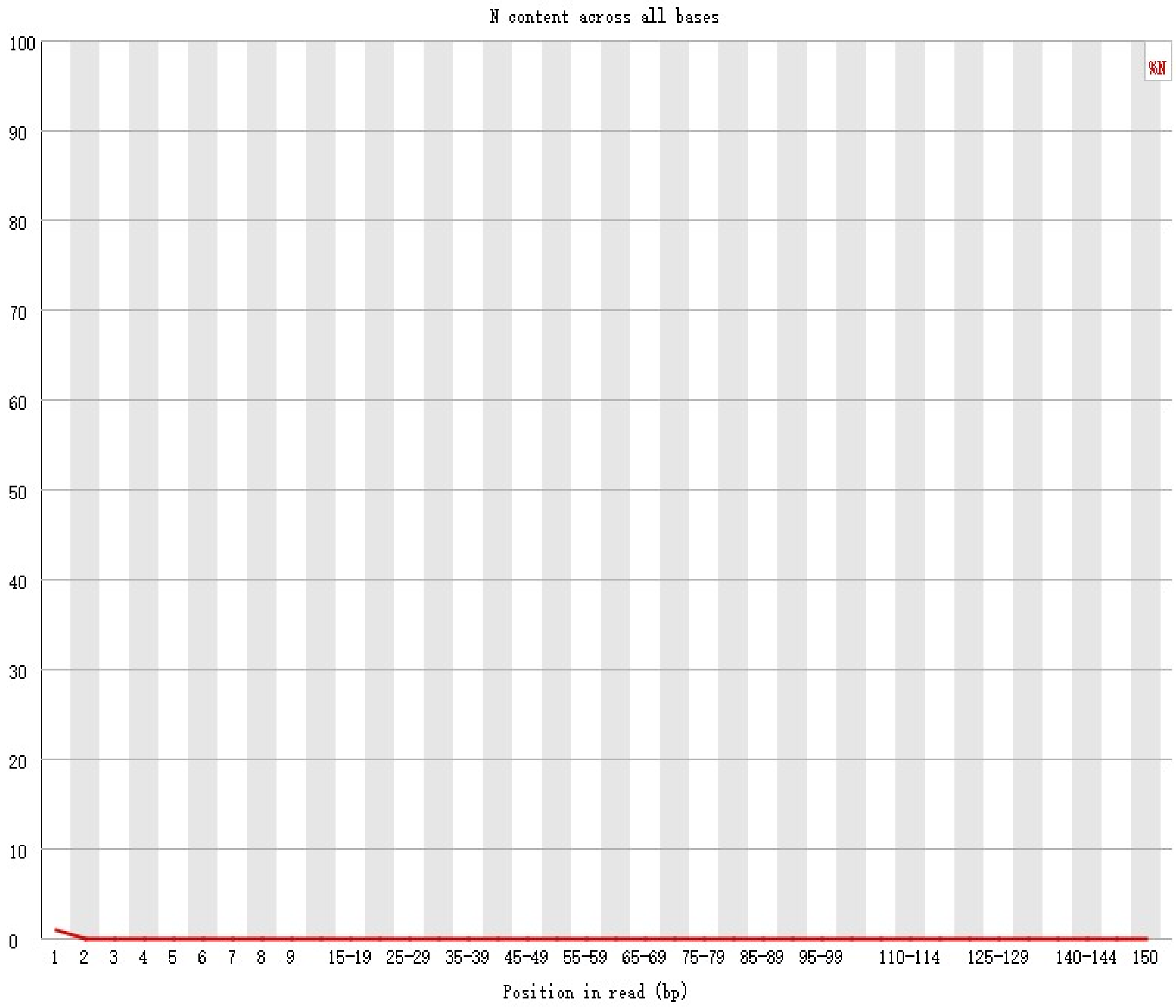

Sequence Length Distribution

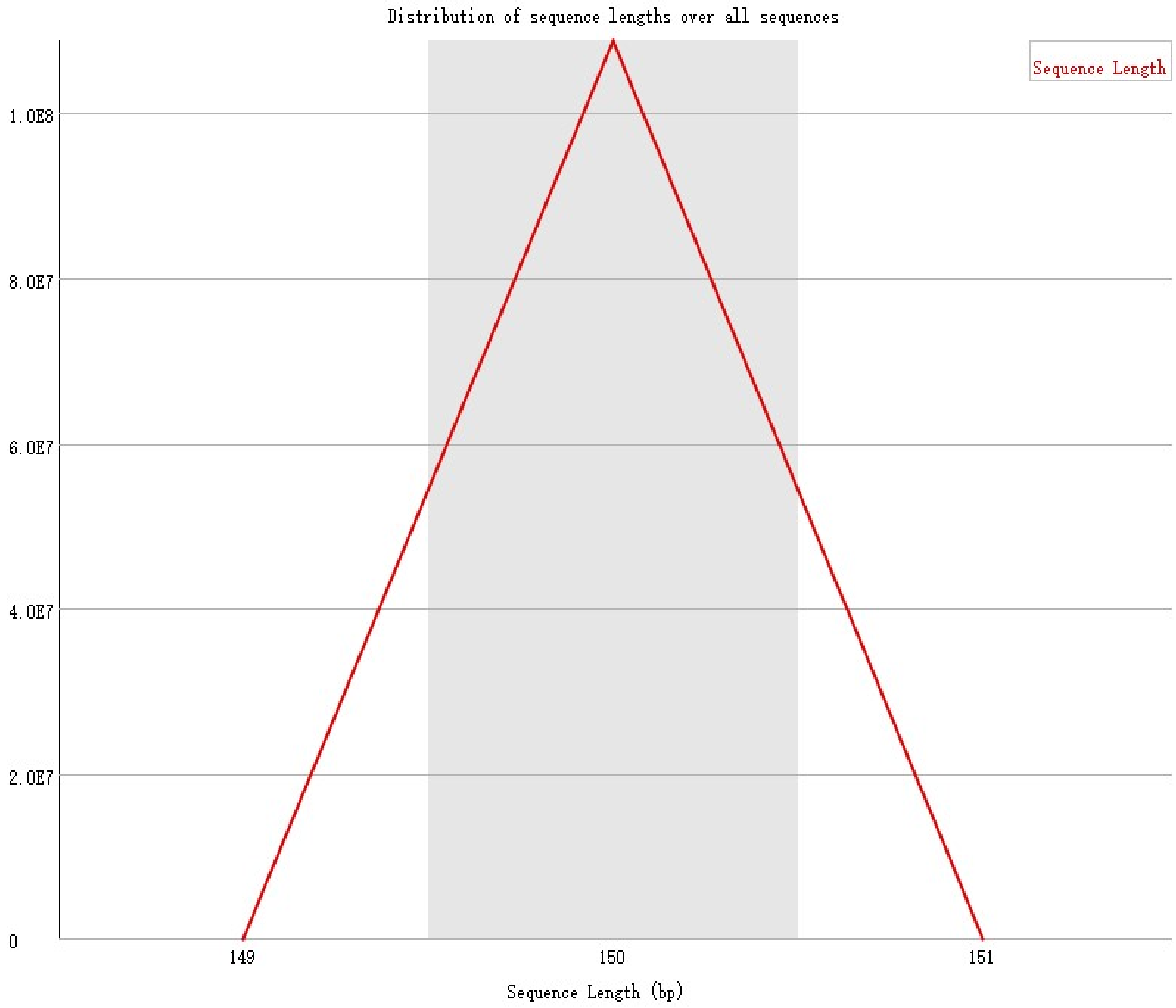

Sequence Duplication Levels

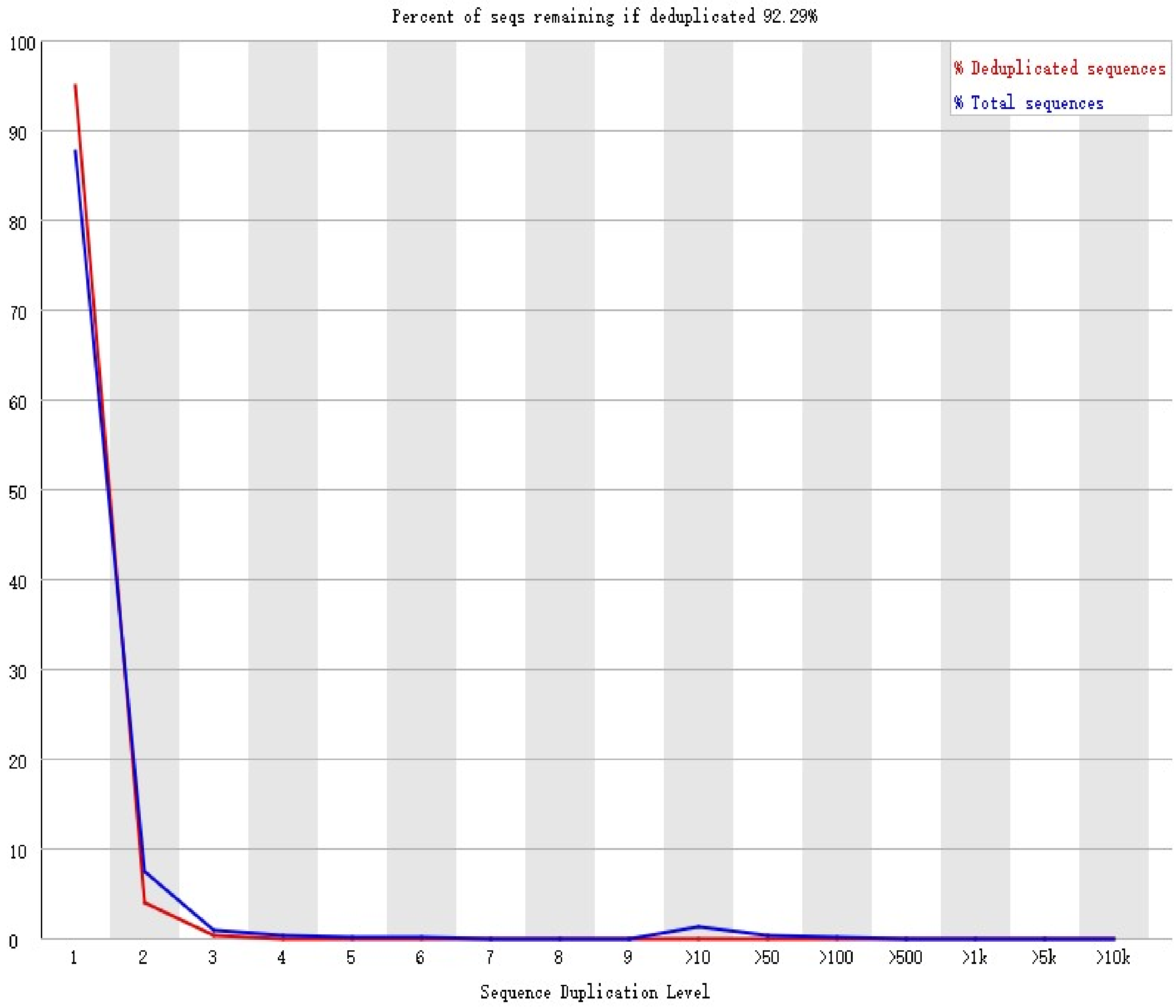

Overrepresented sequences

No overrepresented sequences

Adapter Content

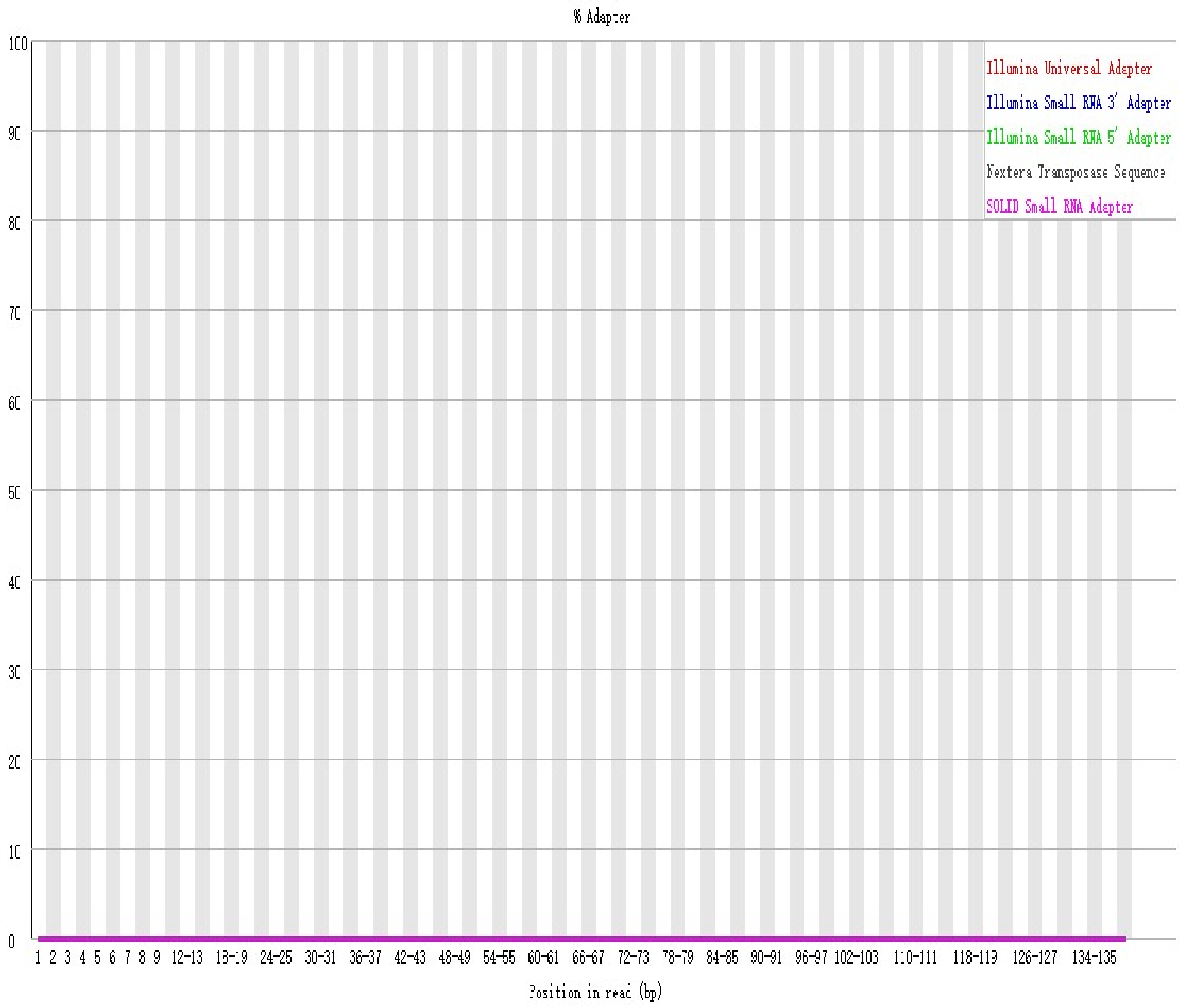

Kmer Content

No overrepresented Kmers

Produced by FastQC (version 0.11.5)
